# Boron-deficiency-responsive microRNAs and their targets in *Citrus sinensis* leaves

**DOI:** 10.1186/s12870-015-0642-y

**Published:** 2015-11-04

**Authors:** Yi-Bin Lu, Yi-Ping Qi, Lin-Tong Yang, Peng Guo, Yan Li, Li-Song Chen

**Affiliations:** College of Resource and Environmental Science, Fujian Agriculture and Forestry University, Fuzhou, 350002 China; Institute of Horticultural Plant Physiology, Biochemistry and Molecular Biology, Fujian Agriculture and Forestry University, Fuzhou, 350002 China; Institute of Materia Medica, Fujian Academy of Medical Sciences, Fuzhou, 350001 China; The Higher Educational Key Laboratory of Fujian Province for Soil Ecosystem Health and Regulation, Fujian Agriculture and Forestry University, Fuzhou, 350002 China; Fujian Key Laboratory for Plant Molecular and Cell Biology, Fujian Agriculture and Forestry University, Fuzhou, 350002 China

**Keywords:** Boron-deficiency, *Citrus sinensis*, Illumina sequencing, Leaves, MicroRNA

## Abstract

**Background:**

MicroRNAs play important roles in the adaptive responses of plants to nutrient deficiencies. Most research, however, has focused on nitrogen (N), phosphorus (P), sulfur (S), copper (Cu) and iron (Fe) deficiencies, limited data are available on the differential expression of miRNAs and their target genes in response to deficiencies of other nutrient elements. In this study, we identified the known and novel miRNAs as well as the boron (B)-deficiency-responsive miRNAs from citrus leaves in order to obtain the potential miRNAs related to the tolerance of citrus to B-deficiency.

**Methods:**

Seedlings of ‘Xuegan’ [*Citrus sinensis* (L.) Osbeck] were supplied every other day with B-deficient (0 μM H_3_BO_3_) or -sufficient (10 μM H_3_BO_3_) nutrient solution for 15 weeks. Thereafter, we sequenced two small RNA libraries from B-deficient and -sufficient (control) citrus leaves, respectively, using Illumina sequencing.

**Results:**

Ninety one (83 known and 8 novel) up- and 81 (75 known and 6 novel) down-regulated miRNAs were isolated from B-deficient leaves. The great alteration of *miRNA* expression might contribute to the tolerance of citrus to B-deficiency. The adaptive responses of miRNAs to B-deficiency might related to several aspects: (a) attenuation of plant growth and development by repressing auxin signaling due to decreased *TIR1* level and ARF-mediated gene expression by altering the expression of *miR393*, *miR160* and *miR3946*; (b) maintaining leaf phenotype and enhancing the stress tolerance by up-regulating *NACs* targeted by *miR159*, *miR782*, *miR3946* and *miR7539*; (c) activation of the stress responses and antioxidant system through down-regulating the expression of *miR164*, *miR6260*, *miR5929*, *miR6214*, *miR3946* and *miR3446*; (d) decreasing the expression of *major facilitator superfamily protein genes* targeted by miR5037, thus lowering B export from plants. Also, B-deficiency-induced down-regulation of *miR408* might play a role in plant tolerance to B-deficiency by regulating Cu homeostasis and enhancing superoxide dismutase activity.

**Conclusions:**

Our study reveals some novel responses of citrus to B-deficiency, which increase our understanding of the adaptive mechanisms of citrus to B-deficiency at the miRNA (post-transcriptional) level.

**Electronic supplementary material:**

The online version of this article (doi:10.1186/s12870-015-0642-y) contains supplementary material, which is available to authorized users.

## Background

Boron (B), an essential micronutrient for normal growth and development of plants, is involved in a series of important physiological functions, including the structure of cell walls, membrane integrity, cell division, phenol metabolism, protein metabolism and nucleic acid metabolism during growth and development of higher plants [1–5]. B-deficiency widespreadly exists in many agricultural crops, including citrus. In China, B-deficiency is frequently observed in citrus orchards, and often contributes to the loss of productivity and poor fruit quality [3]. Li et al. reported that up to 9.0 % and 43.5 % of ‘Guanximiyou’ pummelo (*Citrus grandis*) orchards in Pinghe, Zhangzhou, China were deficient in leaf B and soil water-soluble B, respectively [6].

In plants, approx. 21-nucleotide-long microRNAs (miRNAs), one of the most abundant classes of non-coding small RNAs (sRNAs), are crucial post-transcriptional regulators of gene expression by repressing translation or directly degrading mRNAs in plants [7]. Evidence shows that miRNAs play key roles in plant response to nutrient deficiencies [8–13]. Identification of nutrient-deficiency-responsive-miRNAs and their target genes has become one of the hottest topics in plant nutrition.

Plants have developed diverse strategies to maintain phosphorus (P) homeostasis, including miRNA regulations [11, 12]. *MiR399*, which is specifically induced by P-deficiency in *Arabidopsis* and rice, can regulate P homeostasis by negatively regulating its target gene *UBC24* [13, 14]. Like *miR399*, *miR827* is also highly and specifically induced by P-deficiency and is involved in the regulation of plant P homeostasis by down-regulating its target gene *nitrogen limitation adaptation* (*NLA*) in *Arabidopsis* [13]. In addition, many other P-deficiency-responsive miRNAs (i.e., miR1510, miR156, miR159, miR166, miR169, miR2109, miR395, miR397, miR398, miR408, miR447 and miR482) have been isolated from various plant species [15–21].

*MiR397*, *miR398*, *miR408*, and *miR857*, which are induced by copper (Cu)-deficiency, have been shown to play a role in the regulation of Cu homeostasis by down-regulating genes encoding nonessential Cu proteins such as Cu/Zn superoxide dismutase (SOD), laccases and plantacyanin, hence saving Cu for other essential Cu proteins such as plastocyanin, which is essential for photosynthesis [10, 22, 23].

In *Arabidopsis*, leaf *miR395* was induced by sulfur (S)-deficiency. MiR395 targets *ATP sulfurylases* (*APS*) and *sulfate transporter 2;1* (*SULTR2;1*), both of which are involved in the S metabolism. Their transcripts are greatly down-regulated in *miR395*-over-expressing transgenic *Arabidopsis* accompanied by increased accumulation of S in the shoot but not in the root. They concluded that miR395 play a role in the regulation of plant S accumulation and allocation by targeting *APS* and *SULTR2;1* [24].

MiRNAs have been shown to play a role in the adaptation of plants to Fe-deficiency. Eight Fe-deficiency-responsive conserved miRNAs from five families had been identified in *Arabidopsis* roots and shoots and their expression profiles differed between the two organs [25]. Valdés-López et al. isolated ten up- and four down-regulated miRNAs, five up- and six down-regulated miRNAs, and seven up- and four down-regulated miRNAs from the leaves, roots and nodules of Fe-deficient common bean [17]. Waters et al. obtained eight differentially expressed miRNAs from seven conserved families in the rosettes of Fe-deficient *Arabidopsis*. Interestingly, Fe-deficiency led to increased accumulation of Cu in rosettes and decreased expression levels of *miR397a, miR398a* and *miR398b/c*, which regulate the mRNA levels of genes encoding Cu-containing proteins, implying a links between Fe-deficiency with Cu homeostasis [26].

Many N-deficiency-responsive miRNAs have been identified from *Arabidopsis*, soybean, maize and common bean. These miRNAs belong to at least 27 conserved families [10, 17, 27, 28]. In *Arabidopsis*, the expression of *miR169* was inhibited by N-deficiency, while the expression levels of its target genes [i.e., *NFYA2* (*Nuclear Factor Y, subunit A2)*, *NFYA3, NFYA5* and *NFYA8*] were increased [10, 13, 27, 29]. Transgenic *Arabidopsis* plants over-expressing *miR169a* had less accumulation of N and *NFYA* family members, and were more sensitive to N stress than the wild type, demonstrating a role for miR169 in the adaptation of plants to N-deficiency [29]. It is worth noting that some N-deficiency-responsive miRNAs (e.g., miR169, miR172, miR394, miR395, miR397, miR398, miR399, miR827, miR408 and miR857) are also responsive to other nutrient stresses (i.e., B, P, Fe, S and Cu deficiencies) in plants [8, 10], indicating the involvement of miRNA-mediated crosstalk among N, B, P, Fe, S and Cu under N-deficiency.

An increasing number of nutrient-deficiency-responsive miRNAs have been identified with different techniques [8–14]. Most research, however, has focused on N, P, S, Cu and Fe deficiencies, limited data are available on the differential expression of miRNAs and their target genes in response to deficiencies of other nutrient elements. Recently, we investigated miRNA expression profiles in response to B-deficiency in *Citrus sinensis* roots by Illumina sequencing and identified 134 (112 known and 22 novel) B-deficiency-responsive miRNAs, suggesting the possible roles of miRNAs in the tolerance of citrus plants to B-deficiency [8]. Previous studies showed that the responses of miRNAs to nutrient deficiencies differed between plant roots and shoots (leaves) [12, 17, 25]. In addition, there were great differences in B-deficiency-induced changes in major metabolites, activities of key enzymes involved in organic acid and amino acid metabolism, gas exchange and gene expression profiles between roots and leaves of *C. sinensis* [4, 30]. Therefore, B-deficiency-induced changes in miRNA expression profiles should be different between citrus roots and leaves.

In this study, we sequenced two small RNA libraries from B-deficient and -sufficient (control) citrus leaves, respectively, using Illumina sequencing, then identified the known and novel miRNAs as well as the B-deficiency-responsive miRNAs. Also, we predicted the target genes of these known and novel B-deficiency-responsive miRNAs and discussed their possible roles in the response to B-deficiency in citrus. The objective of this study is to identify the potential miRNAs related to the tolerance of citrus to B-deficiency.

## Results

### B and Cu concentrations in leaves

**Fig. 1:**
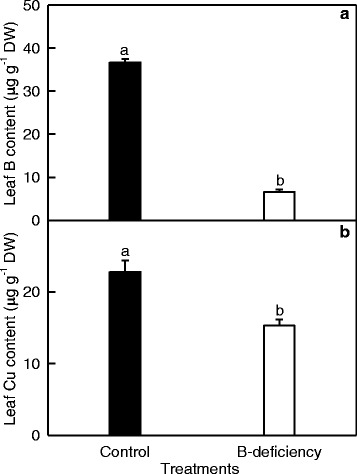
Effects of B-deficiency on B and Cu concentration in leaves. Bars represent mean ± SE (*n* = 3). Different letters above the bars indicate a significant difference at *P* < 0.05

B concentration in 10 μM B-treated leaves was in the sufficient range of 30 to 100 μg g^−1^ DW, while the value in 0 μM B-treated leaves was much less than 30 μg g^−1^ DW (Fig. 1a) [31]. Visible B-deficient symptoms were observed only in 0 μM B-treated leaves (data not shown). Therefore, seedlings treated with 0 μM B are considered as B-deficient, and those treated with 10 μM B are considered as B-sufficient. B-deficiency decreased leaf concentration of Cu (Fig. 1b).

### Sequencing and analysis of two small RNA libraries from B-sufficient and -deficient citrus leaves

As shown in Table 1, 17,996,827 and 18,223,948 raw reads were generated from the libraries of B-sufficient and -deficient leaves, respectively. After removal of the contaminant reads like adaptors and low quality tags, 17,597,008 and 17,829,966 clear reads were obtained from the libraries of B-sufficient and -deficient leaves, comprising 3,673,054 and 4,654,829 unique clear reads, respectively. Among these reads, 11,726,078 clean reads (1,961,407 unique reads) from B-sufficient leaves and 11,372,875 clean reads (2,484,833 unique reads) from B-deficient leaves were mapped to *C. sinensis* genome (JGIversion 1.1, http://phytozome.jgi.doe.gov/pz/portal.html#!info?alias=Org_Csinensis) using SOAP [32]. Exon, intron, miRNA, rRNA, repeat regions, snRNA, snoRNA and tRNA reads were annotated, respectively. After removal of these annotated reads, the remained unique reads that were used to predict novel miRNAs for B-sufficient and -deficient leaves were 3,237,407 and 4,179,224 reads, respectively.

**Table 1:** Statistical analysis of sRNA sequencing data from B-sufficient and -deficient leaves of *Citrus sinensis*

	B-sufficiency		B-deficiency	
	Unique sRNAs	Total sRNAs	Unique sRNAs	Total sRNAs
Raw reads		17,996,827		18,223,948
Clear reads	3,673,054 (100 %)	17,597,008 (100 %)	4,654,829 (100 %)	17,829,966 (100 %)
Mapped to genomic	1,961,407 (53.40 %)	11,726,078 (66.64 %)	2,484,833 (53.38 %)	11,372,875 (63.79 %)
Exon antisense	28,626 (0.78 %)	134,009 (0.76 %)	42,754 (0.92 %)	157,929 (0.89 %)
Exon sense	77,868 (2.12 %)	281,505 (1.60 %)	81,887 (1.76 %)	287,483 (1.61 %)
Intron antisense	36,541 (0.99 %)	244,148 (1.39 %)	46,940 (1.01 %)	248,094 (1.39 %)
Intron sense	56,020 (1.53 %)	526,848 (2.99 %)	67,594 (1.45 %)	457,839 (2.57 %)
miRNA	44,496 (1.21 %)	3,858,007 (21.92 %)	46,800 (1.01 %)	2,639,999 (14.81 %)
rRNA	164,311 (4.47 %)	3,052,914 (17.35 %)	158,009 (3.39 %)	2,851,216 (15.99 %)
repeat	821 (0.02 %)	2009 (0.01 %)	1014 (0.02 %)	2718 (0.02 %)
snRNA	2420 (0.07 %)	8040 (0.05 %)	3547 (0.08 %)	10,269 (0.06 %)
snoRNA	1167 (0.03 %)	3628 (0.02 %)	1270 (0.03 %)	4748 (0.03 %)
tRNA	23,377 (0.64 %)	810,902 (4.61 %)	25,790 (0.55 %)	722,780 (4.05 %)
Unannotated sRNAs	3,237,407 (88.14 %)	8,674,998 (49.30 %)	4,179,224 (89.78 %)	10,446,891 (58.59 %)

Most of the clear sequences were within the range of 19–26 nt, which accounted for 89 % of the total clear reads. Reads with the length of 24 nt were at the most abundant, followed by the reads with the length of 21, 22, 23 and 20 nt (Additional file 1). Overall, the size distribution of sRNAs agrees with the results obtained on roots of *Citrus sinensis* [8]*,* fruits of *C. sinensis* [33] and *Citrus trifoliata*, and flowers of *C. trifoliate* [34]. This indicates that the data of sRNA libraries obtained by the Illumina sequencing are reliable.

### Identification of known and novel miRNAs in citrus leaves

Here, a total of 734 known miRNAs were isolated from the two libraries (Additional file 2). The count of reads was normalized to transcript per million (TPM) in order to compare the abundance of miRNAs in the two libraries. The most abundant miRNA isolated from B-sufficient and -deficient libraries was miR157 (86,829.4201 and 48,091.4546 TPM, respectively), followed by miR166 (36,979.7525 and 26148.2271 TPM, respectively) and miR167 (24,944.5815 and 16,269.745, respectively). In this study, only these known miRNAs with normalized read-count more than ten TPM in B-sufficient and/or -deficient leaf libraries were used for further analysis in order to avoid false results caused by the use of low expressed miRNAs [8, 35]. After removal of these low expressed miRNAs, the remained 321 known miRNAs were used for further analysis (Additional file 3).

After removal of these annotated reads (i.e., exon, intron, miRNA, rRNA, repeat regions, snRNA, snoRNA and tRNA), the remained 3,237,407 and 4,179,224 reads from B-sufficient and -deficient libraries, respectively were used to predict novel miRNAs using the Mireap (http://sourceforge.net/projects/mireap/). Based on the criteria for annotation of plant miRNAs [7, 36], a total of 71 novel miRNAs were isolated from the two libraries (Additional file 4). Like the known miRNAs, novel miRNAs with normalized read-count less than ten TPM were not included in the expression analysis [7, 35]. After excluding these low expressed novel miRNAs, the remained 28 miRNAs were used for further analysis (Additional file 5).

### Identification of B-deficiency-responsive miRNAs in citrus leaves

We identified 91 (83 known and 8 novel) up- and 81 (75 known and 6 novel) down-regulated miRNAs from B-deficient leaves. The most pronounced up- and down-regulated known (novel) miRNAs were miR5266 with a fold-change of 16.22 (novel_miR_95 with a fold-change of 17.61) and miR401 with a fold-change of −15.87 (novel_miR_236 with a fold-change of −18.48), respectively (Additional files 3 and 5).

### Validation of high-throughput sequencing results by qRT-PCR

**Fig. 2:**
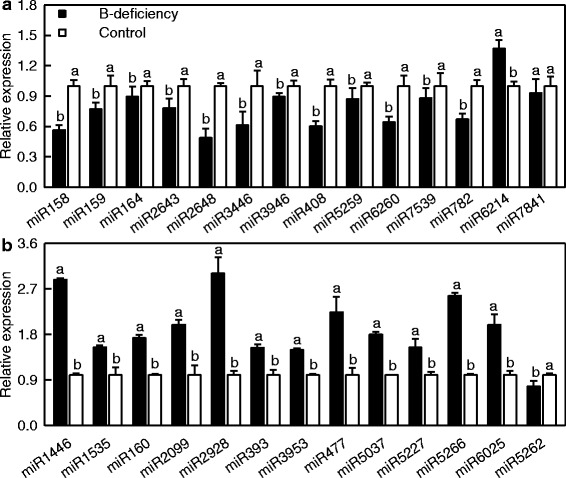
Relative abundances of selected known miRNAs in B-deficient and control leaves revealed by qRT-PCR. Bars represent mean ± SD (*n* = 3). Significant differences were tested between control and B-deficient leaves for the same miRNA. Different letters above the bars indicate a significant difference at *P* < 0.05. All the values were expressed relative to the control leaves

We analyzed the expression of 27 known miRNAs using stem-loop qRT-PCR in order to validate the *miRNA* expression patterns revealed by Illumina sequencing. The expression levels of all these miRNAs except for *miR6214*, *miR5262* and *miR7841* were comparable in magnitude to the expression patterns obtained by Illumiona sequencing (Fig. 2). Obviously, the high-throughput sequencing allowed us to identify the differentially expressed *miRNAs* under B-deficiency.

### Identification of targets for differentially expressed miRNAs and GO analysis

**Fig. 3:**
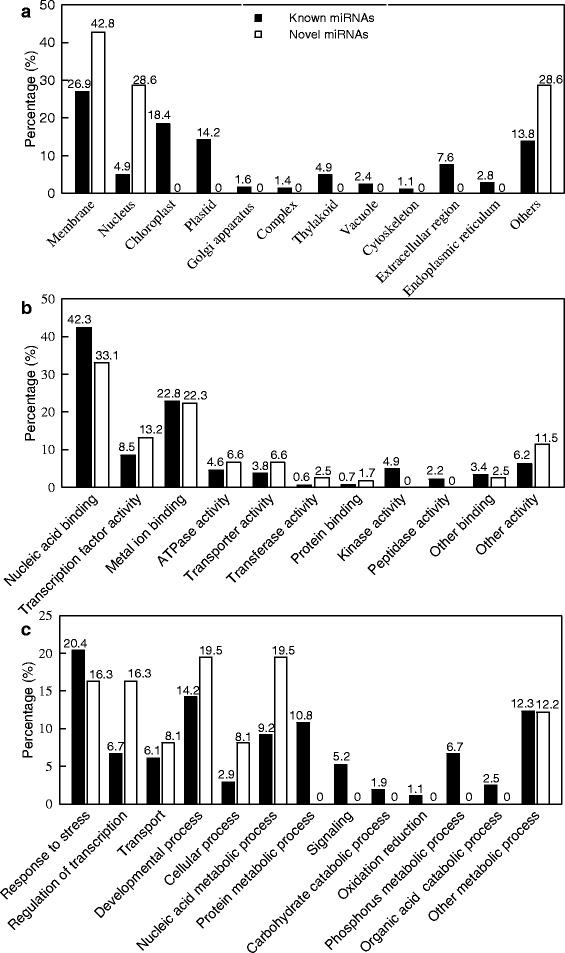
GO of the predicted target genes for 70 (6) differentially expressed known (novel) miRNAs. Categorization of miRNAs target genes was performed according to cellular component (**a**), molecular function (**b**) and biological process (**c**)

In this study, we predicted 489 and 17 target genes from the 70 known and 6 novel differentially expressed miRNAs, respectively (Additional files 6 and 7). GO categories were assigned to all these target genes based on the cellular component, molecular function and biological process. These target genes for the known and novel miRNAs were related to 12 and 3 components, respectively based on the cellular component. The most three GO terms for known miRNAs were membrane, chloroplast and plastid, while more than 42 % of the target genes for novel miRNAs belonged to membrane (Fig. 3a). Based on the molecular function, the target genes for the known and novel miRNAs genes were grouped into 11 and 9 categories, respectively, the highest percentage of three categories were nucleic acid binding, metal ion binding and transcription factor activity (Fig. 3b). In the biological process, the target genes were mainly focused on response to stress and developmental process for known miRNAs, and nucleic acid metabolic process, developmental process, response to stress and regulation of transcription for novel miRNAs, respectively (Fig. 3c).

### qRT-PCR validation of target genes

To verify the expression of the target genes and how the miRNAs regulate their target genes, 77 genes targeted by 14 down- and 13 up-regulated miRNAs were assayed by qRT-PCR (Table 2). Among the 77 genes, the expression changes of 58 target genes showed a negative correlation with their corresponding miRNAs, implying that miRNAs might play a role in regulating gene expression under B-deficiency by cleaving mRNAs. However, the expression changes of the remained 19 target genes had a positive correlation with their corresponding miRNAs, which might be the results of the interaction of different target genes.

**Table 2:** qRT-PCR relative expression of experimentally determined or predicted target genes of selected miRNAs

miRNA	Fold change of miRNA	Accession	Homology	Target genes	Relative change of target genes
miR158	−3.35603222**	**orange1.1g022993m**	**AT1G69840.1**	**SPFH/Band 7/PHB domain-containing membrane-associated protein family**	1.9490**
			**AT2G03210**	**Fucosyltransferase 2**	1.6482**
		orange1.1g001709m	AT3G07400	Lipase class 3 family protein	0.7819*
miR159	−2.04145817**	**orange1.1g039708m**	**AT5G06100.2**	**MYB domain protein 33**	1.1319*
		**orange1.1g044979m**	**AT4G27330.1**	**Sporocyteless (SPL)**	2.2016**
		**orange1.1g046419m**	**AT4G26930.1**	**MYB domain protein 97**	1.9078**
		orange1.1g011938m	AT3G11440.1	MYB domain protein 65	0.8778**
		**orange1.1g038795m**	**AT3G60460.1**	**MYB-like HTH transcriptional regulator family protein**	1.6685**
miR160	1.81653886**	**orange1.1g004896m**	**AT2G28350.1**	**ARF10**	0.7870**
		**orange1.1g005075m**	**AT4G30080.1**	**ARF16**	0.7150**
		**orange1.1g008078m**	**AT1G77850.1**	**ARF17**	0.9153**
miR164	−2.28320824**	orange1.1g030909m	AT1G56010.2	NAC domain containing protein 1	0.5939**
		**orange1.1g047710m**	**AT5G53950.1**	**NAC domain transcriptional regulator superfamily protein**	1.4205**
		**orange1.1g017827m**	**AT5G61430.1**	**NAC domain containing protein 100**	1.3247**
		orange1.1g017636m	AT3G08030.1	Protein of unknown function, DUF642	0.5400**
miR158	−3.35603222**	**orange1.1g022993m**	**AT1G69840.1**	**SPFH/Band 7/PHB domain-containing membrane-associated protein family**	1.9490**
			**AT2G03210**	**Fucosyltransferase 2**	1.6482**
		orange1.1g001709m	AT3G07400	Lipase class 3 family protein	0.7819*
miR393	1.66802767**	**orange1.1g010049m**	**AT3G18080.1**	**B-S glucosidase 44**	0.8384**
		**orange1.1g007916m**	**At3g62980**	**TIR1**	0.7489**
			**At4g03190**	**AFB1**	0.8195**
		**orange1.1g008325m**	**At3g26810**	**AFB2**	0.7895**
			At1g12820	AFB3	1.6782**
miR408	−2.55840249**	**orange1.1g013075m**	**At2g30210**	**Laccase 3**	1.5874**
		orange1.1g041358m	At5g05390	Laccase 12	0.8814**
			**At5g07130**	**Laccase 13**	1.1251*
		**orange1.1g048131m**	**At2g02850**	**Plantacyanin**	1.6723**
miR477	3.82198862**	**orange1.1g018483m**	**AT3G11340.1**	**UDP-Glycosyltransferase superfamily protein**	0.6543**
miR782	−10.08402439**		**HQ202267**	**MYB transcription factor (MYBML2)**	1.5782**
		orange1.1g039969m	NM_001112290	Protein disulfide isomerase (PDIL5-1)	0.9081**
miR1446	5.01671689**	**orange1.1g037028m**	**AT1G14920.1**	**GRAS family transcription factor family protein**	0.7887**
miR1535	1.58529156**	**orange1.1g001616m**	**AT3G63380.1**	**ATPase E1-E2 type family protein/haloacid dehalogenase-like hydrolase family protein**	0.6757**
		**orange1.1g015157m**	**AT3G58060.1**	**Cation efflux family protein**	0.7189**
miR2099	10.31417531**	**orange1.1g017694m**	**AT3G22830.1**	**Heat shock transcription factor A6B**	0.6459**
miR2643	−2.52218131**	orange1.1g018307m	AT1G12500.1	Nucleotide-sugar transporter family protein	0.9924
		**orange1.1g020050m**	**AT5G19890.1**	**Peroxidase superfamily protein**	1.2307**
miR2648	−11.76162602**	**orange1.1g003798m**	**AT5G58460.1**	**Cation/H** ^**+**^ ** exchanger 25**	2.0379**
miR2928	13.58236255**	**orange1.1g007099m**	**AT4G04450.1**	**WRKY family transcription factor**	0.4129**
		orange1.1g014735m	AT4G22070.1	WRKY DNA-binding protein 31	1.4500**
		**orange1.1g016623m**	**AT1G62300.1**	**WRKY family transcription factor**	0.5791**
miR3446	−1.83050087**	**orange1.1g004633m**	**AT5G66850.1**	**Mitogen-activated protein kinase kinase kinase 5**	1.6310**
		**orange1.1g004928m**	**AT2G25930.1**	**Hydroxyproline-rich glycoprotein family protein**	1.3981**
		**orange1.1g036074m**	**AT4G22200.1**	**Potassium transport 2/3**	1.2999**
miR3946	−1.66667782**	orange1.1g029573m	AT5G47370.1	Homeobox-leucine zipper protein 4 (HB-4)/HD-ZIP protein	0.7342*
		**orange1.1g041705m**	**AT4G25980.1**	**Peroxidase superfamily protein**	1.5621**
		**orange1.1g031837m**	**AT1G08830.1**	**Copper/zinc superoxide dismutase 1**	1.6638**
		orange1.1g016997m	AT1G13310.1	Endosomal targeting BRO1-like domain-containing protein	0.5406**
		**orange1.1g014089m**	**AT1G73390.1**	**Endosomal targeting BRO1-like domain-containing protein**	1.3404**
		**orange1.1g027084m**	**AT3G20560.1**	**PDI-like 5-3**	1.0827*
		**orange1.1g017665m**	**AT3G04070.1**	**NAC domain containing protein 47**	1.6886**
		**orange1.1g010076m**	**AT3G54700.1**	**Phosphate transporter 1;7**	1.7862**
		**orange1.1g034408m**	**AT1G33110.1**	**MATE efflux family protein**	1.5697**
		**orange1.1g027612m**	**AT1G04760.1**	**Vesicle-associated membrane protein 726**	1.2270**
		**orange1.1g027026m**	**AT4G27670.1**	**Heat shock protein 21**	1.3134**
		**orange1.1g020124m**	**AT2G01060.1**	**MYB-like HTH transcriptional regulator family protein**	1.7116**
		orange1.1g011938m	AT3G11440.1	MYB domain protein 65	0.8396**
		**orange1.1g005651m**	**AT1G32640.1**	**Basic helix-loop-helix (bHLH) DNA-binding family protein**	1.3806**
		**orange1.1g012387m**	**AT4G00050.1**	**Basic helix-loop-helix (bHLH) DNA-binding superfamily protein**	1.6480**
		**orange1.1g004509m**	**AT2G45290.1**	**Transketolase**	1.1778*
		orange1.1g033760m	AT2G46690.1	SAUR-like auxin-responsive protein family	0.7430**
miR3953	3.80237602**	**orange1.1g016435m**	**AT5G46590.1**	**NAC domain containing protein 96**	0.7783**
		orange1.1g017142m	AT5G22290.1	NAC domain containing protein 89	1.1842*
miR5037	10.12893993**	**orange1.1g013411m**	**AT2G16980.2**	**Major facilitator superfamily protein**	0.5828**
		**orange1.1g016066m**	**AT2G16990.2**	**Major facilitator superfamily protein**	0.4849**
miR5227	1.8059848**	**orange1.1g031467m**	**AT2G24860.1**	**DnaJ/Hsp40 cysteine-rich domain superfamily protein**	0.4641**
		orange1.1g018585m	AT1G31260.1	Zinc transporter 10 precursor	1.2609**
miR5262	1.64808069**	orange1.1g005832m	AT1G06820.1	Carotenoid isomerase	1.5524**
		**orange1.1g003885m**	**AT5G49890.1**	**Chloride channel C**	0.844**
miR5266	16.22392231**	orange1.1g040022m	AT4G13510.1	Ammonium transporter 1;1	1.2439*
miR5929	−5.83479907**	**orange1.1g005910m**	**AT5G42480.1**	**Chaperone DnaJ-domain superfamily protein**	1.3663**
miR6025	3.39080972**	**orange1.1g005832m**	**AT1G06820.1**	**Carotenoid isomerase**	**0.6716**
		**orange1.1g023118m**	**AT2G21940.4**	**Shikimate kinase 1**	0.7012**
miR6214	−3.978202**	**orange1.1g037661m**	**AT5G37380.4**	**Chaperone DnaJ-domain superfamily protein**	1.2352**
miR6260	−6.8442483**	**orange1.1g010903m**	**AT5G15130.1**	**WRKY DNA-binding protein 72**	3.2313**
		**orange1.1g003752m**	**AT5G42480.1**	**Chaperone DnaJ-domain superfamily protein**	1.3327**
		orange1.1g041599m	AT1G49330.1	Hydroxyproline-rich glycoprotein family protein	0.9023**
		**orange1.1g029026m**	**AT1G64650.1**	**Major facilitator superfamily protein**	1.777**
miR7539	−4.033976**	**orange1.1g002698m**	**AT2G42600.1**	**Phosphoenolpyruvate carboxylase 2**	1.5943**
		**orange1.1g020124m**	**AT2G01060.1**	**MYB-like HTH transcriptional regulator family protein**	1.1878**
miR7841	−10.61512382**	orange1.1g041450m	AT3G42640.1	H^+^-ATPase 8	0.8903**

Both fold change of miRNAs and relative change of target genes are the ratio of B-deficient to –sufficient leaves. The value is an average of at least three biological replicates with three technical replicates; Target genes that had the expected changes in mRNA levels were marked in bold. * and ** indicate a significant difference at *P* < 0.05 and *P* < 0.01, respectively

## Discussion

Evidence shows that miRNAs are involved in the adaptive regulation of higher plants to nutrient deficiencies [8, 13, 17, 19, 24, 27, 37]. Here, we isolated 91 (83 known and 8 novel) up- and 81 (75 known and 6 novel) down-regulated miRNAs from B-deficient leaves (Additional files 3 and 5), indicating that B-deficiency greatly affected the expression profiles of miRNAs in leaves. The differentially expressed miRNAs isolated from leaves were more than from roots [i.e., 52 (40 known and 12 novel) up- and 82 (72 known and 10 novel) down-regulated miRNAs] [8]. The majority of the differentially expressed miRNAs were isolated only from B-deficient roots or leaves, only 22 miRNAs were isolated from the both. Moreover, among the 22 miRNAs, 11 miRNAs in roots and leaves displayed different responses to B-deficiency (Table 3). In conclusion, many differences existed in B-deficiency-induced changes in miRNA expression profiles between roots and leaves.

**Table 3:** List of differentially expressed miRNAs present in both roots and leaves

MiRNA	Fold change	
	Roots	Leaves
miR418	1.87710209**	2.01596507**
miR4413	3.76410603**	−5.94405631**
miR5037	4.79286276**	10.12893993**
miR3946	5.08067752**	−1.66667782**
miR5259	6.34492626**	−5.83479907**
miR2099	13.49283335**	10.31417531**
miR2622	13.96750818**	10.13868134**
miR2664	14.36084091**	−13.05830635**
miR5266	−1.5614939**	16.22392231**
miR394	−5.15694535**	−1.66667782**
miR3513	−5.84396568**	−7.04650639**
miR5492	−6.7798681**	−5.48597088**
miR5534	−7.1665574**	−2.89672418**
miR5029	−7.43642552**	6.19590225**
miR5211	−8.31439018**	14.53849221**
miR1847	−9.0000212**	10.94295432**
miR158	−10.05808647**	−3.35603222**
miR2921	−10.13114959**	−11.0611889**
miR782	−10.76475548**	−10.08402439**
miR1446	−10.94721705**	5.01671689**
miR5074	−10.94721705**	10.74971862**
miR3443	−11.47199392**	9.96792062**

Data from Additional file 3 and Lu et al. [8]; ** indicates a significant difference at *P* < 0.01

We found that *miR159* was down-regulated in B-deficient leaves (Table 2), as previously obtained on salt stressed sugarcane leaves [38]. Patade and Suprasanna showed that the up-regulation of *MYB* at 1 h of salt-stressed sugarcane leaves was accompanied by the down-regulation of *miR159* [38]. However, the expression of *miR159* was up-regulated in P-deficient soybean (*Glycine max*) roots and leaves [39]. MiR159 plays important roles in maintaining leaf phenotype by negatively regulating MYB transcription factors [40]. Dai et al. reported that the expression of *OsMYB3R-2* was induced by various abiotic stresses, and that over-expression of *OsMYB3R-2* enhanced tolerance to freezing, drought, and salt stress in transgenic *Arabidopsis* [41]*.* B-1deficiency affects water uptake into the root, transport through the shoot, and loss of water from the leaves [42]. Thus, B-deficiency-induced down-regulation of *miR159* might increase the expression of *MYB*s (Table 2), thus improving the tolerance of plants to B-deficiency. qRT-PCR showed that all the four *MYBs* target genes (i.e., *MYB domain protein 33*, *MYB domain protein 97*, *MYB-like HTH transcriptional regulator family protein* and *MYB domain protein 65*) were induced by B-deficiency except for the last one. Similarly, the expression levels of *MYB transcription factor (MYBML2)* targeted by miR782, *MYB-like HTH transcriptional regulator family protein* and *MYB domain protein 65* targeted by miR3946, and *MYB-like HTH transcriptional regulator family protein* and *MYB transcription factor (MYBML2)* targeted by miR7539 increased in response to B-deficiency except for *MYB domain protein 65* (Table 2). B-deficiency-induced up-regulation of MYBs in citrus leaves agrees with the previous report that the expression of *MYB85*, *MYB63* and *MYB42* were up-regulated at the slight corking veins and the seriously corky split veins caused by B-deficiency in ‘Newhall’ navel orange (*Citrus sinensis)* leaves [43].

TIR1/AFB2 (TRANSPORT INHIBITOR RESPONSE1/AUXIN SIGNALING F-BOX PROTEIN2) Auxin Receptor (TAAR) family F-box proteins are involved in auxin perception and signaling. The expression of *TAAR* is regulated by miR393 [44]. MiR393 plays a key role in maintaining proper homeostasis of auxin signaling [45]. Si-Ammour et al. showed that miR393 down-regulated all four *TAAR* genes by guiding the cleavage of their mRNAs, leading to the changes in auxin perception and some auxin-related leaf development [44]. Stress-induced increase in *miR393* level may decrease the level of *TIR1*, a positive regulator of growth and development, thereby resulting in attenuation in growth and development during stress conditions [14]. Auxin response factors (ARFs) play a role in relaying auxin signaling at the transcriptional level by inducing mainly three groups of genes [i.e., Aux/IAA (Auxin/indole-3-acetic acid), GH3 and small auxin-up RNA (SAUR)] [46, 47]. MiR160 is predicted to target *ARF10, ARF16* and *ARF17*. MiR160-directed regulation of *Arabidopsis ARF17* is necessary for the normal growth and development of many organs, proper GH3-like gene expression and perhaps auxin distribution, while the *ARF10* and *ARF16* knockout mutants do not display obvious developmental anomalies [48]. Weakened plant growth and reduced metabolic rate are common survival strategies employed to divert energy and other resources to deal with stress conditions. It has been suggested that the stress-induced up-regulation of *miR393* and *miR160* might lead to the attenuation of plant growth and development under stress by repressing auxin signaling due to decreased *TIR1* level and by suppressing the ARF-mediated gene expression, respectively, thus promoting plant stress tolerance [47]. Therefore, B-deficiency-induced up-regulation of leaf *miR393* and *miR160* might be an adaptive response of plants to B-deficiency, because the expression of the three genes targeted by miR160 and *TIR1*, *AFB1, AFB2* and *AFB3* targeted by miR393 was down-regulated by B-deficiency except for *AFB3* (Table 2). Similarly, the expression of *SAUR-like auxin-responsive protein family* targeted by miR3946 was down-regulated in B-deficient leaves despite decreased expression of *miR3946* (Table 2). By contrast, root *miR3946* was up-regulated by B-deficiency [8].

Leaf *miR164* was down-regulated by B-deficiency (Table 2), as previously observed on transient low nitrate-stressed maize leaves [28]. Water stress led to decreased expression of *miR164* in cassava (*Manihot esculenta*) leaves, while its target gene *MesNAC (No Apical Meristem)* was strongly induced [49]. As expected, the expression of *NAC domain transcriptional regulator superfamily protein* and *NAC domain containing protein 100* was induced in B-deficient leaves, while the expression of *NAC domain containing protein 1* was depressed (Table 2). Over-expression of *SNAC1* and *OsNAC6* conferred drought and salt tolerance in rice [50, 51]. *SINAC4*-RNAi tomato plants became less tolerant to salt and drought stress [52]. Therefore, the down-regulation of *miR164* in B-deficient leaves might be involved in the B-deficiency tolerance of plants by improving the expression of *NAC*. However, Xu et al. found that *miR164* was up-regulated in maize leaves under chronic N limitation, and suggested that *miR164* might function in remobilizing the N from old to new leaves to cope with the N-limiting condition *via* accelerating senescence due to decreased expression of NAC [28].

Leaf *miR408* was down-regulated by B-deficiency (Table 2), as previously reported on N-deficient seedlings of *Arabidopsis* [27]. MiR408 targets genes encoding Cu containing proteins such as Cu/Zn SODs (CSDs), plantacyanin and several laccases [23]. Abdel-Ghany and Pilon observed that *miR408* was induced under Cu starvation to down-regulate target gene expression and to save Cu for the most essential functional protein, concluding that might play a role in the regulation of Cu homeostasis [22]. Although B-deficiency decreased leaf concentration of Cu, its level was not lower than the sufficiency range of Cu in citrus leaves [53]. Thus, B-deficiency-induced decrease in *miR408* might be advantageous to plant survival under B-deficiency by regulating Cu homeostasis and improving antioxidant (SOD) activity, because the expression of its four target genes was induced by B-deficiency except for *laccase 12* (Table 2). Indeed, SOD activity was higher in B-deficient *C. sinensis* leaves than in B-sufficient ones [54]. Also, *SOD* expression was up-regulated in B-deficient *Medicago truncatula* root nodules [55].

Leaf *miR477* was up-regulated by B-deficiency (Table 2), as previously reported on salt-stressed *Populus cathayana* plantlets [56]. NAC and GRAS transcription factors are target genes of *miR477. NAC* is involved in developmental process and stress responses [56], while GRAS proteins play a role in signal transduction and the maintenance and development of meristems [57]. Also, *GRAS* is the target gene of miR1446 (Table 2), miR170 and miR171 [58], and *NAC* is the target gene of miR164, miR3953 and miR3946 (Table 2). This indicates the complex regulation in plant development and stress response.

WRKY proteins play important roles in plant responses to (a)biotic stresses, allowing plants to adapt to unfavorable environmental conditions including B-deficiency [59, 60]. Our results showed that leaf transcript of *miR6260* decreased in response to B-deficiency accompanied by increased expression of its target gene: *WRKY DNA-binding protein 72* (Table 2), which agrees with the previous reports that *WRKY3 DNA binding protein* expression was induced in B-deficient *M. truncatula* root nodules [55] and that *WRKY6* was up-regulated in B-deficient *Arabidopsis* roots [60]. Over-expression of various *WRKY* conferred tolerance to different abiotic stresses in different plant species, possible through the regulation of the reactive oxygen species system [61, 62]. Transgenic *Nicotiana benthamiana* plants over-expressing *GhWRKY39* had enhanced tolerance to salt and oxidative stress and increased expression of genes encoding antioxidant enzymes such as SOD, ascorbate peroxidase (APX), catalase (CAT) and glutathione-S-transferase (GST) [62]. Thus, leaf expression levels of antioxidant enzyme genes might be increased in response to B-deficiency. This agrees with our report that B-deficient citrus leaves had higher activities of SOD, APX, MDAR and GR [54]. Heat shock proteins (HSPs)/chaperones function in protecting plants against various stresses. As expected, the expression of *miR6260* was down-regulated in B-deficient leaves accompanied by increased expression of its one target gene: *chaperone DnaJ-domain superfamily protein* (Table 2). Similarly, leaf expression levels of *miR5929* and *miR6214* were decreased by B-deficiency accompanied by increased expression levels of their corresponding target genes: *DnaJ-domain superfamily protein* (AT5G42480.1 and AT5G37380.4; Table 2). However, the expression of *heat shock transcription factor A6B* targeted by miR2099 were inhibited in B-deficient leaves despite down-regulated expression of *miR2099* (Table 2). Hydroxyproline-rich glycoproteins (HRGPs) are the most abundant cell wall structural proteins in dicotyledonous plants [63]. Hall and Cannon demonstrated that the cell wall HRGP RSH was required for normal embryo development in *Arabidopsis* [64]. Bonilla et al. observed that B-deficiency-induced aberrant cell walls of bean root nodules lacked covalently bound HRGPs [65]. Here, the expression of *HRGP family protein* (AT2G25930.1), a target gene of *miR3446*, was up-regulated in B-deficient leaves (Table 2), thus enhancing plant tolerance to B-deficiency. However, *miR3446* was down-regulated in B-deficient leaves, but its target gene (*HRGP family protein*; AT1G49330.1) was also depressed (Table 2).

B-deficiency lowered leaf expression level of *miR158* (Table 2), as previously obtained on N-deficient *Arabidopsis* seedlings [27] and B-deficient citrus roots [8]. The down-regulation of *miR158* means that its target genes: *SPFH/Band 7/PHB domain-containing membrane-associated protein family*, *fucosyltransferase 2* and *lipase class 3 family protein* might be up-regulated in B-deficient leaves. However, qRT-PCR showed that the expression of the former two target genes was induced by B-deficiency, while the last one was down-regulated (Table 2). Lu et al. reported that *fucosyltransferase 2* and *lipase class 3 family protein* were down-regulated in B-deficient citrus roots accompanied by decreased expression of *miR158* [8].

The major facilitator superfamily (MFS) is the largest group of transport carriers, which are often coupled to the movement of another ion [66]. Kaya et al. reported that *ATR1*, which encodes a multidrug resistance transport protein of the MFS, was responsible for most of the tolerance of high B in *Saccharomyces cerevisiae*, concluding that ATR1 was a B exporter [67]. In this study, leaf *miR5037* was induced by B-deficiency accompanied by decreased expression of its target gene: *MFS protein* (Table 2), thus decreasing B export from plants and improving plant tolerance to B-deficiency.

We found that leaf *miR5266* was induced by B-deficiency accompanied by increased expression of its target gene: *ammonium transporter 1;1* (Table 2), which disagrees with our report that the abundance of *miR5266* was lower in B-deficient citrus roots than in controls, while the expression level of *ammonium transporter 1;1* was higher in the former [8].

We observed that *miR3946* was inhibited in B-deficient leaves (Table 2), which disagrees with the previous report that *miR3946* was induced in B-deficient *C. sinensis* roots [8]. All the 17 target genes targeted by miR3946 were induced by B-deficiency except for *homeobox-leucine zipper protein 4 (HB-4)/HD-ZIP protein*, *endosomal targeting BRO1-like domain-containing protein* (AT1G13310.1), *MYB domain protein 65* and *SAUR-like auxin-responsive protein family* (Table 2). Previous studies showed that B-deficiency increased the expression levels of some transport-related genes and the abundances of some transport-related proteins in citrus roots [5, 8], thus improving the tolerance of plants to B-deficiency. *BOR1*, an efflux-type B transporter for xylem loading, play a key role in the tolerance of plants to low B. *Arabidopsis bor1-1* mutant was more sensitive to B-deficiency than the wild type [68]. *Oryza sativa BOR1* has been demonstrated to be required for B acquisition by roots and translocation of B into shoots [69]. Thus, B-deficiency-induced up-regulation of leaf *endosomal targeting BRO1-like domain-containing protein* (AT1G73390.1), *phosphate transporter 1;7*, *MATE efflux family protein*, *vesicle-associated membrane protein 726* (targeted by miR3946), *potassium transport 2/3* (targeted by miR3446), *ammonium transporter 1;1* (targeted by miR5266), *Zn transporter 10 precursor* (targeted by miR5227) and *cation/H*^*+*^*exchanger 25* (targeted by miR2648) involved in cell transport (Table 2) might contribute to the tolerance of citrus to B-deficiency. HD-ZIP transcription factors are found only in plants. The expression of *Hahb-4*, a member of *Helianthus annuus* (sunflower) subfamily I, strongly increased in water-stressed sunflower [70]. Subsequent study showed transgenic *Arabidopsis* plants over-expressing *Hahb-4* were more tolerant to drought by delaying the onset of senescence [71]. Huang et al. demonstrated that *PtrbHLH*, a basic helix-loop-helix transcription factor of *Poncirus trifoliata* might play a crucial role in cold tolerance *via* positively regulating peroxidase (POD)-mediated ROS scavenging [72]. Transketolase is a key enzyme of the pentose phosphate pathway (PPP) in plant cells. Our finding that *transketolase* was up-regulated in B-deficient leaves agrees with the report that transketolase activity in maize moderately increased in response to salt or oxidative stress [73]. In citrus, PPP has been suggested to play a role in the tolerance of plants to B-deficiency by providing reducing power (NADPH) and enhancing the antioxidant capacity [4]. Protein disulfide isomerases (PDIs), which act as molecular chaperones, play a role in the formation of proper disulfide bonds during protein folding [74]. Over-expression of a protein disulfide isomerase-like protein (PDIL) gene conferred Hg tolerance in transgenic plants, which had higher antioxidant capacity and lower levels of superoxide anion radicals, H_2_O_2_ and malondialdehyde (MDA) [75]. As shown in Table 2, the expression level of *PDIL5-3* targeted by miR3946 was increased in B-deficient leaves. To conclude, down-regulation of *miR3946* in B-deficient leaves might be an adaptive response of plants to B-deficiency.

Carotenoid (Car) isomerase (CRTISO), which catalyzes the isomerization of poly-*cis*-carotenoids to all *trans*-carotenoids in higher plants, is a regulatory step for Car biosynthesis. *Arabidopsis* mutants of *crtiso* had increased accumulation of poly-*cis*-carotenoids and reduced lutein concentration [76, 77]. Here, the expression of *miR6025* was increased and its one target gene: *CRTISO* was decreased in B-deficient leaves (Table 2), thus impairing Car biosynthesis. This agrees with our report that B-deficient citrus leaves had lower Car concentration [54]. Plant phenolic secondary metabolites and their precursors are synthesized *via* the pathway of shikimate biosynthesis [78]. Shikimate kinase, a key enzyme for the biosynthesis of polyphenols, catalyzes the fifth reaction of the shikimate pathway. As shown in Table 2, the expression level of *shikimate kinase 1* was down-regulated in B-deficient leaves and the expression of *miR6025*, which targets the gene, was up-regulated. This disagrees with our report that B-deficient citrus leaves displayed increased accumulation of phenolics [4].

Mitogen-activated protein kinase (MAPK) cascades play important roles in plant response to various stresses. Each MAPK cascade consists of MAPKs, MAPK kinases (MAPKKs), and MAPKK kinases (MAPKKKs). In plants, MAPKKKs have been shown to be involved in various stresses. Ning et al. showed that transgenic rice plants over-expressing *DSM1* (a putative MAPKKK gene in rice) displayed higher tolerance to dehydration at the seedling stage by regulating ROS scavenging [79]. In this study, leaf transcript of *miR3446* was decreased by B-deficiency and its target gene *(MAPKKK5*) was up-regulated under B-deficiency. This agrees with the report that *MAPKKK genes* were induced by drought, heat, salt, cold, IAA and jasmonic acid (JA) in *Arabidopsis* [80].

Our finding that leaf expression level of *miR7539* decreased in response to B-deficiency, and its target gene (*phosphoenolpyruvate carboxylase, PEPC*) was induced by B-deficiency (Table 2). This agrees with our report that B-deficient citrus leaves had increased activity of PEPC and dark respiration [4].

## Conclusion

**Fig. 4:**
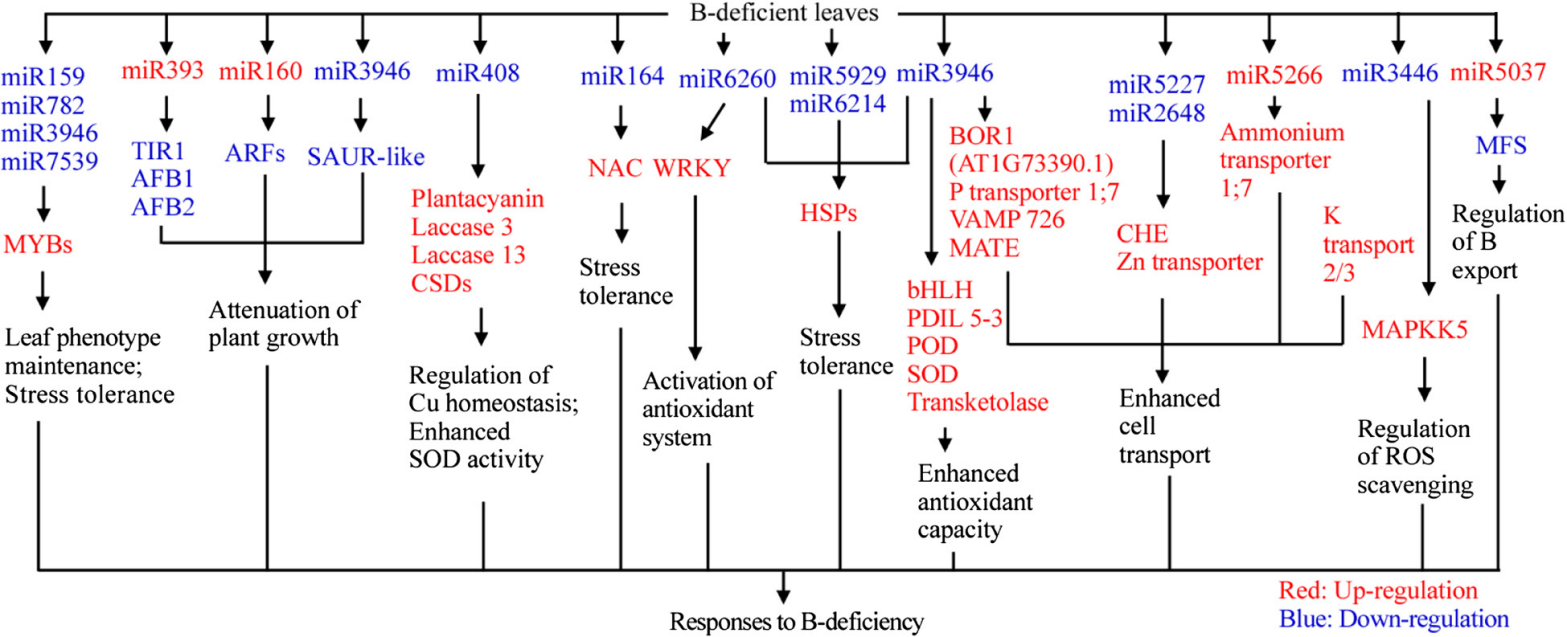
A potential model for the roles of miRNAs in the tolerance of citrus plants to B-deficiency. VAMP 726: vesicle-associated membrane protein 726; CHE: cation/H^+^ exchanger 25

We identified 734 known and 71 novel miRNAs from B-sufficient and -deficient citrus leaves using Illumina sequencing, and obtained 91 (83 known and 8 novel) up- and 81 (75 known and 6 novel) down-regulated miRNAs from B-deficient citrus leaves. Obviously, the expression of miRNAs was greatly altered in B-deficient leaves, which might play a role in the tolerance of plants to B-deficiency. In this study, we proposed a model for the responses of leaf miRNAs to B-deficiency by integrating the present results with the data available in the previous literatures (Fig. 4). The adaptive responses of leaf miRNAs to B-deficiency might be associated with several aspects: (*a*) attenuation of plant growth and development by down-regulating *TIR1*, *ARF* and *AFB* due to up-regulated miR393 and miR160, and by lowering the expression of *SAUR-like auxin-responsive protein family* targeted by miR3946, thus enhancing plant stress tolerance; (*b*) improving the expression of *NACs* due to decreased expression *miR159*, *miR782*, *miR3946* and *miR7539*, hence maintaining leaf phenotype and enhancing the stress tolerance; (*c*) activation of the stress responses and antioxidant system due to decreased expression of *miR164*, *miR6260*, *miR5929*, *miR6214*, *miR3946* and *miR3446*; (*d*) decreased expression of *MFS* resulting from increased expression of *miR5037*, thus lowering B export from plants. In addition, B-deficiency-induced down-regulation of *miR408* might be involved in the tolerance of plants to B-deficiency by regulating Cu homeostasis and enhancing SOD activity. In conclusion, our study reveals some adaptive mechanisms of citrus to B-deficiency.

## Methods

### Plant culture and B treatments

Both plant culture and B treatments were performed according to Yang et al. [5] and Lu et al. [8]. Briefly, 15-week-old seedlings of ‘Xuegan’ [*Citrus sinensis* (L.) Osbeck] grown in 6 L pots (two seedlings per pot) containing fine river sand were supplied every other day until dripping with B-deficient (0 μM H_3_BO_3_) or -sufficient (10 μM H_3_BO_3_) nutrient solution for 15 weeks. There were 10 replications per B treatment with 2 pots in a completely randomized design. At the end of the experiment, fully-expanded leaves from different replicates and treatments were collected at noon under full sun and frozen immediately in liquid N_2_. Leaf samples were stored at −80 °C until extraction. It’s worth mentioning that *C. sinensis* is polyembryonic seed development, an apomictic process in which many embryos are initiated directly from the maternal nucellar cells surrounding the embryo sac containing a developing zygotic embryo [81].

### Isolation of leaf sRNAs, library construction and Illumina sequencing

About 0.1 g mixed frozen B-sufficient and -deficient leaves from five replications were used to extract RNA. Total RNA was extracted from frozen leaves using TRIzol reagent (Invitrogen, Carlsbad, CA) following manufacturer’s instructions. Two sRNA libraries were constructed according to Lu et al. [8]. High throughput sequencing was performed on a Solexa sequencer (Illumina) at the Beijing Genomics Institute (BGI), Shenzhen, China.

### sRNA annotation and miRNA identification

Both sRNA annotation and miRNA identification were performed according to Lu et al. [8]. Briefly, software developed by the BGI was used to deal with the raw data from the Solexa sequencing. Clean reads were then used to analyze length distribution and common/specific sequences. Thereafter, the clear reads were mapped to *C. sinensis* genome (JGIversion 1.1, http://phytozome.jgi.doe.gov/pz/portal.html#!info?alias=Org_Csinensis) using SOAP, only perfectly mapped sequences were retained and analyzed further. rRNAs, tRNAs, snRNAs and snoRNAs were removed from the sRNAs sequences through BLASTn search using NCBI Genebank database (http://www.ncbi.nlm.nih.gov/blast/Blast.cgi/) and Rfam (12.0) database (http://www.sanger.ac.uk/resources/databases/rfam.html) (*e* = 0.01). The remaining sequences were aligned with known plant miRNAs from miRBase 21 (http://www.mirbase.org/). Only the perfectly matched sequences were considered to be conserved miRNAs. Reads that were not annotated were used to predict novel miRNAs using a prediction software Mireap (http://sourceforge.net/projects/mireap/), which was developed by the BGI, by exploring the secondary structure, the Dicer cleavage site and the minimum free energy of the unannotated small RNA tags which could be mapped to genome. In addition, we used MTide: an integrated tool for the identification of miRNA-target interaction in plants (http://bis.zju.edu.cn/MTide) [82] and DNAMAN 8 (http://www.lynnon.com/pc/framepc.html) to predict novel miRNA. Only these miRNA candidates that were simultaneously predicted by the three softwares were considered to be real novel miRNAs.

### Differential expression analysis of miRNAs

Both the fold change between B-deficiency and -sufficiency and the *P*-value were calculated from the normalized expression of TPM [83]. A 1.5 log2-fold cut-off was set to determine up- and down-regulated miRNAs in addition to a *P*-value of less than 0.01 [8].

### Target prediction of miRNAs

This was performed by RNAhybrid based on rules suggested by Allen et al. [84] and Schwab et al. [85].

### Functions of the potential targets of the differentially expressed miRNAs

All targets of the differentially expressed miRNAs were mapped to GO terms in the database (http://www.geneontology.org/), and calculated gene numbers for each term. The GO results were expressed as three categories: cellular component, molecular function, biological process [8].

### Validation of miRNA expression by stem-loop qRT-PCR

The detection of miRNA expression was performed using stem-loop qRT-PCR method, stem-loop primers for reverse transcription and primers for qRT-PCR were listed in Additional file 8. Total RNA was reversetranscribed using Taqman® MicroRNA Reverse Transcription Kit (USA), and SYBR® Premix Ex Taq™ II (Takara, Japan) kit was used for qRT-PCR. MiRNA special (forward) primers were designed according to the miRNA sequence but excluded the last six nucleotides at 3’ end of the miRNA. A 5’ extension of several nucleotides, which was chosen randomly and relatively GC-rich, was added to each forward primer to increase the melting temperature [86]. All the primers were assigned to Primer Software Version 5.0 (PREMIER Biosoft International, USA) to assess their quality. For qRT-PCR, 20 μL reaction solution contained 10 μL ready-to-use SYBR® Premix Ex TaqTM II (Takara, Japan), 0.8 μL 10 μM miRNA forward primer, 0.8 μL 10 μM Uni-miR qPCR primer, 2 μL cDNA template and 6.4 μL dH_2_O. The cycling conditions were 60 s at 95 °C, followed by 40 cycles of 95 °C for 10 s, 60 °C for 30 s. qRT-PCR was performed on the ABI 7500 Real Time System. Samples for qRT-PCR were run in at least three biological replicates with two technical replicates. Relative miRNA expression was calculated using ddCt algorithm. For the normalization of miRNA expression, *actin* (AEK97331.1) was used as an internal standard and the leaves from control plants were used as reference sample, which was set to 1.

### qRT-PCR analysis of miRNA target gene expression

Total RNA was extracted from frozen B-sufficient and -deficient leaves using TRIzol reagent (Invitrogen, Carlsbad, CA) following manufacturer’s instructions. The sequences of the F and R primers used were given in Additional file 9. qRT-PCR analysis of miRNA target gene expression was performed using a ABI 7500 Real Time System according to Lu et al. [8].

### Experimental design and statistical analysis

There were 20 pot seedlings per treatment in a completely randomized design. Experiments were performed with 3 replicates. Differences among treatments were separated by the least significant difference (LSD) test at *P* < 0.05 level.

### Availability of data and materials

“The data set supporting the results of this article are available in the Gene Expression Omnibus repository under accession no GSE72108 (http://www.ncbi.nlm.nih.gov/geo/query/acc.cgi?acc=GSE72108)”. The mature miRNA and precursor sequences will be submitted to miRBase registry and assigned final names after final acceptance of the manuscript.

## Additional files

Additional file 1:
**Length distribution of small RNAs from control and B-deficient leaves of** ***Citrus sinensis*** **seedlings.** (DOC 81 kb) Additional file 2:
**List of known miRNAs in** ***Citrus sinensis*** **leaves.** (DOC 1525 kb) Additional file 3:
**List of known miRNAs in** ***Citrus sinensis*** **leaves after removing these miRNAs with normalized read-count less than 10 TPM in the two miRNA libraries constructed from control and B-deficient leaves.** (DOC 452 kb) Additional file 4:
**List of novel miRNAs in** ***Citrus sinensis*** **leaves.** (DOC 158 kb) Additional file 5:
**List of novel miRNAs in** ***Citrus sinensis*** **leaves after removing these miRNAs with normalized read-count less than 10 TPM in two miRNA libraries constructed from control and B-deficient leaves.** (DOC 69 kb) Additional file 6:
**List of target genes for parts of known miRNAs in** ***Citrus sinensis*** **leaves.** (DOC 198 kb) Additional file 7:
**List of target genes for parts of novel miRNAs in** ***Citrus sinensis*** **leaves.** (DOC 33 kb) Additional file 8:
**List of stem loop qRT-PCR primers.** (DOC 61 kb) Additional file 9:
**Specific primer pairs used for qRT-PCR expression analysis of selected miRNA target genes.** (DOC 160 kb)
