# Supplementary material for: Boron-deficiency-responsive microRNAs and their targets in Citrus sinensis leaves
Source: BMC Plant Biol. 2015 Nov 4;15:271. doi: 10.1186/s12870-015-0642-y (PMC4634795; doi:10.1186/s12870-015-0642-y)
Supplement: Additional file 1: — Length distribution of small RNAs from control and B-deficient leaves of Citrus sinensis seedlings. (DOC 81 kb) [file 12870_2015_642_MOESM1_ESM.doc]

**Additional file 1: Length distribution of small RNAs from control and B-deficient leaves of *Citrus sinensis* seedlings.**
