# Supplementary material for: Boron-deficiency-responsive microRNAs and their targets in Citrus sinensis leaves
Source: BMC Plant Biol. 2015 Nov 4;15:271. doi: 10.1186/s12870-015-0642-y (PMC4634795; doi:10.1186/s12870-015-0642-y)
Supplement: Additional file 2: — List of known miRNAs in Citrus sinensis leaves. (DOC 1525 kb) [file 12870_2015_642_MOESM2_ESM.doc]

**Additional file 2: List of known miRNAs in *Citrus sinensis* leaves**.

| miRNA | *Sequence* | Expressed | | Normalized read count | | Fold change |
| --- | --- | --- | --- | --- | --- | --- |
|  |  | B-deficiency | Control | B-deficiency | Control |  |
| ***Up-regulated miRNAs*** | |  |  |  |  |  |
| miR5266 | CGGGGGACGGUCUGGGAACG | 13647 | 0 | 765.3969 | 0.01 | 16.22392231** |
| miR5760 | UGUUUAAGAUAGUUUGUAAGGAU | 4447 | 0 | 249.4116 | 0.01 | 14.60623973** |
| miR5211 | UCGCAGGGGAGAUGGGACCGC | 4243 | 0 | 237.9702 | 0.01 | 14.53849221** |
| miR1151 | ACGGGGUUGUGGGAGAGCGGAC | 3306 | 0 | 185.4182 | 0.01 | 14.17849626** |
| miR7693 | GACGCAUCGAUGAAGAACGU | 3046 | 0 | 170.836 | 0.01 | 14.0603237** |
| miR5666 | AGGGACAUAGAGACAUUUACU | 2818 | 0 | 158.0485 | 0.01 | 13.94808012** |
| miR859 | UGAUUUUAGAAUAAGAGAUGA | 2790 | 0 | 156.4781 | 0.01 | 13.93367328** |
| miR2928 | AAGAAGAAGAAGUUUGUU | 2187 | 0 | 122.6587 | 0.01 | 13.58236255** |
| miR7721 | CGGUUUUAUAACGGUCUUUAGG | 1306 | 0 | 73.2475 | 0.01 | 12.83856413** |
| miR8039 | UUUCCUACUGAGAUUAUCAAC | 873 | 0 | 48.9625 | 0.01 | 12.25746177** |
| miR3510 | UUUAUACAUGCUCUGCAGACUGA | 843 | 0 | 47.28 | 0.01 | 12.20701447** |
| miR5076 | GAAAUGUGAGUAGAGCAGGUAUU | 816 | 0 | 45.7657 | 0.01 | 12.16005071** |
| miR7727 | UCAAGAUCGAUUGGAAUAAGGGC | 648 | 0 | 36.3433 | 0.01 | 11.8274736** |
| miR1159 | AACAAUGCCUGUGGAGACGAU | 628 | 0 | 35.2216 | 0.01 | 11.78224467** |
| miR5057 | CAAUUUGCAGAUCUUUUGACA | 455 | 0 | 25.5188 | 0.01 | 11.31734461** |
| miR5812 | UCAAGACUUUUAAGAUUGUGGCC | 454 | 0 | 25.4628 | 0.01 | 11.31417531** |
| miR5295 | UGGGUUGGGAAUGAAAAUGAGGG | 446 | 0 | 25.0141 | 0.01 | 11.28852593** |
| miR7127 | AUUCUCAUCGAUUUGGAUA | 369 | 0 | 20.6955 | 0.01 | 11.01510148** |
| miR6229 | AUAUCUCACUUGAGCGUCGGAGG | 367 | 0 | 20.5833 | 0.01 | 11.00725872** |
| miR6143 | AAGACUGUAGUGAACAUG | 6222 | 3 | 348.9631 | 0.1705 | 10.99908716** |
| miR1847 | UUCUAAUUUGAGUUGUGGCAC | 351 | 0 | 19.686 | 0.01 | 10.94295432** |
| miR1162 | CGGCUUAAUUUGACACAACACG | 311 | 0 | 17.4425 | 0.01 | 10.76839115** |
| miR5074 | GCAAGGCCACCGUGCCGGCGACGC | 307 | 0 | 17.2182 | 0.01 | 10.74971862** |
| miR7726 | UGUUGCAUGUCGGACGUCACGGU | 305 | 0 | 17.106 | 0.01 | 10.74028674** |
| miR5525 | UCAAUCCUUGUGGAGACGAUCUGA | 302 | 0 | 16.9378 | 0.01 | 10.7260309** |
| miR7489 | AUUGUGCAAUACAAGAAGAUCGU | 258 | 0 | 14.47 | 0.01 | 10.49884921** |
| miR1024 | UCUGGUUCUGAUUGUAUGGUCUC | 232 | 0 | 13.0118 | 0.01 | 10.3456048** |
| miR2099 | AAAGGCUGUACGUUAUUU | 227 | 0 | 12.7314 | 0.01 | 10.31417531** |
| miR860 | UCAAUUAGAUUGGAUAUAUGGAUA | 226 | 0 | 12.6753 | 0.01 | 10.30780411** |
| miR7780 | GUAUGGGACCUUGGCUAUAGACGUU | 202 | 0 | 11.3292 | 0.01 | 10.14583021** |
| miR2622 | UUUGUGGCCACCGUGAACUAA | 201 | 0 | 11.2732 | 0.01 | 10.13868134** |
| miR5037 | AUGGAACUUUGAAGGCCG | 3404 | 3 | 190.9146 | 0.1705 | 10.12893993** |
| miR5651 | UUGUGCGGAUCAAAUUGUUAGAC | 198 | 0 | 11.1049 | 0.01 | 10.11698066** |
| miR8145 | AAAACAGAUAGAAUGCCACUU | 196 | 0 | 10.9927 | 0.01 | 10.10233009** |
| miR7525 | AGAGGUUUGGGUACAUACU | 194 | 0 | 10.8806 | 0.01 | 10.08754234** |
| miR5566 | UAAGCAUACACCUCCCUCUUU | 189 | 0 | 10.6001 | 0.01 | 10.0498622** |
| miR5233 | GAGUAGGAUGGUCGUUGGA | 187 | 0 | 10.488 | 0.01 | 10.03452393** |
| miR3443 | GUGCGUUUGAGAUUGAGGUU | 5073 | 5 | 284.521 | 0.2841 | 9.96792062** |
| miR826 | UUGCUGGUUUUGGAUGACGUG | 173 | 0 | 9.7028 | 0.01 | 9.92225737** |
| miR7745 | UGAAAGGUUUAGAACUAAGAGAU | 168 | 0 | 9.4223 | 0.01 | 9.87993545** |
| miR5294 | GCUAGAAUCGGAAUGAGUUAGCAU | 165 | 0 | 9.2541 | 0.01 | 9.85394889** |
| miR3638 | GAACAAGCACAAAAGAGGACACC | 2771 | 3 | 155.4125 | 0.1705 | 9.83211503** |
| miR842 | UCUGCUCUAGAUCCGUCAUC | 152 | 0 | 8.525 | 0.01 | 9.73555606** |
| miR5638 | AUACCAAGUACUCUCACACUU | 149 | 0 | 8.3567 | 0.01 | 9.70678955** |
| miR6425 | CUUGCUUCCAUGGACGUAGGCAUG | 145 | 0 | 8.1324 | 0.01 | 9.66753737** |
| miR8019 | AAGAAUGACAUCGUUUGACAUUG | 145 | 0 | 8.1324 | 0.01 | 9.66753737** |
| miR6421 | AGAGAGAUUGUUGGGGAAG | 141 | 0 | 7.908 | 0.01 | 9.6271691** |
| miR7740 | UUUGUAGAUCAACCUCGGACUCAU | 141 | 0 | 7.908 | 0.01 | 9.6271691** |
| miR6145 | CACUGUUCACAUGUACGACACUAC | 123 | 0 | 6.8985 | 0.01 | 9.43013888** |
| miR5206 | AGUGGUGAUCGUGUUGGUGGGUUU | 122 | 0 | 6.8424 | 0.01 | 9.41835864** |
| miR1533 | AUAAUAAACAACAGUAGAUGA | 117 | 0 | 6.562 | 0.01 | 9.35799181** |
| miR6020 | AAAGUUUUUCUAGAAAUCUUC | 109 | 0 | 6.1133 | 0.01 | 9.25580758** |
| miR7785 | GUGAAUGGGUAGAGAGAGAAGAC | 615 | 1 | 34.4925 | 0.0568 | 9.24617605** |
| miR533 | GAGUGGCCAGGGCUGUUGGAGGGC | 107 | 0 | 6.0011 | 0.01 | 9.22908313** |
| miR5998 | ACAUUUAUGUGUUUGCUUUGU | 106 | 0 | 5.945 | 0.01 | 9.21553299** |
| miR1103 | UGGAAAAUGGAUGGUGCAGUUUU | 105 | 0 | 5.889 | 0.01 | 9.20187884** |
| miR5222 | CUAAGGAGAAGAAUGAGGC | 105 | 0 | 5.889 | 0.01 | 9.20187884** |
| miR8043 | UGAUGAUAAUUGGACAUUGCA | 105 | 0 | 5.889 | 0.01 | 9.20187884** |
| miR951 | GUUCUUGAGUUGGACCAA | 103 | 0 | 5.7768 | 0.01 | 9.17412672** |
| miR1870 | GAAGAAUUAGAACCUAGUGGCAU | 99 | 0 | 5.5525 | 0.01 | 9.11699371** |
| miR3981 | CUAUUAGGAUGCGUCUCAU | 97 | 0 | 5.4403 | 0.01 | 9.08754242** |
| miR6103 | CAAAAGUUGAUCAGGCAUG | 93 | 0 | 5.2159 | 0.01 | 9.02677238** |
| miR5026 | CCAAUAAGAUCGUGACACU | 90 | 0 | 5.0477 | 0.01 | 8.97948236** |
| miR7517 | AUAUGCUAAGGUUACGGGAUCC | 86 | 0 | 4.8233 | 0.01 | 8.91387676** |
| miR5742 | CAACAAUCAAUGGUAGUUGGA | 79 | 0 | 4.4307 | 0.01 | 8.79139084** |
| miR6213 | UCAGAUUGCUAAGAUGGU | 78 | 0 | 4.3747 | 0.01 | 8.7730403** |
| miR8152 | UUGCACAGUAGGUGGACCCAU | 65 | 0 | 3.6455 | 0.01 | 8.50997291** |
| miR5486 | AGGGAUGCAUAUUCUACUCA | 64 | 0 | 3.5895 | 0.01 | 8.4876391** |
| miR3710 | UGGGAAUCCUGACGGCCUA | 63 | 0 | 3.5334 | 0.01 | 8.46491326** |
| miR1881 | AAUGUUAUUGGUACGUGGCGUUGU | 60 | 0 | 3.3651 | 0.01 | 8.39450556** |
| miR5215 | AGAUGAGGAUGAGCUAAGUGCUU | 60 | 0 | 3.3651 | 0.01 | 8.39450556** |
| miR3635 | GCAAGUGUUGGGCAUAAUCAG | 58 | 0 | 3.253 | 0.01 | 8.34562699** |
| miR417 | GAAGAAUGAUUUUGUUCGA | 57 | 0 | 3.1969 | 0.01 | 8.3205298** |
| miR5797 | CGUGGGAUGUAUGCAGAA | 56 | 0 | 3.1408 | 0.01 | 8.29498826** |
| miR5229 | UGUAGCAGGAAUAGUGAACUAU | 55 | 0 | 3.0847 | 0.01 | 8.26898639** |
| miR5777 | CUGGAGAAUAAUGUUGGAUGACGAC | 55 | 0 | 3.0847 | 0.01 | 8.26898639** |
| miR5763 | UGAAUAUGACUGAAAGACGCGUUA | 53 | 0 | 2.9725 | 0.01 | 8.21553299** |
| miR5284 | AGGGACCUAAGUGGAGAAUCCU | 52 | 0 | 2.9164 | 0.01 | 8.18804481** |
| miR1427 | ACGGAGCCGUGCGGUGGGC | 51 | 0 | 2.8604 | 0.01 | 8.16007312** |
| miR1087 | UGCAGAUUGAGAGUUAGGCUU | 49 | 0 | 2.7482 | 0.01 | 8.10234317** |
| miR4383 | UAUUGAAUUCUCAGUUGGACGGU | 44 | 0 | 2.4678 | 0.01 | 7.94708168** |
| miR5578 | UUAUGUGGAUGAUGGCAAGAAGGU | 41 | 0 | 2.2995 | 0.01 | 7.84517638** |
| miR1428 | UAAAUAAUGCAUCGAAUUCU | 40 | 0 | 2.2434 | 0.01 | 7.80954308** |
| miR5061 | UCUGAUCUCGCUCCUGCUCGGCA | 40 | 0 | 2.2434 | 0.01 | 7.80954308** |
| miR4379 | UGAAUGCUAUUACUGUGAGAGGCC | 39 | 0 | 2.1873 | 0.01 | 7.77300729** |
| miR6423 | UGCUGUCGCCACUAUCUUCGCA | 38 | 0 | 2.1312 | 0.01 | 7.73552217** |
| miR5151 | UAAUGAUGUGGUACUAAUGA | 37 | 0 | 2.0752 | 0.01 | 7.69710659** |
| miR7700 | UGAAGUUUGGGACAGCUUGCA | 37 | 0 | 2.0752 | 0.01 | 7.69710659** |
| miR2112 | CGAAAUGCGGAAUGAAGGU | 36 | 0 | 2.0191 | 0.01 | 7.65756855** |
| miR5285 | UAGGACUUAGGGCUAAAUUAGGC | 36 | 0 | 2.0191 | 0.01 | 7.65756855** |
| miR3699 | ACAGAAGAUAGACAUUUGAAC | 782 | 4 | 43.8587 | 0.2273 | 7.59212154** |
| miR812 | ACGGCACGAUUAAAGAUGGGCCAU | 1154 | 6 | 64.7225 | 0.341 | 7.56835179** |
| miR5235 | AUAGGUCAAUGUUGGUGUU | 33 | 0 | 1.8508 | 0.01 | 7.53200521** |
| miR2671 | UUAAAAAUAUUCGUUCGGUCC | 31 | 0 | 1.7386 | 0.01 | 7.44178224** |
| miR2875 | AAUUAUCAGUCAUAUACUUUUAA | 31 | 0 | 1.7386 | 0.01 | 7.44178224** |
| miR4408 | UAAGGACAUUGGUGAAGGGUUGA | 31 | 0 | 1.7386 | 0.01 | 7.44178224** |
| miR6021 | UUGGAGAUGCUUGCUAUUGAA | 30 | 0 | 1.6826 | 0.01 | 7.39454843** |
| miR6141 | ACAACUCUGGCCUUGUAGCGGA | 30 | 0 | 1.6826 | 0.01 | 7.39454843** |
| miR7787 | GCUUUAGGUCGUGUGCUUGGGCAC | 30 | 0 | 1.6826 | 0.01 | 7.39454843** |
| miR821 | AAGACACUCAACAAAAAGUUGCAU | 30 | 0 | 1.6826 | 0.01 | 7.39454843** |
| miR2613 | AGGUUCGCCGUGGUCAACGGU | 28 | 0 | 1.5704 | 0.01 | 7.29498826** |
| miR7824 | UUGAAAAAGCUGAAUCGGAUC | 28 | 0 | 1.5704 | 0.01 | 7.29498826** |
| miR8048 | AGAGUGGACAGCUAAUGCA | 28 | 0 | 1.5704 | 0.01 | 7.29498826** |
| miR5828 | UUAAGGGCAUUAAAAUUAAAU | 27 | 0 | 1.5143 | 0.01 | 7.24250723** |
| miR902 | AUGAAGGUCGUGUAAUCGUGC | 149 | 1 | 8.3567 | 0.0568 | 7.2008986** |
| miR4232 | UCAAUUUAUUUAGCGAUGUGGUAC | 26 | 0 | 1.4582 | 0.01 | 7.18804479** |
| miR1223 | UUCUAAGUCAUACAGCUUCU | 25 | 0 | 1.4021 | 0.01 | 7.13144544** |
| miR5758 | UAAAUUGGAAGAAGUCAUUGG | 24 | 0 | 1.346 | 0.01 | 7.0725346** |
| miR5512 | UGGAUAUGAGUAAUGAUAGAA | 23 | 0 | 1.29 | 0.01 | 7.01122725** |
| miR5544 | AUGAAACGUAGGUAGAAGUUGGAU | 23 | 0 | 1.29 | 0.01 | 7.01122725** |
| miR7823 | UUCAUGGAAGAAGCUUGAAAU | 23 | 0 | 1.29 | 0.01 | 7.01122725** |
| miR5033 | UGGCUGUACAAAAGUAAUCUC | 22 | 0 | 1.2339 | 0.01 | 6.94708166** |
| miR5165 | CUCUAGGGCUCUAAGUGUAGGUU | 22 | 0 | 1.2339 | 0.01 | 6.94708166** |
| miR4363 | CAUUACAGAAGGCUUAGAG | 21 | 0 | 1.1778 | 0.01 | 6.87995078** |
| miR4368 | AAGACGGGACUUACUAUCAGUAAA | 21 | 0 | 1.1778 | 0.01 | 6.87995078** |
| miR6161 | AAUAUAAGUGGAGUUGGUGUCCU | 21 | 0 | 1.1778 | 0.01 | 6.87995078** |
| miR6439 | AGAAAGAAGAAGAGAAGCC | 119 | 1 | 6.6742 | 0.0568 | 6.87656017** |
| miR5634 | AGGAGACUUUGCUGAAUUUUUGG | 20 | 0 | 1.1217 | 0.01 | 6.80954306** |
| miR5767 | UAGAGGACACUUUGAAGGGCA | 20 | 0 | 1.1217 | 0.01 | 6.80954306** |
| miR7505 | UACAGACAACCAUUCUUCCUU | 20 | 0 | 1.1217 | 0.01 | 6.80954306** |
| miR161 | GCAUUCAUUGAAGUGACUA | 19 | 0 | 1.0656 | 0.01 | 6.73552217** |
| miR5248 | UUUUUAGGUUGAGCAUGACAUACA | 19 | 0 | 1.0656 | 0.01 | 6.73552217** |
| miR6107 | AAAGUGGACAAUAUCUUGCUAGG | 18 | 0 | 1.0095 | 0.01 | 6.6574971** |
| miR6221 | UUUCUGACUUUGGCGCCCGUGCAU | 18 | 0 | 1.0095 | 0.01 | 6.6574971** |
| miR5508 | UAUGAUGGCUGUUGGUGGUGG | 86 | 1 | 4.8233 | 0.0568 | 6.40798581** |
| miR5024 | AUAAGAACGGCCAAGAUACUAACA | 150 | 2 | 8.4128 | 0.1137 | 6.20928189** |
| miR5029 | AUGAAAGACGAACAACUGCAAA | 4531 | 61 | 254.1228 | 3.4665 | 6.19590225** |
| miR6136 | UCAUUCCACACGUCGUCUAUA | 813 | 11 | 45.5974 | 0.6251 | 6.18872075** |
| miR840 | AGACUGAAGGACCUAAAAUCAAGC | 64 | 1 | 3.5895 | 0.0568 | 5.98174816** |
| miR5750 | AAAGAGAUAGUCAGAUUGCAU | 441 | 7 | 24.7336 | 0.3978 | 5.95828516** |
| miR5761 | UGUUUGAGUCGUUAGCUUUUG | 370 | 7 | 20.7516 | 0.3978 | 5.70503549** |
| miR5766 | UUGAGGCAUGUAGAAGAGCAA | 5816 | 122 | 326.1924 | 6.933 | 5.5560996** |
| miR5561 | CAUAAGAGAGAGAGCAUAGACAA | 173 | 4 | 9.7028 | 0.2273 | 5.41573154** |
| miR5665 | UUGGUGGAUUAGAUUGGGUAU | 41 | 1 | 2.2995 | 0.0568 | 5.33928546** |
| miR6117 | GGUUUUAGGUUGUUCGGGUUAGAC | 41 | 1 | 2.2995 | 0.0568 | 5.33928546** |
| miR5776 | ACUAUGGGCUGACCUUAGGUGG | 541 | 14 | 30.3422 | 0.7956 | 5.2531386** |
| miR1120 | ACUUUUAUUUAUGGGACGGGAGG | 66 | 2 | 3.7016 | 0.1137 | 5.02484485** |
| miR1446 | CGAACUCUCUCCCUCAACGGC | 8594 | 262 | 481.9976 | 14.8889 | 5.01671689** |
| miR3631 | AUAUUGGUUGAUCUCAUACAA | 32 | 1 | 1.7947 | 0.0568 | 4.98170797** |
| miR5801 | CCAAAUUGUUUUCGUCUUGGAU | 32 | 1 | 1.7947 | 0.0568 | 4.98170797** |
| miR6262 | UCUUUAGACAACGGUAGAAUGGU | 11670 | 392 | 654.5161 | 22.2765 | 4.8768342** |
| miR5270 | GAGAGAGAAGAGUUUUAGGGAUU | 29 | 1 | 1.6265 | 0.0568 | 4.83973608** |
| miR6430 | UGAUGAUAAUUGGACAUUGCACAA | 29 | 1 | 1.6265 | 0.0568 | 4.83973608** |
| miR8144 | AAGUAGAAGAGGUAGAAACGGA | 226 | 8 | 12.6753 | 0.4546 | 4.8012784** |
| miR7707 | UUUAGAUGAUGUAUGGAACGG | 607 | 22 | 34.0438 | 1.2502 | 4.76716127** |
| miR4346 | UAAACCCAACGAGAGAGCUGCAU | 27 | 1 | 1.5143 | 0.0568 | 4.73661631** |
| miR3509 | AUCUAACGACUGCUACAUAAUCAU | 162 | 6 | 9.0858 | 0.341 | 4.7357699** |
| miR8141 | UGGAUACAGUUAGAUUGGUU | 367 | 14 | 20.5833 | 0.7956 | 4.69328721** |
| miR7822 | UUCUGAAAUUGAAAAAGGCGUA | 712 | 28 | 39.9328 | 1.5912 | 4.64938715** |
| miR1142 | AAGGUGUGGAAUGGGCAUG | 151 | 6 | 8.4689 | 0.341 | 4.63433095** |
| miR5494 | UUAGCUUGGAGGUAUAGACGUA | 944 | 39 | 52.9446 | 2.2163 | 4.57825846** |
| miR5232 | UACAUUCCUCUCACAUGAU | 120 | 5 | 6.7302 | 0.2841 | 4.56617864** |
| miR7540 | UUAUAGAUGAUAAGUGAUGUAGA | 47 | 2 | 2.636 | 0.1137 | 4.53504621** |
| miR6151 | CUGAUAUGUAGAGGAUUGGAUUGU | 67 | 3 | 3.7577 | 0.1705 | 4.46200625** |
| miR2083 | GAGAUUGAGAGCUGCAGAAGA | 111 | 5 | 6.2255 | 0.2841 | 4.45371897** |
| miR2874 | AUGUGAACAGUGUCGUGAACAGUU | 193 | 9 | 10.8245 | 0.5115 | 4.40342234** |
| miR6288 | AACCGAAUUAGAAAAAACCGUCGG | 83 | 4 | 4.6551 | 0.2273 | 4.35614257** |
| miR7711 | UGAUGGAAUUGUAAAUUAGAAGA | 81 | 4 | 4.5429 | 0.2273 | 4.32094396** |
| miR2619 | AGCAUAGGAGGGCGUGUUCUUGAU | 19 | 1 | 1.0656 | 0.0568 | 4.22963125** |
| miR5498 | UGGACAGCUGUAAUCAUUGAAGAC | 270 | 16 | 15.143 | 0.9092 | 4.05790955** |
| miR5340 | UUGGAUGACAUGAUUAAUUUCAAA | 31 | 2 | 1.7386 | 0.1137 | 3.93462189** |
| miR6422 | UAUGAAAUGAAGGGAUAUGGU | 161 | 11 | 9.0297 | 0.6251 | 3.85251915** |
| miR477 | ACCUCCCUCGAAGGCUUCCAA | 1433 | 100 | 80.3703 | 5.6828 | 3.82198862** |
| miR3953 | UUGAGUUCUGCAAGCCGUCGA | 311 | 22 | 17.4425 | 1.2502 | 3.80237602** |
| miR7728 | UUUGGGAUUGAGGUAUUUU | 126 | 9 | 7.0668 | 0.5115 | 3.78825093** |
| miR5716 | UUGAUAAUUGAGAGAGUAAUGAAA | 55 | 4 | 3.0847 | 0.2273 | 3.7624606** |
| miR5304 | UGUGAGUAUGGGCAUUGA | 40 | 3 | 2.2434 | 0.1705 | 3.71784323** |
| miR8000 | AACCGAACCGAACCGAACCGAAGA | 524 | 42 | 29.3887 | 2.3868 | 3.62211196** |
| miR5218 | GUGAGACUUAGAGUGUAAGAUGAC | 61 | 5 | 3.4212 | 0.2841 | 3.59003171** |
| miR5640 | UGAGAAGCAGGAAUUAGAAUA | 72 | 6 | 4.0381 | 0.341 | 3.56583299** |
| miR6190 | CAGGAAAGGAACAAGCAUU | 57 | 5 | 3.1969 | 0.2841 | 3.49220288** |
| miR1048 | UAGAACUCAUGAGUGUAGAAGAA | 250 | 22 | 14.0213 | 1.2502 | 3.4873893** |
| miR6253 | GAGAGAAAGUGGGCUGGGUU | 130 | 12 | 7.2911 | 0.6819 | 3.4185044** |
| miR848 | UGACAUGGUACAACAGAUUA | 32 | 3 | 1.7947 | 0.1705 | 3.39589906** |
| miR6025 | UACCAACAAGAGAUGAACAUU | 457 | 43 | 25.631 | 2.4436 | 3.39080972** |
| miR856 | UAAAUCCUACCCAUAUACUAUCAC | 21 | 2 | 1.1778 | 0.1137 | 3.37279042** |
| miR5788 | UGGAUGUAGAGCAUACUCAGUAUA | 51 | 5 | 2.8604 | 0.2841 | 3.33174617** |
| miR7747 | UCAGUUUGUUUUGUGUAGGGAUGG | 29 | 3 | 1.6265 | 0.1705 | 3.25392718** |
| miR6281 | UGGGUAGAGAGAGAGAGAGUGAG | 679 | 72 | 38.082 | 4.0916 | 3.21837223** |
| miR779 | UCUGCUCAUGGAUUGUCUGCUCAU | 1365 | 146 | 76.5565 | 8.2969 | 3.20588057** |
| miR1879 | GUUUGUUUGGUUUGGGAGAGAUGG | 110 | 12 | 6.1694 | 0.6819 | 3.1774981** |
| miR319 | AUCCAACGAAGCAGGAGCUGU | 169 | 19 | 9.4784 | 1.0797 | 3.13401304** |
| miR5668 | AGCAAUGGAAUCAUGACGC | 53 | 6 | 2.9725 | 0.341 | 3.12383317** |
| miR1534 | UAUUUAGGGGUAAAAUUGUCAU | 44 | 5 | 2.4678 | 0.2841 | 3.11875474** |
| miR1510 | AGGGAUAGGUAAAACAACGAC | 78 | 9 | 4.3747 | 0.5115 | 3.09637794** |
| miR5205 | AUGACAAUUUGGGACGGGAUUGAG | 26 | 3 | 1.4582 | 0.1705 | 3.09634496** |
| miR5251 | AUUAGAUCUAGGGUUUCUU | 120 | 14 | 6.7302 | 0.7956 | 3.0805342** |
| miR5296 | AAUUUUGUGGGUUGUAAGCAGGUU | 25 | 3 | 1.4021 | 0.1705 | 3.0397456** |
| miR2628 | AAUGAAAGAGAUGAUGAUAU | 65 | 8 | 3.6455 | 0.4546 | 3.00344711** |
| miR6287 | CUGAACACUAGUGGAAGUUUUGGGC | 32 | 4 | 1.7947 | 0.2273 | 2.98107312** |
| miR5672 | CUGGAAUGGAAGCAAAUGGA | 63 | 8 | 3.5334 | 0.4546 | 2.95838749** |
| miR1439 | UUUAGGAACGGGAGUGAGAUAUU | 30 | 4 | 1.6826 | 0.2273 | 2.88802266** |
| miR7507 | AAGGUGGGAGUAGGCAAUAAUGGG | 22 | 3 | 1.2339 | 0.1705 | 2.85538183** |
| miR2630 | UUGUUUUGGCCUUCGGUUUU | 1369 | 197 | 76.7809 | 11.1951 | 2.77788006** |
| miR4366 | UACUGUAGUAGUGUUUGUUGG | 400 | 60 | 22.4341 | 3.4097 | 2.71797659** |
| miR8136 | UGAAAGCAUGGAAUUGGGAGUAAA | 53 | 8 | 2.9725 | 0.4546 | 2.70900722** |
| miR5824 | AGUCGAGUAAGAAGUGAAGGCAU | 358 | 55 | 20.0786 | 3.1255 | 2.68349977** |
| miR3949 | UGAUGUUAGUGCAAAAGUGGUUAG | 26 | 4 | 1.4582 | 0.2273 | 2.68151902** |
| miR952 | AAACAGAACAUGGCAUUGGUCC | 779 | 121 | 43.6905 | 6.8762 | 2.6676362** |
| miR415 | AACAGAAGGAGAAGGACGAGCAG | 193 | 30 | 10.8245 | 1.7048 | 2.66662598** |
| miR6218 | ACAAGUUCGUGUCUUUGGG | 57 | 9 | 3.1969 | 0.5115 | 2.64386747** |
| miR2922 | AAUAAGUAGAUUCGAAAUA | 68 | 11 | 3.8138 | 0.6251 | 2.60907028** |
| miR5540 | UUGUGCGCGCUCGACCGGUAU | 30 | 5 | 1.6826 | 0.2841 | 2.56622151** |
| miR1216 | UGUAGUGAUGGCGGUUGUAUC | 18 | 3 | 1.0095 | 0.1705 | 2.56579727** |
| miR479 | UGUGAUAUUGGUUCGGCUCAUC | 17994 | 3185 | 1009.1999 | 180.9967 | 2.47917667** |
| miR8127 | CAACUGUGGAAGACACCUUUA | 4149 | 750 | 232.6981 | 42.6209 | 2.44882647** |
| miR5241 | UACGUGAAUGGAAGGAUGCAU | 121 | 22 | 6.7863 | 1.2502 | 2.4404663** |
| miR2597 | UUUGGCUUCUUCGUCGGGUUUGA | 42 | 8 | 2.3556 | 0.4546 | 2.37342499** |
| miR7698 | UUUCUCAUCAAAGUUAUUCUGACA | 36 | 7 | 2.0191 | 0.3978 | 2.34359718** |
| miR3522 | UGAGACCAAAUGAGCAGCUGA | 975 | 201 | 54.6832 | 11.4224 | 2.25923186** |
| miR414 | CAUCUUCAGCAUCACCGUCCA | 29 | 6 | 1.6265 | 0.341 | 2.25392718** |
| miR1509 | UUAAUCAAGGAAAUCACGGUCG | 285 | 59 | 15.9843 | 3.3528 | 2.25321723** |
| miR1091 | GGCGUGAGGAGGAUUUGAC | 95 | 20 | 5.3281 | 1.1366 | 2.22889654** |
| miR8124 | ACUUGGUACGUGGAGCGGU | 301 | 66 | 16.8817 | 3.7506 | 2.17026688** |
| miR3711 | GGCGCUAGAAGGAGGGCAUU | 109 | 25 | 6.1133 | 1.4207 | 2.10534942** |
| miR5293 | CAUGAAAAGUAGGAAGAAGAUGA | 257 | 59 | 14.4139 | 3.3528 | 2.10402241** |
| miR823 | UGGGUAGAGUAGACAUCAUAAGAU | 38 | 9 | 2.1312 | 0.5115 | 2.05885984** |
| miR5664 | AUGAAAAUUUUAUCGGCUG | 37 | 9 | 2.0752 | 0.5115 | 2.02044424** |
| miR418 | UUAAUGUGAUCAUGAAUGAGC | 209 | 51 | 11.7218 | 2.8982 | 2.01596507** |
| miR5049 | UUUAUAUGGAUCGGACGGA | 170 | 43 | 9.5345 | 2.4436 | 1.96414914** |
| miR5999 | CUUCACGAUCAUGACGGACAA | 481 | 122 | 26.9771 | 6.933 | 1.9601836** |
| miR5648 | AUUGAAGAACAAGGAGCGGCAU | 52 | 14 | 2.9164 | 0.7956 | 1.87407343** |
| miR160 | GCGUACGAGGAGCCAAGCAUA | 18548 | 5197 | 1040.2712 | 295.3343 | 1.81653886** |
| miR5227 | UGAAGAUGAAGACGAUGAUGAAGA | 2721 | 768 | 152.6083 | 43.6438 | 1.8059848** |
| miR2661 | UAUGUUUGGAGAAAUUUGGGCAG | 21 | 6 | 1.1778 | 0.341 | 1.78825093** |
| miR2101 | AUUUGAACUCAAGUGACCGUUGGU | 94 | 27 | 5.272 | 1.5344 | 1.78067574** |
| miR5675 | UCAGAGACGGACAACUGGAAA | 31 | 9 | 1.7386 | 0.5115 | 1.7651199** |
| miR5176 | UUGGAUGAAUGAUGUGGCAUAGAU | 112 | 33 | 6.2816 | 1.8753 | 1.74401067** |
| miR5641 | UGGAAGAAGAUGUAGGUUUAA | 44 | 13 | 2.4678 | 0.7388 | 1.73996971** |
| miR6432 | CCGACUUGGAGAAAAAGAUGG | 323 | 97 | 18.1156 | 5.5123 | 1.71650628** |
| miR536 | UUGUUCAAGCUGUGGCAUA | 43 | 13 | 2.4117 | 0.7388 | 1.70679468** |
| miR6192 | UGGAGAGGGGAGGAGAGGGAACUG | 42 | 13 | 2.3556 | 0.7388 | 1.67283881** |
| miR393 | UCAUGCGAUCCCUUCGGAAUU | 1977 | 614 | 110.8807 | 34.8923 | 1.66802767** |
| miR5262 | UCUUCAAGAGACUCAAUUU | 235 | 74 | 13.1801 | 4.2053 | 1.64808069** |
| miR1878 | ACUUAAUUGGCACUAAAAAAGAAA | 19 | 6 | 1.0656 | 0.341 | 1.64382234* |
| miR5255 | UGCUGUAGAUAGAACGGACAUGGG | 28 | 9 | 1.5704 | 0.5115 | 1.61832593** |
| miR1535 | CUUCUUUGUGGUAGAUUGUUU | 1204 | 396 | 67.5268 | 22.5038 | 1.58529156** |
| miR6469 | UGGGAGAAAAGGAUUACCGGUUA | 98 | 33 | 5.4964 | 1.8753 | 1.55136559** |
| miR5209 | CAGGAGGACGGUUUUGUUUGA | 151 | 52 | 8.4689 | 2.955 | 1.51901646** |
| miR5140 | CGCUGGUGGAGGAUUUGGUGG | 37 | 13 | 2.0752 | 0.7388 | 1.48999461** |
| miR3948 | UGGGAGUGUGGAGUGGGAGUGGGA | 5455 | 1938 | 305.9456 | 110.1324 | 1.47403619** |
| miR5632 | UUUAUUGAUAGUUGGAUAAGU | 166 | 61 | 9.3102 | 3.4665 | 1.4253324** |
| miR2870 | UAACAGUUUGGUGGACGACAAA | 938 | 347 | 52.6081 | 19.7193 | 1.41567661** |
| miR2652 | UAUGCAGGGUGGAUUGGAU | 185 | 69 | 10.3758 | 3.9211 | 1.40389224** |
| miR5769 | UGAUAGGGACAAUGAAGAGACGGA | 24 | 9 | 1.346 | 0.5115 | 1.39587227* |
| miR5799 | AGACGAAUGAUCAAAGUUGGAC | 24 | 9 | 1.346 | 0.5115 | 1.39587227* |
| miR4414 | AGCUGCUGACUCGUUGGUUC | 1375 | 532 | 77.1174 | 30.2324 | 1.3509609** |
| miR171 | CGAGCCGAAUCAAUAUCACUC | 6819 | 2654 | 382.446 | 150.8211 | 1.34241778** |
| miR5081 | UUUUGUUGCAGAAUUGAUAGU | 28 | 11 | 1.5704 | 0.6251 | 1.32897317** |
| miR6280 | UUAGCAUGUAAGAUUCUUGGU | 162 | 66 | 9.0858 | 3.7506 | 1.27649214** |
| miR1518 | UGUGUUGUAAAGGGAAUAGUCGAU | 49 | 20 | 2.7482 | 1.1366 | 1.27376238** |
| miR6170 | CAAGAAAAAGUAGAGAUGGGCAU | 142 | 58 | 7.9641 | 3.296 | 1.27279509** |
| miR1507 | UCUCAUUCCAUACAUCGUCUGA | 873 | 361 | 48.9625 | 20.5149 | 1.2550051** |
| miR6460 | CUGAUAUGUGGAUCAUCGA | 185 | 77 | 10.3758 | 4.3757 | 1.24563684** |
| miR5373 | UCUGAUUGUUCUUGAUGAUGU | 80 | 34 | 4.4868 | 1.9321 | 1.21551711** |
| miR530 | UGCAUUUGCACCUGCAUCUUG | 338 | 145 | 18.9569 | 8.24 | 1.20200682** |
| miR5380 | GAGAAUGAGAUGGGGAUGGGGAA | 279 | 122 | 15.6478 | 6.933 | 1.17440817** |
| miR1077 | UCCUCAAAUGUUCGGAAUGACGUG | 25 | 11 | 1.4021 | 0.6251 | 1.16543034* |
| miR2621 | AGCUUGGGCGUUGGAAUUUGGCUA | 34 | 15 | 1.9069 | 0.8524 | 1.16162669** |
| miR8045 | AUUGAUAGUUGUGGCGGUU | 31 | 14 | 1.7386 | 0.7956 | 1.12781087* |
| miR4993 | GGCGGCGGCGGUGGUGGAG | 97 | 44 | 5.4403 | 2.5004 | 1.1215273** |
| miR854 | AGGAUGAGGAAGAGGAGGAGGAGC | 68 | 31 | 3.8138 | 1.7617 | 1.11426092** |
| miR5521 | AGUGGCAUGCUUAUCUGAUGA | 35 | 16 | 1.963 | 0.9092 | 1.11039058** |
| miR5185 | UUUGAAAAAUGAACUGAAGAA | 72 | 33 | 4.0381 | 1.8753 | 1.10655523** |
| miR440 | AUGUCACUGAUGAUCGAAGGACAA | 488 | 225 | 27.3697 | 12.7863 | 1.09798077** |
| miR948 | UGUGGAUGUUGGGUUCGGG | 52 | 24 | 2.9164 | 1.3639 | 1.09645074** |
| miR1172 | UAAGAUGGAGGACGCAGUGAU | 71 | 33 | 3.9821 | 1.8753 | 1.08640804** |
| miR2936 | GCUAGAGAGAGAGAAGCACGAGAG | 150 | 71 | 8.4128 | 4.0348 | 1.06008888** |
| miR5802 | AGUGUGAUUAUUUGUAAAGCGGA | 23 | 11 | 1.29 | 0.6251 | 1.04521216* |
|  |  |  |  |  |  |  |
| ***Down-regulated miRNAs*** | |  |  |  |  |  |
| miR2620 | UUCUGAUAGAACAUCGUGCUCAAGC | 49 | 99 | 2.7482 | 5.626 | -1.03362255** |
| miR5722 | UAGAAAUAGAGUAGUGGUAACGA | 36 | 73 | 2.0191 | 4.1484 | -1.03884264** |
| miR8051 | AUAGUAUGGUAGAAAGAUUCA | 90 | 183 | 5.0477 | 10.3995 | -1.04281609** |
| miR6426 | GAUGGAGACAGUAGGUGAAGA | 158 | 326 | 8.8615 | 18.5259 | -1.0639208** |
| miR774 | UGAGAUGGAAGAUGAUGGUAU | 236 | 495 | 13.2361 | 28.1298 | -1.0876212** |
| miR6285 | UGUGAAAGUGUGAAAUUAGGGCAU | 10 | 21 | 0.5609 | 1.1934 | -1.08926219** |
| miR6483 | UUAUUGUAGAAAUUUUCGGGAU | 40 | 85 | 2.2434 | 4.8304 | -1.10645578** |
| miR390 | AAGCUCAGGAGGGAUAGCGCC | 1027 | 2186 | 57.5997 | 124.2257 | -1.10883047** |
| miR5556 | UGAUGAGGAAGAACUGCAGAA | 15 | 33 | 0.8413 | 1.8753 | -1.15642916** |
| miR5147 | CAACUGUUGUGGAUUUGAGGG | 15 | 34 | 0.8413 | 1.9321 | -1.19947752** |
| miR5152 | AGUCCUGCUAUACCCACCA | 41 | 93 | 2.2995 | 5.285 | -1.20058327** |
| miR3629 | UGGAUGCUAGAAAUUGUAGGA | 14 | 32 | 0.7852 | 1.8185 | -1.21161685** |
| miR5667 | AAACAGAUCAAAGAUGGCAUUUCC | 303 | 711 | 16.9939 | 40.4046 | -1.24950257** |
| miR894 | GUUUCACGUCGGGUUCACCA | 23124 | 54264 | 1296.9178 | 3083.706 | -1.24957818** |
| miR5813 | ACAGCAGGACGGUGGUCAUGGA | 2867 | 6779 | 160.7967 | 385.2359 | -1.26050436** |
| miR5077 | GAUUCACGUCGGGUUCACCA | 1366 | 3272 | 76.6126 | 185.9407 | -1.279189** |
| miR173 | GAUUCUCUGGGCGAAGUGGAAUGC | 25 | 61 | 1.4021 | 3.4665 | -1.30589051** |
| miR5073 | GUUUGGUGAACGGCAGAUAUAUUU | 405 | 1038 | 22.7146 | 58.9873 | -1.37678447** |
| miR5720 | UUUUGAUUGUUGGAAUUC | 87 | 232 | 4.8794 | 13.1841 | -1.43402343** |
| miR396 | CUCAAGAAAGCUGUGGGAGA | 2686 | 7342 | 150.6453 | 417.23 | -1.46968723** |
| miR6477 | AUGAACAGUAACAGUGAAUGGUAU | 8 | 23 | 0.4487 | 1.307 | -1.54243605** |
| miR7716 | UCUGUUCUAGAGAAAACGACUAAC | 8 | 23 | 0.4487 | 1.307 | -1.54243605** |
| miR6283 | AAAAGGGGAGUGGGAGUGGAAAUC | 11 | 32 | 0.6169 | 1.8185 | -1.55964037** |
| miR2670 | UCUCAACAUGGACGUGAUCAGAAUA | 21 | 63 | 1.1778 | 3.5802 | -1.6039456** |
| miR8125 | CAAGUAAAGCAAUGGAUGAGUA | 178 | 535 | 9.9832 | 30.4029 | -1.60663471** |
| miR5658 | GAAUGAUGAUGAUGAUGAUGAUGA | 53 | 165 | 2.9725 | 9.3766 | -1.65738808** |
| miR1092 | UGACAGGAAGCUUGGUGUUUU | 8 | 25 | 0.4487 | 1.4207 | -1.66277885** |
| miR3946 | GUAGAGAGAGAGAGAGAGAGCAA | 135 | 423 | 7.5715 | 24.0382 | -1.66667782** |
| miR403 | UUAGAUUCACGCACAAACUCG | 985 | 3145 | 55.2441 | 178.7236 | -1.69383785** |
| miR5655 | AAGUAGACCACAAAGACGGAA | 14 | 47 | 0.7852 | 2.6709 | -1.76619388** |
| miR841 | UACAGCACUGUGACAACUGAA | 19 | 64 | 1.0656 | 3.637 | -1.77108294** |
| miR528 | UGGAAGGGGCAUGCAGAGGAG | 7 | 24 | 0.3926 | 1.3639 | -1.79660579** |
| miR3446 | CUGGAAGCAACUGUGGCACGG | 2762 | 9695 | 154.9078 | 550.9459 | -1.83050087** |
| miR1441 | ACGGAUGUCGGAUGAAAUGGUUGU | 9 | 33 | 0.5048 | 1.8753 | -1.89333759** |
| miR5368 | AGGGACAGUCUCAGGUAGA | 232 | 852 | 13.0118 | 48.4173 | -1.89570208** |
| miR5522 | AAGAUAGGAAUGGGGAGGCUU | 5 | 19 | 0.2804 | 1.0797 | -1.94507225** |
| miR5794 | UGAGGAAUCACUAGUAGUCGU | 17 | 65 | 0.9535 | 3.6938 | -1.95380091** |
| miR6115 | CUGGACGGGUUUGACAUGGCAU | 6 | 23 | 0.3365 | 1.307 | -1.95758073** |
| miR6455 | UAAAGAUAGCAUCCUCAAUUU | 6 | 23 | 0.3365 | 1.307 | -1.95758073** |
| miR5292 | AUUCAGUAGAGCAACAAAGAAGGC | 35 | 135 | 1.963 | 7.6718 | -1.96650494** |
| miR5830 | UGUGAUAGGUGAUGUUAAACAU | 8 | 31 | 0.4487 | 1.7617 | -1.97314518** |
| miR159 | UUUGGAUUGAAGGGAGCUCUA | 1894 | 7695 | 106.2257 | 437.2902 | -2.04145817** |
| miR5236 | UGAUUCGGCAGAUUUGGAU | 7 | 29 | 0.3926 | 1.648 | -2.06958416** |
| miR6111 | AUGAUGGUGAUUGAAUAGC | 7 | 29 | 0.3926 | 1.648 | -2.06958416** |
| miR5783 | GACGGCGACGGGGAGGAGGCG | 10 | 42 | 0.5609 | 2.3868 | -2.08926219** |
| miR5810 | ACGGAACCGCUAUGGCAAGGCAA | 19 | 82 | 1.0656 | 4.6599 | -2.12863301** |
| miR6485 | AGGAUGUAGAAGAUCAUA | 22 | 96 | 1.2339 | 5.4555 | -2.14448595** |
| miR2616 | UUUGGGUUUGGAUCGGGUUCGGAU | 7 | 32 | 0.3926 | 1.8185 | -2.21161685** |
| miR6173 | CUAGCCGUAAACGAUGGAUA | 49 | 226 | 2.7482 | 12.8431 | -2.22443457** |
| miR5239 | UGGGAAUGAAAAUGAGAGAAUGUG | 40 | 188 | 2.2434 | 10.6836 | -2.25163908** |
| miR6271 | AAUCAAGUUGAGAGAUUAUAAGUG | 5 | 24 | 0.2804 | 1.3639 | -2.28217962** |
| miR164 | UGGAGAAGCAGGGCACGUGCA | 9464 | 45465 | 530.7918 | 2583.6779 | -2.28320824** |
| miR1112 | AACGGAAUGGUAUACAAAGUCA | 26 | 129 | 1.4582 | 7.3308 | -2.32978204** |
| miR5246 | UCUCCAGAGAGUUUGAAGGUU | 22 | 117 | 1.2339 | 6.6489 | -2.4298902** |
| miR7767 | CCCCAAGAUGAGUGCUCUCC | 120 | 658 | 6.7302 | 37.3927 | -2.47403536** |
| miR2643 | UUUGGGACGAGAUUGAGA | 121 | 686 | 6.7863 | 38.9839 | -2.52218131** |
| miR4344 | AAGUAGACAUUUAAUGACGGUUU | 4 | 23 | 0.2243 | 1.307 | -2.54275762** |
| miR7511 | AGAAGAUUUUGUUGUGUAGUGAC | 5 | 29 | 0.2804 | 1.648 | -2.55515799** |
| miR408 | ACGGGGAACAGGCAGAGCAUG | 11319 | 65804 | 634.8302 | 3739.4994 | -2.55840249** |
| miR5501 | ACUUGUGGCUAGGGGUGAA | 37 | 216 | 2.0752 | 12.2748 | -2.56437723** |
| miR8033 | UUUCAAAGCUGGAGAAAGAU | 27 | 160 | 1.5143 | 9.0925 | -2.58602597** |
| miR5656 | AGUGAGUGAGAGAUUGGGUGU | 46 | 276 | 2.5799 | 15.6845 | -2.60395249** |
| miR7781 | CGAUUAUCUGACACGGACAUGAC | 4 | 25 | 0.2243 | 1.4207 | -2.66310042** |
| miR7782 | ACUUGCUCUGAUACCAGCUGAUGA | 3 | 20 | 0.1683 | 1.1366 | -2.75561754** |
| miR7130 | GAUGGAAUGUGGAGGAUGUGUGU | 12 | 84 | 0.673 | 4.7735 | -2.82636905** |
| miR6149 | UUAUAGGAACCUGAACUCGGC | 5 | 36 | 0.2804 | 2.0458 | -2.86710686** |
| miR1026 | UGAGAAAGAUAGAGAGGACGA | 61 | 440 | 3.4212 | 25.0043 | -2.86960187** |
| miR5534 | CUUUAGACAACAGUAGAAUGG | 1437 | 10562 | 80.5947 | 600.2157 | -2.89672418** |
| miR4995 | UAGGCAGUGGCUUGGUUAAGGGAACC | 38 | 293 | 2.1312 | 16.6506 | -2.96583627** |
| miR2949 | CACUUUUGAAAUCUGGAUUGCCGC | 3 | 24 | 0.1683 | 1.3639 | -3.01863079** |
| miR5303 | UUAUGAAGAGUUGAGCAUC | 8 | 67 | 0.4487 | 3.8075 | -3.08502095** |
| miR2626 | AUGUUGGGAUUUAGGUGUU | 5 | 46 | 0.2804 | 2.6141 | -3.22075608** |
| miR5025 | AUCUGUAUAUAUGGGUAAUGAUCA | 26 | 243 | 1.4582 | 13.8092 | -3.24336923** |
| miR7708 | AGAAUGGACUGAAAUUGUAAUGGU | 4 | 39 | 0.2243 | 2.2163 | -3.30465165** |
| miR158 | UCCCAAAUGUAGACAAAGCA | 19 | 192 | 1.0656 | 10.9109 | -3.35603222** |
| miR1877 | AGAUGACAGUGGAUAUGGAGGGGG | 16 | 162 | 0.8974 | 9.2061 | -3.35876702** |
| miR8005 | UUAGGUUUAGGGUUUAGGGUUUA | 36 | 373 | 2.0191 | 21.1968 | -3.39206221** |
| miR7842 | UAAUCAAGCCGGGUACUUCUA | 5 | 54 | 0.2804 | 3.0687 | -3.45206936** |
| miR5260 | UUUGUAUGUGGAAAUGGCU | 2 | 23 | 0.1122 | 1.307 | -3.54211456** |
| miR7760 | CGGCUUUGGUCGGAGUGGUGGGC | 20 | 233 | 1.1217 | 13.2409 | -3.56124241** |
| miR4398 | UCACCGGAGGAGAAAGACGAAA | 3 | 39 | 0.1683 | 2.2163 | -3.7190461** |
| miR3979 | UUCGGGGGAGGUGAGAAG | 29 | 437 | 1.6265 | 24.8338 | -3.93246231** |
| miR3933 | AGAAGAAAAUGAAGAACACGG | 4 | 61 | 0.2243 | 3.4665 | -3.94997823** |
| miR6214 | CGACACGAAGCAGACACGACA | 83 | 1291 | 4.6551 | 73.3647 | -3.978202** |
| miR7743 | AAUUUACUGAUAUAGUUUGACUGC | 2 | 32 | 0.1122 | 1.8185 | -4.01860434** |
| miR7539 | AGAGAGAGAGAGGACAAGG | 18 | 291 | 1.0095 | 16.5369 | -4.033976** |
| miR5834 | ACGGAUGAGAAAAUGGUGU | 29 | 484 | 1.6265 | 27.5047 | -4.07983544** |
| miR5210 | AUAAGUGCGUUUGGAAUUAAGGUU | 11 | 185 | 0.6169 | 10.5132 | -4.09102141** |
| miR5712 | AAUUAUUAAUUAAUUGAGUGUAGA | 4 | 69 | 0.2243 | 3.9211 | -4.12775691** |
| miR1069 | UUCAAGGCUUAGCAUUGGA | 3 | 52 | 0.1683 | 2.955 | -4.13405105** |
| miR6180 | AGGGAGAAGAAAGGAGCG | 13 | 226 | 0.7291 | 12.8431 | -4.13873296** |
| miR2614 | CGGUUCGAUUCGGUUUGGUUC | 24 | 428 | 1.346 | 24.3223 | -4.17552935** |
| miR2867 | CACAGGACGUUCGGAGUGGCA | 6 | 114 | 0.3365 | 6.4784 | -4.26695914** |
| miR5791 | UAUGCAGGAGAUCUAGAUAACAG | 1 | 20 | 0.0561 | 1.1366 | -4.34058004** |
| miR4401 | ACAAGUAUUUAAAGUAGCUGCAUU | 4 | 82 | 0.2243 | 4.6599 | -4.37679747** |
| miR847 | CUUGAAUGUAAGUAUGGAAUGGAA | 5 | 104 | 0.2804 | 5.9101 | -4.39762429** |
| miR6032 | UGGAGAUCAACAGAAUGUGG | 2 | 42 | 0.1122 | 2.3868 | -4.4109331** |
| miR2586 | CGGGUUGGAUCGGGUUGGAC | 2 | 44 | 0.1122 | 2.5004 | -4.47801433** |
| miR7980 | CUGAGAUGGAAGAAUGUUUGGA | 2 | 45 | 0.1122 | 2.5573 | -4.51047683** |
| miR5789 | UGAUGAGCAUCUGGUCGGUAU | 7 | 160 | 0.3926 | 9.0925 | -4.53354494** |
| miR5240 | UUGAAAAUAAAUUGUUGGAAUUUAA | 3 | 74 | 0.1683 | 4.2053 | -4.64310164** |
| miR7512 | UGUUACUUGUAGAUGCAUA | 1 | 25 | 0.0561 | 1.4207 | -4.66245736** |
| miR2592 | UACGGCAGAGACUCAGCAAUUCGA | 1 | 27 | 0.0561 | 1.5344 | -4.77353004** |
| miR6449 | CAUGCUCUCUGAAAUUAAACGGUUU | 18 | 493 | 1.0095 | 28.0161 | -4.79454332** |
| miR5290 | AAUUUGGAUGAGAGUGACCUA | 14 | 394 | 0.7852 | 22.3902 | -4.83366343** |
| miR6259 | UAGAAAAUCGGGCGACACAA | 2 | 63 | 0.1122 | 3.5802 | -4.9958956** |
| miR1526 | AGGAAGAGGAAAAUUAAGCAA | 2 | 65 | 0.1122 | 3.6938 | -5.04096117** |
| miR838 | UGUAGAAUGAGAAGAAGGC | 12 | 438 | 0.673 | 24.8906 | -5.20885069** |
| miR7758 | CAUUGACCGUUAGUUGACCGUG | 30 | 1104 | 1.6826 | 62.7379 | -5.22057308** |
| miR8036 | UUUCUUCCCUAUGCCUCCCA | 18 | 719 | 1.0095 | 40.8592 | -5.33894814** |
| miR4371 | AUAGUGGUGACGGGUGACGGAGA | 25 | 1028 | 1.4021 | 58.419 | -5.38077651** |
| miR4385 | AAGUCAUGUAGAAUAAGGAUGGU | 2 | 87 | 0.1122 | 4.944 | -5.46153416** |
| miR5492 | AGACUAGGAGAAACAGAUAUGGUU | 13 | 575 | 0.7291 | 32.676 | -5.48597088** |
| miR5226 | UUUUACAAGUUGGAGGCUCA | 3 | 138 | 0.1683 | 7.8422 | -5.54215135** |
| miR5554 | UUGCAUCAUGGGAACAAAUGGUAU | 1 | 47 | 0.0561 | 2.6709 | -5.57318138** |
| miR5996 | UGACUCCAGAUCAGAAGGUUG | 1 | 47 | 0.0561 | 2.6709 | -5.57318138** |
| miR6228 | UGGAUAGUAGAAUAAUGAAGGAGA | 14 | 674 | 0.7852 | 38.302 | -5.60821574** |
| miR7739 | UUGAGCUGAGAAGGAUUUU | 2 | 106 | 0.1122 | 6.0238 | -5.74652929** |
| miR5259 | CAAGGGGUAUUUGGAUGGACA | 9 | 507 | 0.5048 | 28.8117 | -5.83479907** |
| miR4413 | GAGAGGAUUGUAAGUCACGUGC | 4 | 243 | 0.2243 | 13.8092 | -5.94405631** |
| miR5759 | AACGGGGUGAAGAAGAUUAGA | 2 | 137 | 0.1122 | 7.7854 | -6.11662658** |
| miR2864 | UUGUUUUGAUUCGUUAGGA | 3 | 231 | 0.1683 | 13.1272 | -6.28538024** |
| miR5499 | GAAGGAAGAGAUCUUCGGAA | 4 | 309 | 0.2243 | 17.5598 | -6.29070298** |
| miR8003 | UUUCUGGUAACAAAUGGGAGUC | 3 | 250 | 0.1683 | 14.207 | -6.39942296** |
| miR2086 | UCACAUUUAAUGCAGAACUGGAA | 2 | 169 | 0.1122 | 9.6039 | -6.4194758** |
| miR7731 | GGUUAGCACUCUGGACUUUGAAUC | 0 | 18 | 0.01 | 1.0229 | -6.6765213** |
| miR2585 | CGGAUUAAUGAUUACAGGGAC | 0 | 19 | 0.01 | 1.0797 | -6.7544867** |
| miR6260 | UGGAGUGGGAGUGGGAGU | 55 | 6237 | 3.0847 | 354.4353 | -6.8442483** |
| miR2097 | UCUCUCUCUCUUCUGUCGGCUUUU | 0 | 21 | 0.01 | 1.1934 | -6.89893387** |
| miR8040 | UUAUAAUUGUAAUAUCUGAUG | 0 | 21 | 0.01 | 1.1934 | -6.89893387** |
| miR833 | UGAUUUGUUGAUAAUCGGCUCAGU | 3 | 370 | 0.1683 | 21.0263 | -6.96501601** |
| miR400 | AUGAGCAGUAGGUUAAUAAGUCAC | 0 | 22 | 0.01 | 1.2502 | -6.9660151** |
| miR5137 | AGGGAGAAGGACAUCGGCU | 0 | 22 | 0.01 | 1.2502 | -6.9660151** |
| miR5649 | AAUUGAUUGUUGGAUUACUAU | 4 | 514 | 0.2243 | 29.2095 | -7.02486423** |
| miR5503 | UUGGAUUUUUUUAAGGCAUU | 0 | 23 | 0.01 | 1.307 | -7.03011533** |
| miR3513 | UUAGAUAAGAUAGGAUUGGAU | 2 | 261 | 0.1122 | 14.8321 | -7.04650639** |
| miR7991 | AGGAGGUGGAUUUUUAAUUGUAUA | 0 | 24 | 0.01 | 1.3639 | -7.09159406** |
| miR6247 | UGGCUGAAUGAACAUAAGGCA | 5 | 694 | 0.2804 | 39.4385 | -7.13597452** |
| miR2595 | UACAUUUCUCUUCAAUUUAUUCUC | 0 | 29 | 0.01 | 1.648 | -7.36457243** |
| miR2905 | UUACAUGACAGGACAAAGCA | 0 | 29 | 0.01 | 1.648 | -7.36457243** |
| miR7510 | AAGGGCAUGUUUUAGCGGACAUU | 0 | 30 | 0.01 | 1.7048 | -7.41345869** |
| miR946 | UAUGGAUGAGAAGGGUUU | 0 | 31 | 0.01 | 1.7617 | -7.46082446** |
| miR916 | CAAGGUCAUCGGUUCGAUU | 0 | 32 | 0.01 | 1.8185 | -7.50660512** |
| miR2950 | UGGUAGACGGAGAUGGAAUA | 1 | 188 | 0.0561 | 10.6836 | -7.57318138** |
| miR7488 | UUUUUGAUAAAGUGGACAAAA | 0 | 38 | 0.01 | 2.1595 | -7.75455351** |
| miR5773 | UUUUUAAAGUUUCCGUUAGU | 0 | 41 | 0.01 | 2.3299 | -7.86412423** |
| miR4351 | AUUGGGAGUGUCGAGUGGGAGUGG | 20 | 4700 | 1.1217 | 267.0909 | -7.89550014** |
| miR1149 | CGGAUCAAUACUGACCCCCAGC | 0 | 43 | 0.01 | 2.4436 | -7.93286434** |
| miR1866 | UGAAAUUCCUGUGAAAGAUUGCGU | 0 | 46 | 0.01 | 2.6141 | -8.03017052** |
| miR2919 | AAUGGGAGGGGGGGGCAAGAA | 0 | 47 | 0.01 | 2.6709 | -8.06118215** |
| miR6230 | UGACAAGUUUAAGGGCAUUAGA | 0 | 47 | 0.01 | 2.6709 | -8.06118215** |
| miR851 | UUGGGUGGAAAAAAGAAGACGA | 0 | 50 | 0.01 | 2.8414 | -8.15045813** |
| miR7821 | AGAUGGGAUGGGGAUUGUA | 0 | 55 | 0.01 | 3.1255 | -8.28794319** |
| miR6465 | UUAAAGCUGAAGGGGUUAGUAGAU | 0 | 56 | 0.01 | 3.1824 | -8.31397137** |
| miR6189 | AGGGAGGCUGGUGGUGAUC | 0 | 58 | 0.01 | 3.296 | -8.36457243** |
| miR7490 | AGUGUAGAAGACUUCACCUGGGU | 0 | 58 | 0.01 | 3.296 | -8.36457243** |
| miR7981 | UUAAUUAUACUAUGGUCGGCUAUU | 0 | 65 | 0.01 | 3.6938 | -8.52896194** |
| miR8004 | GAGUGGAUUGUGUAUGUGUUGGCU | 0 | 70 | 0.01 | 3.9779 | -8.6358632** |
| miR7734 | UUGAACGUUGACUGGUUGUAAGG | 0 | 79 | 0.01 | 4.4894 | -8.81037883** |
| miR5263 | AGGCUAAAACAGAACGGGG | 0 | 82 | 0.01 | 4.6599 | -8.86415519** |
| miR1110 | GACUUAGGGAGACAGUGGUAAGGA | 0 | 89 | 0.01 | 5.0577 | -8.98233766** |
| miR5078 | CUUAGUUGACGUGGCAUUU | 0 | 92 | 0.01 | 5.2282 | -9.03017052** |
| miR4387 | AGAGUGAUGAGUAGACAUCCCUC | 1 | 524 | 0.0561 | 29.7778 | -9.05202068** |
| miR6292 | UACUUUUAACGUUAUUUCA | 0 | 95 | 0.01 | 5.3986 | -9.07644152** |
| miR7983 | UAAAGCUUUAGGACAUUGGU | 0 | 108 | 0.01 | 6.1374 | -9.2614838** |
| miR4357 | CAGUCGGUUUGGAUCGGUUCAU | 0 | 118 | 0.01 | 6.7057 | -9.38924413** |
| miR6187 | UGAAUAGUUCGGCACUCUCA | 0 | 130 | 0.01 | 7.3876 | -9.52896194** |
| miR5023 | UUGGUAGUGAUAAAGGUGC | 0 | 137 | 0.01 | 7.7854 | -9.60462735** |
| miR5268 | AUAGAGUGGAAUGAGAAUGUGUU | 0 | 140 | 0.01 | 7.9559 | -9.63588133** |
| miR771 | AGGAUCCUCAGAUGUUGCAU | 0 | 155 | 0.01 | 8.8083 | -9.7827198** |
| miR1165 | UACUGUAGCAAGCGGCCAUCC | 0 | 166 | 0.01 | 9.4334 | -9.88163403** |
| miR6138 | UCACGUUUGGGAUUGAGGUUGAAA | 0 | 175 | 0.01 | 9.9449 | -9.95781305** |
| miR1516 | CAAAAGAGAUUGGGCUUUA | 0 | 183 | 0.01 | 10.3995 | -10.02229845** |
| miR782 | ACAAACAGAGUUGGAUUUCUU | 0 | 191 | 0.01 | 10.8541 | -10.08402439** |
| miR2663 | UUAGAGAGGGUUUAAAAUUU | 0 | 198 | 0.01 | 11.2519 | -10.13595292** |
| miR6245 | GGAAUAGGUGUCGGCUCAU | 0 | 207 | 0.01 | 11.7634 | -10.20008939** |
| miR4369 | GGAUGCAAGCGUUAUCCGGAAUGA | 0 | 233 | 0.01 | 13.2409 | -10.37078547** |
| miR6470 | CUCUGAUGAUUCAUUAUUAGAUAAAA | 0 | 269 | 0.01 | 15.2867 | -10.57806128** |
| miR7713 | AGGAAUUGACGGAACAGCACACCAG | 4 | 6071 | 0.2243 | 345.0018 | -10.58695855** |
| miR7841 | GGGGAUUCUCUCAAGCAAA | 0 | 276 | 0.01 | 15.6845 | -10.61512382** |
| miR5067 | UCAGAGAACAACUAGAUAGGAU | 0 | 291 | 0.01 | 16.5369 | -10.6914731** |
| miR7128 | AUAUUAUGACUUAUAACGA | 0 | 337 | 0.01 | 19.151 | -10.90320401** |
| miR2938 | AUCUUCUGAGAAGGGUUCGAG | 0 | 344 | 0.01 | 19.5488 | -10.93286434** |
| miR2921 | UAAGAACUUAAUAGAAUUUGAGC | 0 | 376 | 0.01 | 21.3673 | -11.0611889** |
| miR7837 | UGGAUGGGAGGAUGUGGUGU | 0 | 418 | 0.01 | 23.754 | -11.21395476** |
| miR2868 | UUGGUUUUGGUGAGUAGGGAA | 0 | 427 | 0.01 | 24.2655 | -11.24469087** |
| miR7838 | AAGCAUGUGCUGGGAGGAGAGAGA | 0 | 606 | 0.01 | 34.4377 | -11.74977308** |
| miR2648 | UAGCAUGGGAUAACAGAU | 0 | 611 | 0.01 | 34.7218 | -11.76162602** |
| miR4244 | UGGUUGAUUGUUGGAAUUG | 0 | 694 | 0.01 | 39.4385 | -11.94538897** |
| miR6195 | UGAGAAGUAGAAAGGGAUGAG | 4 | 18928 | 0.2243 | 1075.6374 | -12.22747458** |
| miR8042 | AUAGACUGAAGAUGCAUUGUAUCU | 0 | 887 | 0.01 | 50.4063 | -12.29938834** |
| miR5821 | UGACGGAGCAUGGUGAGG | 0 | 983 | 0.01 | 55.8618 | -12.44764635** |
| miR2664 | AAUGUGAGUGGGGUUGACGUC | 0 | 1501 | 0.01 | 85.2986 | -13.05830635** |
| miR911 | CAAGUGGAGUACGGACAUUU | 0 | 1725 | 0.01 | 98.028 | -13.25897817** |
| miR7692 | UGACUUGGCAGCAUAUCAGUGGAC | 0 | 2062 | 0.01 | 117.179 | -13.51642642** |
| miR5762 | UCGUGAGGAAUAAACUGGC | 0 | 2377 | 0.01 | 135.0798 | -13.72152433** |
| miR5338 | UGAGGUAGUUGGUUGUAU | 0 | 9056 | 0.01 | 514.6329 | -15.65125607** |
| miR401 | CGAACCCUGCUGUCGACGACA | 0 | 10503 | 0.01 | 596.8628 | -15.86511172** |
|  |  |  |  |  |  |  |
| ***Equally expressed miRNAs*** | |  |  |  |  |  |
| miR5490 | UAUGGAUUUGUAUUUGGAUGG | 1315 | 653 | 73.7522 | 37.1086 | 0.99093251 |
| miR5013 | UUUGUGACAGUCAGAGUGCCUU | 322 | 161 | 18.0595 | 9.1493 | 0.98102468 |
| miR5740 | UGGAACAGGAAACAACAUUUGG | 294 | 148 | 16.4891 | 8.4105 | 0.97124918 |
| miR2092 | CAGAAGUCGGGGUUUACU | 999 | 515 | 56.0293 | 29.2663 | 0.9369411 |
| miR1169 | UCUCCAGUCUAAAGCAACAUCACA | 27 | 14 | 1.5143 | 0.7956 | 0.92853587 |
| miR1144 | UGGGUUUAUGUGCGGCAGGCAG | 318 | 170 | 17.8351 | 9.6607 | 0.88451967 |
| miR5079 | UUUGGAUAUGUUAUGUUUGGUGGU | 144 | 78 | 8.0763 | 4.4326 | 0.86554132 |
| miR6114 | UGAAAGGAAUCAGAAUCGUCA | 74 | 41 | 4.1503 | 2.3299 | 0.83294759 |
| miR1314 | UAGGCCAUAGAAUGUUAGGAGACA | 128 | 71 | 7.1789 | 4.0348 | 0.83126564 |
| miR5673 | UCUUGGAAUCUCGGGAAGCAU | 27 | 15 | 1.5143 | 0.8524 | 0.82904855 |
| miR5826 | UAGGGAAAGUAGAAGAGUGGAGU | 113 | 63 | 6.3376 | 3.5802 | 0.82389642 |
| miR1108 | UGAGCAAAUGAGAGAAUGAACCCC | 23 | 13 | 1.29 | 0.7388 | 0.80411529 |
| miR1511 | AACCAGGCUCUGAUACCAUGA | 58 | 33 | 3.253 | 1.8753 | 0.79464941 |
| miR845 | UGGCUCUGAUACCAAACUGAUGCA | 65 | 37 | 3.6455 | 2.1026 | 0.79394228 |
| miR861 | CUUGGAGAAAUUAUGAGCGUCAGA | 1224 | 699 | 68.6485 | 39.7227 | 0.78926451 |
| miR5203 | AGACUUAUCUAUUGGACGGAGGGA | 21 | 12 | 1.1778 | 0.6819 | 0.78846249 |
| miR6442 | CCAGAACGGUUGAAGGACACG | 286 | 165 | 16.0404 | 9.3766 | 0.77457332 |
| miR6196 | AGGAGAGGUAUAGAUGGACGAGGA | 197 | 114 | 11.0488 | 6.4784 | 0.77018024 |
| miR5657 | UGGACAAGGAAGAUUGAGGUG | 2240 | 1302 | 125.6312 | 73.9899 | 0.76379454 |
| miR2105 | UUGAUGAUGUGAAUAAAUUCA | 78 | 46 | 4.3747 | 2.6141 | 0.74286975 |
| miR5169 | UUUGACCAAAGUUUGAGAGACAUU | 93 | 55 | 5.2159 | 3.1255 | 0.73882921 |
| miR8024 | UUAAAAUUUAAAGGACGUCAACUC | 49 | 29 | 2.7482 | 1.648 | 0.73777076 |
| miR5250 | UGAUGCAUGUUGAUACGGAUC | 778 | 462 | 43.6344 | 26.2545 | 0.73290124 |
| miR5039 | CCCUUUCUGAAUCGUUUGCUG | 106 | 63 | 5.945 | 3.5802 | 0.73163663 |
| miR5179 | UCUUGCUCAAGACCGCGCAAU | 333 | 198 | 18.6764 | 11.2519 | 0.73104776 |
| miR1886 | GAGGAGUGAAAAUCUUAUGAUUGG | 20 | 12 | 1.1217 | 0.6819 | 0.71805479 |
| miR7497 | ACAUGUGACUGUAGCAUAGGCUU | 40 | 24 | 2.2434 | 1.3639 | 0.71794901 |
| miR7773 | UUAUUCCAUUCGUCGACACGU | 382 | 231 | 21.4246 | 13.1272 | 0.70670904 |
| miR5291 | GUGGAUUGAUGGAUUGGAUUGGAU | 512 | 311 | 28.7157 | 17.6735 | 0.70025196 |
| miR6286 | UUUAACCAUUGAUCGUCGUUGAC | 346 | 211 | 19.4055 | 11.9907 | 0.69454972 |
| miR6206 | GGCACACGUGCUUGCAGGUAG | 39 | 24 | 2.1873 | 1.3639 | 0.68141324 |
| miR5143 | UUGGUAUUGGCAAUGUCGGUAA | 55 | 34 | 3.0847 | 1.9321 | 0.67496042 |
| miR6108 | UAUGGGUGAGAAGGGAAGAUA | 76425 | 47247 | 4286.3234 | 2684.9451 | 0.67484812 |
| miR7484 | UUUGAUCAUCAAGAUAAAGAGCAA | 29 | 18 | 1.6265 | 1.0229 | 0.66910571 |
| miR4352 | AAUAAGUAGACAUUUAAUGACGG | 32 | 20 | 1.7947 | 1.1366 | 0.65901808 |
| miR5523 | UAGGAGUGUAACAUAUUUAAUAGU | 19 | 12 | 1.0656 | 0.6819 | 0.6440339 |
| miR5539 | AAGAAAACGGGAUGGCGAGCU | 238 | 151 | 13.3483 | 8.581 | 0.63743833 |
| miR4364 | CGUAGAUCGGCAGCGGAAGAAGUU | 195 | 124 | 10.9366 | 7.0467 | 0.6341446 |
| miR3630 | GCAACUGAUGAUAGAACAGACAGGG | 102 | 65 | 5.7207 | 3.6938 | 0.63108594 |
| miR831 | GAGGAAGAGGAAAGGGAGAUGAGA | 161 | 103 | 9.0297 | 5.8533 | 0.62542783 |
| miR8126 | UCUGACUCCCAGAUUACUGACAUA | 137 | 88 | 7.6837 | 5.0009 | 0.61961344 |
| miR6157 | AGGUAAUGUAGGAUUUGCAAGA | 90 | 58 | 5.0477 | 3.296 | 0.61490993 |
| miR5713 | UAUGAGCUUCAGAAGAACUUUGUU | 195 | 126 | 10.9366 | 7.1603 | 0.61107236 |
| miR6182 | UGGGUUGUAGAGGAUGGCUUU | 34 | 22 | 1.9069 | 1.2502 | 0.60907028 |
| miR2600 | AUAGCAAUUCUGGCUGUGAUUGGU | 105 | 68 | 5.889 | 3.8643 | 0.60781557 |
| miR5337 | UCUGGAACUGCAAGAAUUUGA | 43 | 28 | 2.4117 | 1.5912 | 0.59993528 |
| miR5557 | AAUACAAGACUCAAGGAAGCACAU | 367 | 243 | 20.5833 | 13.8092 | 0.57584456 |
| miR6152 | UUUGAUACGACUGUCAUUUACGG | 24 | 16 | 1.346 | 0.9092 | 0.56600882 |
| miR1074 | AGUGUUUGUUUGUUUGUUGAU | 45 | 30 | 2.5238 | 1.7048 | 0.56599509 |
| miR4393 | UUGAAUAAGGGACACAGAGAC | 480 | 320 | 26.921 | 18.1849 | 0.56599101 |
| miR5298 | UGAGGAAAUGAAUAUGAAGACAA | 494 | 330 | 27.7062 | 18.7532 | 0.56307206 |
| miR8138 | UAAAGAUGGGAACAAAACAA | 431 | 290 | 24.1728 | 16.4801 | 0.5526596 |
| miR1513 | UUUAAUGUGUAGAGAUUCAAUGGU | 120 | 81 | 6.7302 | 4.6031 | 0.54804359 |
| miR5231 | UUUGCAAGUUGUAAGCUCAU | 40 | 27 | 2.2434 | 1.5344 | 0.54801225 |
| miR3704 | GGUAUGGUGAGUAGGGAAGA | 77 | 52 | 4.3186 | 2.955 | 0.54740557 |
| miR4378 | AUAGGACUACUUAGAAUGGCGA | 81 | 56 | 4.5429 | 3.1824 | 0.51349837 |
| miR6462 | AAAGGACAAAAUAUGCAUGAAGA | 39 | 27 | 2.1873 | 1.5344 | 0.51147648 |
| miR5278 | AAAAUAUGCAUGCAGAAAUGUGAA | 49 | 34 | 2.7482 | 1.9321 | 0.50831723 |
| miR5040 | AUUAUACAUAUAAGAAGCAUGAG | 23 | 16 | 1.29 | 0.9092 | 0.50470148 |
| miR8154 | CAGAGAGGAAGGAUGAAGAGGGGC | 33 | 23 | 1.8508 | 1.307 | 0.50188986 |
| miR7996 | AUGUGGAGAAUGAAAUUAUGAAGA | 43 | 30 | 2.4117 | 1.7048 | 0.50044796 |
| miR482 | AGUGGGAGCGUGGGGUAAGAAG | 8665 | 6079 | 485.9796 | 345.4565 | 0.4923917 |
| miR2604 | AAUAUUUUUGUGGGAGUUU | 92 | 65 | 5.1599 | 3.6938 | 0.48223735 |
| miR4406 | AUUGUACUAGAGAACCGGUGUAAC | 965 | 683 | 54.1224 | 38.8134 | 0.479671 |
| miR3436 | CAACAAGUUUUUGGCGCCGUU | 62 | 44 | 3.4773 | 2.5004 | 0.47580863 |
| miR4391 | UAUGGCAAAGAACAAGAAGAA | 18 | 13 | 1.0095 | 0.7388 | 0.45038514 |
| miR1861 | UGAUCUUGAGGCAAGAAGCUGU | 943 | 683 | 52.8885 | 38.8134 | 0.44639924 |
| miR1444 | UGCCACAUCAUCGGUCAAUGUC | 441 | 320 | 24.7336 | 18.1849 | 0.44373125 |
| miR5269 | AGAAGAUGGUGGGACAACUUGCUU | 467 | 339 | 26.1919 | 19.2646 | 0.44316849 |
| miR829 | UAGCUCUGAUACCAAACUGAUGCA | 22 | 16 | 1.2339 | 0.9092 | 0.44055589 |
| miR6233 | UCAAGUUGUUUUGGAAUUACUG | 1137 | 830 | 63.7691 | 47.1671 | 0.43507662 |
| miR1875 | AGCAAUGGAGCUGAAUGAAGAGAA | 30 | 22 | 1.6826 | 1.2502 | 0.42853334 |
| miR8049 | CAAGCCUCUGAAGACAUGCUA | 34 | 25 | 1.9069 | 1.4207 | 0.42462725 |
| miR7129 | AGAAAUCUAGAGAUCGUGUAU | 1157 | 855 | 64.8908 | 48.5878 | 0.41741984 |
| miR5653 | CUGGGUUGGGUAGAGUUGGUUGC | 58 | 43 | 3.253 | 2.4436 | 0.41276268 |
| miR4345 | UUAAGACGGAACUUACAAAGAAUA | 31 | 23 | 1.7386 | 1.307 | 0.41166691 |
| miR5485 | UGACAAUGAAGUAGCAGUAGACAA | 110 | 82 | 6.1694 | 4.6599 | 0.40483119 |
| miR5721 | AUAAAAUGGAUGAGAACCGAUGGA | 48 | 36 | 2.6921 | 2.0458 | 0.39606689 |
| miR5198 | GGGAGAAAGAGAGAUUGUUGGGAG | 331 | 250 | 18.5643 | 14.207 | 0.38592897 |
| miR1134 | CAGAACAAAGAAGAAGAAGAAGAU | 274 | 207 | 15.3674 | 11.7634 | 0.38556799 |
| miR835 | AUGAGAAGAUGGACGCAAGAAAGC | 29 | 22 | 1.6265 | 1.2502 | 0.37961191 |
| miR2916 | UGGGGGCUCGAAGACGAUCAG | 7083 | 5376 | 397.2526 | 305.5065 | 0.37885359 |
| miR5644 | GUGAAGUGGCGGAUAACGGUA | 162 | 123 | 9.0858 | 6.9898 | 0.37836237 |
| miR3947 | AAUGAUUUAGUAGACGACGUUACA | 129 | 99 | 7.235 | 5.626 | 0.36288346 |
| miR5376 | UGAGAGGGUUUGAAGAAUUUGGGC | 720 | 553 | 40.3815 | 31.4258 | 0.36174503 |
| miR1445 | UCCCUUGUAGAUCUAGUAGAAGAA | 4039 | 3109 | 226.5288 | 176.6778 | 0.35857371 |
| miR6295 | AGGACAGGAGAUGAUUCAUGA | 1161 | 902 | 65.1151 | 51.2587 | 0.34519525 |
| miR6029 | UGGGUUGUAAUUUGAUGGCUU | 709 | 558 | 39.7645 | 31.7099 | 0.3265477 |
| miR5719 | UUGUGAUGAAAAUAGACGUCC | 4047 | 3187 | 226.9774 | 181.1103 | 0.32568006 |
| miR6269 | UGUGAAUAAGUGAUUGUCUGA | 52 | 41 | 2.9164 | 2.3299 | 0.32392057 |
| miR1520 | GUCACGAUCCUGUUGGACUAA | 665 | 525 | 37.2968 | 29.8346 | 0.32206542 |
| miR5537 | AAUGAUGUAGGAUCGUCUUGU | 54 | 43 | 3.0286 | 2.4436 | 0.3096429 |
| miR1171 | AGUGUGGAGUGGGAGUGGGAGUGG | 4842 | 3867 | 271.5653 | 219.7533 | 0.30541432 |
| miR7485 | AAAGACCAUCUUUGAUUCGUUUGA | 453 | 362 | 25.4067 | 20.5717 | 0.30454798 |
| miR5054 | GUUCCCCACAGACGGCGCCA | 4772 | 3815 | 267.6393 | 216.7982 | 0.3039372 |
| miR5163 | GUAGAAUAGUUCAGGUGUGUGGGA | 20 | 16 | 1.1217 | 0.9092 | 0.30301729 |
| miR832 | UUACUCCCAAUCCAAGACAAAG | 144 | 117 | 8.0763 | 6.6489 | 0.28057882 |
| miR5818 | UCGAACUGAGAGGCACAGGUU | 540 | 441 | 30.2861 | 25.0611 | 0.27320607 |
| miR4375 | AGCACUAUGGUCGGGCCUGGCAU | 33 | 27 | 1.8508 | 1.5344 | 0.27047438 |
| miR8034 | AUAUGACAGAAGAUCUUCAAAAACU | 293 | 243 | 16.433 | 13.8092 | 0.25096614 |
| miR2948 | UAGUGGGAGAUUGUUGGGAAAAU | 194 | 161 | 10.8806 | 9.1493 | 0.25002484 |
| miR818 | AGGAGCAUUAGGAUGGACCA | 790 | 657 | 44.3074 | 37.3359 | 0.24698416 |
| miR5058 | AACAAUUAGGGAUGACAAACAGCA | 18 | 15 | 1.0095 | 0.8524 | 0.24403841 |
| miR8151 | UGGAUACCAGUAGACCCCAU | 137 | 115 | 7.6837 | 6.5352 | 0.2335698 |
| miR1858 | GUGAUGAGGAGGAGUGGGGUC | 514 | 432 | 28.8279 | 24.5496 | 0.23176623 |
| miR2610 | AAGAUUUAGACAUUGUAUGGCGUU | 1486 | 1259 | 83.3428 | 71.5463 | 0.22018041 |
| miR815 | AAGGGUGAUGAGGAGGAGUGGG | 186 | 158 | 10.4319 | 8.9788 | 0.2164074 |
| miR6427 | GUGGAGAAUGAAAUUAUGAAGAUC | 40 | 34 | 2.2434 | 1.9321 | 0.21551711 |
| miR5386 | CGUCGGCUGUCGGCGGACUG | 2960 | 2537 | 166.0127 | 144.1722 | 0.20350061 |
| miR7814 | AAUUGAUUUUUAUAGCUUUGA | 84 | 72 | 4.7112 | 4.0916 | 0.20342947 |
| miR5835 | ACAAACUCAUCCGGAUUUUGGCAU | 71 | 61 | 3.9821 | 3.4665 | 0.20004969 |
| miR844 | UGUGUAGAGAUUGCUUAUAACGU | 388 | 334 | 21.7611 | 18.9805 | 0.19723349 |
| miR2275 | AGAAUUGGCAAGGAACAAAAGUGA | 22 | 19 | 1.2339 | 1.0797 | 0.19259497 |
| miR2636 | UUUGUGUUGAAGAUGGCUGAAUAU | 1239 | 1072 | 69.4898 | 60.9194 | 0.1898995 |
| miR8148 | UAGACGGAUCGAUGACGUGGCAU | 1414 | 1234 | 79.3047 | 70.1256 | 0.17746516 |
| miR5149 | GAGCAGCUGGAAGAUUUGGG | 518 | 453 | 29.0522 | 25.743 | 0.17446723 |
| miR5158 | UGAGACGGGAUGAGAUGAGAU | 424 | 377 | 23.7802 | 21.4241 | 0.15052625 |
| miR6263 | GAAUGGAAAAAGGGGAUGG | 197 | 176 | 11.0488 | 10.0017 | 0.14364445 |
| miR4403 | ACGACACGAACACGACCCGAUGAC | 1066 | 953 | 59.787 | 54.1569 | 0.14268666 |
| miR3694 | AUUAAGGGGUUGUGGGUGGCGGCU | 19 | 17 | 1.0656 | 0.9661 | 0.14142155 |
| miR6019 | AACAGGUGGACGGUUGUAAAUUUU | 1448 | 1299 | 81.2116 | 73.8194 | 0.1376858 |
| miR6300 | GUCGUUGUAGUAUAGUGGUGA | 4177 | 3749 | 234.2685 | 213.0476 | 0.13698718 |
| miR6466 | UCAGUGGUAGAGCAUUUGACUGCA | 1364 | 1227 | 76.5004 | 69.7278 | 0.13373333 |
| miR5265 | AAGUGAUGUGUGGAUGAUGGUUGA | 89 | 81 | 4.9916 | 4.6031 | 0.11689654 |
| miR7722 | GAGGGGACCGGGAUGAGAUGG | 905 | 824 | 50.7572 | 46.8261 | 0.1162996 |
| miR897 | UAACUUCAAGUGGAAUUCAGCAAA | 268 | 245 | 15.0309 | 13.9228 | 0.11048202 |
| miR5565 | UUUUGUUGGAAGAUUGUCGGA | 350 | 320 | 19.6299 | 18.1849 | 0.11031183 |
| miR5782 | UAGCUGUAGAGUAGAAGUUGAG | 393 | 360 | 22.0415 | 20.458 | 0.1075573 |
| miR3705 | GAGGUGGUUAUGAGCGGAC | 121 | 111 | 6.7863 | 6.3079 | 0.10546542 |
| miR6474 | UGUUCACACUCAGUAGCUGCA | 29 | 27 | 1.6265 | 1.5344 | 0.0840962 |
| miR7730 | AUGAACACGGCACGGUUGAAGUUA | 643 | 602 | 36.0629 | 34.2104 | 0.07608044 |
| miR6198 | GCUAUUCUUAGAGAUGGUCGAUUC | 55 | 52 | 3.0847 | 2.955 | 0.06197206 |
| miR1033 | AUAGACGGGUCGAUGAUGUGGCAU | 159 | 151 | 8.9176 | 8.581 | 0.0555097 |
| miR1168 | UUGUGGACAAGGCCAAGGA | 2001 | 1905 | 112.2268 | 108.257 | 0.05195692 |
| miR834 | UGAGUAGGAUCAGUAGGGUGGUAA | 129 | 123 | 7.235 | 6.9898 | 0.04974184 |
| miR5183 | UAUUAGAGUACAAAUUUGAGUCGA | 22 | 21 | 1.2339 | 1.1934 | 0.0481478 |
| miR7717 | UAUUGAGAUUCGUCGUUAAUAAAU | 25 | 24 | 1.4021 | 1.3639 | 0.03985138 |
| miR5524 | GAAAAAUGUGGAUUCAUGACGG | 431 | 417 | 24.1728 | 23.6972 | 0.02866799 |
| miR5780 | AUUAAACUUAACUGACGGUAGGGA | 1784 | 1728 | 100.0563 | 98.1985 | 0.02703912 |
| miR6257 | CUUAACUGUAAUGGAUUAGGGCAU | 33 | 32 | 1.8508 | 1.8185 | 0.02540008 |
| miR5148 | GAGGCCUAGAAAUGUCAUACUCA | 247 | 241 | 13.8531 | 13.6955 | 0.01650692 |
| miR6171 | AUUGUGGACGGCUGAAGGUUU | 889 | 868 | 49.8599 | 49.3266 | 0.01551414 |
| miR6478 | CCGACCUUAGCUCAGUUGGCA | 2380 | 2328 | 133.4831 | 132.2952 | 0.01289638 |
| miR5662 | AGAGUGUGAGCAAUUGGAGAG | 1505 | 1474 | 84.4085 | 83.7642 | 0.0110545 |
| miR1078 | CUUGAUUGAUUGUUGGAU | 1674 | 1642 | 93.8869 | 93.3113 | 0.00887207 |
| miR7122 | UUGGACAGAGAAAUCACGGUCG | 1652 | 1643 | 92.653 | 93.3681 | -0.01109204 |
| miR7508 | CAAGAAAAAGAAGGCUGGGAGAGU | 23 | 23 | 1.29 | 1.307 | -0.01888808 |
| miR8143 | GAUGAGAUGGGGACAAGAUAGGU | 29 | 29 | 1.6265 | 1.648 | -0.01894542 |
| miR3519 | UCAACAAGGACUGCAUUUCUA | 24 | 24 | 1.346 | 1.3639 | -0.01905946 |
| miR2199 | UGAUAACUCGACGGAUCGC | 10007 | 10057 | 561.2462 | 571.5176 | -0.02616416 |
| miR1850 | UGGAAAGUAGAAGAGAUUGGG | 1632 | 1653 | 91.5313 | 93.9364 | -0.03741913 |
| miR5631 | UGACGAGGAAGAGAUAAUUUU | 145 | 147 | 8.1324 | 8.3537 | -0.03873416 |
| miR399 | GGGCACCUCUUACUUGGCAUG | 71 | 72 | 3.9821 | 4.0916 | -0.03913566 |
| miR6116 | UCAUUGUACACAAGCUGAG | 3924 | 4003 | 220.0789 | 227.4819 | -0.04773093 |
| miR7532 | GAACAGCCUCUGGUCGAUGGA | 4278 | 4365 | 239.9332 | 248.0535 | -0.04801851 |
| miR447 | UUGGUGAAGACAGUCUUUUGUUGC | 186 | 190 | 10.4319 | 10.7973 | -0.04966865 |
| miR3954 | UUGGACAGAGAAAUCACGGUCA | 140892 | 144099 | 7901.9781 | 8188.8353 | -0.05144442 |
| miR6140 | AAGUUUGUAGAAGAGUUUGUGGCU | 704 | 723 | 39.4841 | 41.0865 | -0.05739263 |
| miR3950 | UAUUUUCUGCAACAUGAUUGU | 1294 | 1330 | 72.5745 | 75.581 | -0.05856088 |
| miR1873 | AUCAACAUUGGUAUCAGAGCUAAA | 27 | 28 | 1.5143 | 1.5912 | -0.07146413 |
| miR2665 | UCAUUUCAGGAAGAAUUGCA | 676 | 702 | 37.9137 | 39.8931 | -0.07341998 |
| miR7764 | CAAAACCUUAGAUCUGGAUCAA | 77 | 80 | 4.3186 | 4.5462 | -0.07409746 |
| miR5139 | AACCUGGCUCUGAUACCA | 1316 | 1370 | 73.8083 | 77.8541 | -0.07698996 |
| miR7504 | AGGGAAAAAUCUGAUCUGUGCAUU | 20 | 21 | 1.1217 | 1.1934 | -0.0893908 |
| miR3514 | AGGAUUCUGGAAUUAAACGGUGAA | 119 | 125 | 6.6742 | 7.1035 | -0.08993512 |
| miR5234 | UUUGUUAUGGAUGGCUGAAGG | 778 | 831 | 43.6344 | 47.2239 | -0.11405123 |
| miR5793 | CGAGGACGGACACGAGAGCAG | 84 | 90 | 4.7112 | 5.1145 | -0.11849863 |
| miR5207 | CAUUAAGGUGUUUGGACGUU | 845 | 910 | 47.3921 | 51.7133 | -0.12588878 |
| miR3633 | UUACCUAUGCCACCCAUUCCUU | 5823 | 6295 | 326.585 | 357.7313 | -0.13141782 |
| miR8022 | UUAAAUAGAUAUUUUGGACGACU | 364 | 394 | 20.4151 | 22.3902 | -0.13323078 |
| miR1887 | CACUAAGUAGAUCUAAGAGGAU | 24 | 26 | 1.346 | 1.4775 | -0.13447972 |
| miR169 | CAGCCAAGGAUGACUUGCCGG | 763 | 830 | 42.7931 | 47.1671 | -0.14040271 |
| miR858 | CUCGUUGUCUGUUCGACCUUG | 128 | 140 | 7.1789 | 7.9559 | -0.14826234 |
| miR827 | UUAGAUGACCAUCAACAAACA | 329 | 360 | 18.4521 | 20.458 | -0.1488801 |
| miR1863 | AGAGUUUGUGGCUGUAUCAUUACU | 987 | 1080 | 55.3562 | 61.3741 | -0.14888505 |
| miR5772 | UAGGAAUGUGAGUAGAGUAAGCAU | 243 | 267 | 13.6287 | 15.173 | -0.15485841 |
| miR5536 | AAGGUAGUGACUAUGUACGGUAGU | 87 | 96 | 4.8794 | 5.4555 | -0.16100767 |
| miR6035 | UGAAGUAAAAAUGCAUGACGU | 64 | 71 | 3.5895 | 4.0348 | -0.16871427 |
| miR162 | UCGAUAAACCUCUGCAUCCAG | 922 | 1023 | 51.7107 | 58.1349 | -0.16894168 |
| miR4376 | ACGCAGGAGAGAUGACGCUGU | 16 | 18 | 0.8974 | 1.0229 | -0.18884202 |
| miR6464 | UUGAUUGAUUGUUGGAUUUU | 11919 | 13424 | 668.4814 | 762.857 | -0.19052523 |
| miR7812 | CUGUUAGGAAAUUGAUGGGUGU | 669 | 755 | 37.5211 | 42.905 | -0.19344366 |
| miR1310 | GAGGCAUCGGGGGCGCAACGC | 684 | 772 | 38.3624 | 43.8711 | -0.1935779 |
| miR5741 | UAGAGACUGAAAUGGGAUGGUUU | 30 | 34 | 1.6826 | 1.9321 | -0.19947752 |
| miR7533 | AGGGGUGAGAGAGAAGCUG | 29 | 33 | 1.6265 | 1.8753 | -0.20535059 |
| miR2089 | UUACCUAUGCCACCCAUUCC | 2607 | 2976 | 146.2145 | 169.1197 | -0.20995833 |
| miR8142 | UGAGGUAGUAGACUGUAUAGUU | 2980 | 3402 | 167.1344 | 193.3283 | -0.21004414 |
| miR5562 | AUGAUGGAGAAUAGGCUGUCAAC | 28 | 32 | 1.5704 | 1.8185 | -0.21161685 |
| miR909 | UGUGGUCAAAGAGUGUGGUGG | 41 | 47 | 2.2995 | 2.6709 | -0.21600576 |
| miR5021 | UGGGCCAAGAAGAAGAAAGAAAA | 149 | 172 | 8.3567 | 9.7744 | -0.2260748 |
| miR6150 | AGAAUUUGUUUGAAUCUGUUGGCA | 18 | 21 | 1.0095 | 1.1934 | -0.24143677 |
| miR7748 | AAUUUCUUCUGAUUGUUGGACUGG | 40 | 47 | 2.2434 | 2.6709 | -0.25163908 |
| miR5532 | AUGGAAUAUAUGACAACGGUGGAG | 17 | 20 | 0.9535 | 1.1366 | -0.25341978 |
| miR1515 | UCAUUUUUGCGUGCAAUGAUCC | 204 | 240 | 11.4414 | 13.6387 | -0.25344254 |
| miR6118 | UGGACGAUAUGGGUGGUUCGGAAA | 365 | 430 | 20.4712 | 24.436 | -0.25541247 |
| miR168 | UCGCUUGGUGCAGGUCGGGAA | 55196 | 65311 | 3095.6873 | 3711.4832 | -0.26173609 |
| miR8030 | UUGGAUCGGUUUGGAUUCUCGGUU | 43 | 51 | 2.4117 | 2.8982 | -0.2651067 |
| miR473 | UGAGGCCGUUGGGGAGAGUGG | 648 | 769 | 36.3433 | 43.7006 | -0.26596366 |
| miR529 | GAAAGGAGAGAUGGAGCAG | 7535 | 8961 | 422.6032 | 509.2343 | -0.26902591 |
| miR1857 | UGGAUUUUUUUGAAGCAUCGG | 84 | 100 | 4.7112 | 5.6828 | -0.27050736 |
| miR156 | UUGACAGAAGAGAGUGAGCAC | 188175 | 224082 | 10553.862 | 12734.0966 | -0.27092559 |
| miR5770 | UUCAGGAUAUGGUUUUGAUAA | 48732 | 58373 | 2733.1516 | 3317.2117 | -0.2794056 |
| miR8135 | AUCGAUAUUUGCAGAGUUGAU | 20 | 24 | 1.1217 | 1.3639 | -0.28205099 |
| miR7696 | UUCAAAUGAGAACUUUGAAG | 12630 | 15168 | 708.3581 | 861.9647 | -0.28314991 |
| miR5671 | CAUGGUGGUGACGGGUGAC | 1636 | 1977 | 91.7556 | 112.3486 | -0.29211403 |
| miR5161 | UUUUGAUAGAGUGGAGUAUA | 10863 | 13155 | 609.2552 | 747.5703 | -0.29516259 |
| miR857 | GUGUUGAAUGUUGAAUGGUUGGCA | 23 | 28 | 1.29 | 1.5912 | -0.30274412 |
| miR5244 | UUAUCGGAUGAAGAUUGUUGG | 950 | 1158 | 53.2811 | 65.8066 | -0.30460842 |
| miR1028 | UGCACUUGUAGGGUUUAAGGAGGC | 17 | 21 | 0.9535 | 1.1934 | -0.32377284 |
| miR5272 | AAUUGUUAUGUUUGGAUAACAAUC | 17 | 21 | 0.9535 | 1.1934 | -0.32377284 |
| miR3515 | GAAUGUAGAGCAAAAUGAAGGUAU | 252 | 313 | 14.1335 | 17.7871 | -0.33171254 |
| miR2118 | GUCGAUGGAACAAUGUAGGCAAGG | 26575 | 33125 | 1490.4683 | 1882.4223 | -0.33682462 |
| miR165 | UCGGACCAGGCUUCAUCCCCC | 1441 | 1821 | 80.819 | 103.4835 | -0.35663435 |
| miR5020 | UGGCAUGAAGAUGAACUUGCA | 34 | 43 | 1.9069 | 2.4436 | -0.35777896 |
| miR5254 | AGCGGUGGAAGCAAUUGUGUA | 13056 | 16556 | 732.2504 | 940.8418 | -0.36161508 |
| miR2666 | CAAAGUGUUGAUAAUCAAGGA | 47 | 60 | 2.636 | 3.4097 | -0.37129444 |
| miR5230 | CAAAUCUUGGAUCGAACUGGA | 96 | 123 | 5.3842 | 6.9898 | -0.37651918 |
| miR472 | UUUUUCCCACACCUCCCAUCCC | 799 | 1024 | 44.8122 | 58.1917 | -0.37692184 |
| miR2108 | UUAUGAGUUUUGAUUUGUCGG | 66 | 86 | 3.7016 | 4.8872 | -0.40085914 |
| miR5070 | AACUAGUAGGUCAGAGACGU | 574 | 756 | 32.193 | 42.9618 | -0.41630742 |
| miR7984 | UCCGACUUUGUGAAAUGACUU | 740 | 983 | 41.5032 | 55.8618 | -0.42863949 |
| miR397 | UCAUUGAGUGCAGCGUUGAUG | 6566 | 8773 | 368.2565 | 498.5507 | -0.43702924 |
| miR6135 | CGUGUCGUGGUGUAGUUGGU | 47851 | 65531 | 2683.7404 | 3723.9854 | -0.47260229 |
| miR7496 | AGACCAAAUUGUUAGACGAUGUGU | 485 | 674 | 27.2014 | 38.302 | -0.49373882 |
| miR7535 | UGGAAAAUGACUGGGGGUGGU | 56 | 78 | 3.1408 | 4.4326 | -0.4970211 |
| miR166 | UCGGACCAGGCUUCAUUCCCC | 466222 | 650733 | 26148.2271 | 36979.7525 | -0.50002244 |
| miR6267 | UAGGAAUAGGUCAGGCAAUGU | 527 | 743 | 29.557 | 42.2231 | -0.51453266 |
| miR8122 | UAAGGAAGGUUUUGUGGACAAG | 1056 | 1492 | 59.2261 | 84.7871 | -0.51761169 |
| miR5541 | UCAAAGUGGUGUAUGUAAUGA | 36 | 51 | 2.0191 | 2.8982 | -0.52144479 |
| miR1869 | UGGAACAAUGUAGGCAAGGGAAGUA | 31379 | 44468 | 1759.9024 | 2527.0205 | -0.52194195 |
| miR5261 | UCUAUUGUAGAUGUUUUGGAU | 16 | 23 | 0.8974 | 1.307 | -0.54243605 |
| miR391 | UUGGCAGGGAGAUAGUGACCA | 115 | 167 | 6.4498 | 9.4902 | -0.55718407 |
| miR5822 | UGUCUGCGAGUCGGGUUG | 1411 | 2054 | 79.1364 | 116.7244 | -0.56069283 |
| miR1023 | AGACUGAGAAUUGAAGAGAGUGCA | 168 | 245 | 9.4223 | 13.9228 | -0.56329821 |
| miR1211 | AGGGAUGGGAAUGGUAUAUGCAAA | 17 | 25 | 0.9535 | 1.4207 | -0.5752971 |
| miR7545 | UUGAAGAAAUUAGAGUGCU | 372 | 548 | 20.8638 | 31.1417 | -0.57784576 |
| miR5072 | UUCGUUCCCCAGUGGAGUCGCCA | 80 | 118 | 4.4868 | 6.7057 | -0.57970106 |
| miR5059 | UCGUUCCUGGGCAGCAACACCA | 3059 | 4553 | 171.5651 | 258.7372 | -0.59273138 |
| miR167 | UGAAGCUGCCAGCAUGAUCUGA | 290089 | 438950 | 16269.745 | 24944.5815 | -0.61653483 |
| miR850 | UAAAGAUCCGGAAACAACCAAG | 492 | 746 | 27.594 | 42.3936 | -0.61949188 |
| miR780 | UUCUUCGGACACAUUUGGCAU | 32 | 49 | 1.7947 | 2.7846 | -0.6337274 |
| miR6480 | AUGCUGAAACGAUAGAAUAUU | 22 | 34 | 1.2339 | 1.9321 | -0.64694429 |
| miR6484 | UGAUGGGCUCUGCAAGAAUGG | 323 | 500 | 18.1156 | 28.4139 | -0.64936428 |
| miR2617 | GAGUGUGAGCAUGCCUGUU | 6204 | 9639 | 347.9536 | 547.7636 | -0.65465847 |
| miR6441 | AAUUGACGGAAGGGCUAC | 12172 | 19327 | 682.671 | 1098.3117 | -0.68602518 |
| miR2084 | CCUCAUUGAUGGAUUGUGUAA | 17 | 27 | 0.9535 | 1.5344 | -0.68636978 |
| miR5225 | UCUGUCGCAGGAGAGAUGGUGC | 17 | 27 | 0.9535 | 1.5344 | -0.68636978 |
| miR3440 | UGAGAUUGUGGGUCAAGGGAAGGU | 15 | 24 | 0.8413 | 1.3639 | -0.69704562 |
| miR3952 | UGAAGGGCCUUUCUAGAGCAC | 408 | 655 | 22.8828 | 37.2222 | -0.70189973 |
| miR8155 | AUCGUAACCUGGCUCUGAUACCA | 398 | 648 | 22.322 | 36.8244 | -0.72219572 |
| miR2088 | UCCAAGUGAAGAUCUAGGUCGA | 106 | 174 | 5.945 | 9.888 | -0.73400193 |
| miR398 | GGGGCGACAUGAGAUCACAUG | 7170 | 11820 | 402.132 | 671.7051 | -0.74015884 |
| miR2111 | UAAUCUGCAUCCUGAGGUUUG | 27 | 45 | 1.5143 | 2.5573 | -0.75597037 |
| miR3951 | UAGAUAAAGAUGAGAGAAAAA | 9318 | 15627 | 522.6034 | 888.0487 | -0.76492228 |
| miR535 | UGACAAUGAGAGAGAGCACAC | 78506 | 133618 | 4403.037 | 7593.2227 | -0.78621335 |
| miR4349 | UAUGGUGAGAGUAAGACAAAGAG | 12 | 21 | 0.673 | 1.1934 | -0.82639927 |
| miR157 | UUGACGGAAGAUAGAGAGCAC | 857469 | 1527938 | 48091.4546 | 86829.4201 | -0.85240339 |
| miR6203 | AGACGAUAUCAGAUAGACCUGCAU | 14 | 25 | 0.7852 | 1.4207 | -0.85546986 |
| miR5669 | AAUGUGAGUGUGGUUAAGUGGGGC | 17 | 31 | 0.9535 | 1.7617 | -0.88566342 |
| miR862 | CCAAUAGGUCGAACGGCAUGUGGC | 14 | 26 | 0.7852 | 1.4775 | -0.91202605 |
| miR5253 | GAAUGAAAAUUGAUUAGUGUGGAC | 10 | 19 | 0.5609 | 1.0797 | -0.94481502 |
| miR5301 | UGGGGUGGGGAUGGGGAAAGCAUU | 173 | 329 | 9.7028 | 18.6964 | -0.94628746 |
| miR172 | AGAAUCUUGAUGAUGCUGCAU | 27916 | 53143 | 1565.6788 | 3020.002 | -0.94776123 |
| miR5237 | UUCACAUGAUUAGUUGGAU | 193 | 368 | 10.8245 | 20.9126 | -0.95007205 |
| miR5052 | ACGGGUGGACGGUGGAUA | 3256 | 6263 | 182.6139 | 355.9128 | -0.96272724 |
| miR5369 | UGAGAAAGGAGAGAUGGUGCA | 1014 | 1998 | 56.8706 | 113.542 | -0.99747113 |
| miR1852 | AAAUAUGUGGAUUCGAAUGCAGGU | 11 | 22 | 0.6169 | 1.2502 | -1.01905036 |

* and ** indicates a significant difference at *P* < 0.05 and *P* < 0.01, respectively.
