# Supplementary material for: Boron-deficiency-responsive microRNAs and their targets in Citrus sinensis leaves
Source: BMC Plant Biol. 2015 Nov 4;15:271. doi: 10.1186/s12870-015-0642-y (PMC4634795; doi:10.1186/s12870-015-0642-y)
Supplement: Additional file 4: — List of novel miRNAs in Citrus sinensis leaves. (DOC 158 kb) [file 12870_2015_642_MOESM4_ESM.doc]

**Additional file 4: List of novel miRNAs in *Citrus sinensis* leaves.**

| miRNA | Location of miRNAs | MFE | MFEI | Sequence | miR* Sequence | Expressed | | Normalized read count | | Fold change |
| --- | --- | --- | --- | --- | --- | --- | --- | --- | --- | --- |
|  |  |  |  |  |  | Control | BD | Control | BD |  |
| ***Up-regulated miRNAs*** | | | | |  |  |  |  |  |  |
| novel_mir_95 | scaffold00120:72709:72835:+ 127(nt) | -72.90(kcal/mol) | 1.15 | GUUCUCAGGUCGCCCCUGUGGGA | CCGCAGGGGCGACATGAGATC | 0 | 35672 | 0.01 | 2000.6768 | 17.6101286** |
| novel_mir_127 | scaffold00330:77027:77129:+ 103(nt) | -53.10(kcal/mol) | 1.66 | AGGGACAAGCUAAAAGACCAA | TGTCTTTTAGCTTGTTCGTTCCTTAAAA | 0 | 1442 | 0.01 | 80.8751 | 12.98147988** |
| novel_mir_35 | scaffold00013:468461:468571:+ 111(nt) | -55.80(kcal/mol) | 1.17 | UUCAAUAAAGCUGUGGGAAG | TTTCCACAGCTTTCTTGAACT | 0 | 1242 | 0.01 | 69.658 | 12.76607334** |
| novel_mir_59 | scaffold00035:91768:91885:+ 118(nt) | -47.00(kcal/mol) | 0.09 | UGUUGGAACGGCUCAAUCAAA | TGATTGAGCCGTGCCAATATC | 0 | 649 | 0.01 | 36.3994 | 11.82969895** |
| novel_mir_132 | scaffold00396:76375:76481:+ 107(nt) | -58.40(kcal/mol) | 1.14 | CUGGAUGCAACUGUGACACGG | TTACTGTGCCACAGTTGCATCCAA | 0 | 448 | 0.01 | 25.1262 | 11.29497678** |
| novel_mir_167 | scaffold02238:8970:9143:- 174(nt) | -38.90(kcal/mol) | 0.60 | CUGAAAAGGAAUGCUGGUCAA | CCATTGGTAGTCTTTTAAGGT | 0 | 181 | 0.01 | 10.1514 | 9.98746299** |
| novel_mir_71 | scaffold00061:256186:256346:+ 161(nt) | -67.70(kcal/mol) | 0.89 | AGGUGCAGUUGCAAGUGCAGA | ATTCTGCATTTGCACCTGCATCTTG | 0 | 155 | 0.01 | 8.6932 | 9.76374353** |
| novel_mir_190 | scaffold09175:570:695:- 126(nt) | -41.70(kcal/mol) | 0.83 | AGCUGCCGACUCAUUCAUUCA | TGTGAATGAAGCGGGAGATAAT | 0 | 109 | 0.01 | 6.1133 | 9.25580756** |
| novel_mir_11 | scaffold00003:1499741:1499916:+ 176(nt) | -66.00(kcal/mol) | 1.17 | UACUAGGGAUCUCAACAUUUGGA | CAAATATTGGGATTTCTAGCATT | 0 | 102 | 0.01 | 5.7207 | 9.16004788** |
| novel_mir_14 | scaffold00004:3424292:3424394:- 103(nt) | -56.30(kcal/mol) | 1.71 | UUGUGGAGUGUGUAUGUUACA | ATGTAACATACACATTTCACAATA | 0 | 94 | 0.01 | 5.272 | 9.04220656** |
| novel_mir_29 | scaffold00010:938702:938805:+ 104(nt) | -62.90(kcal/mol) | 1.34 | GUUUAGGACAGCUGCUGCCAAA | TGGCGCAGCTGTCCTAAACGG | 0 | 39 | 0.01 | 2.1873 | 7.7730073** |
| novel_mir_16 | scaffold00005:358662:358762:+ 101(nt) scaffold00346:55913:56034:- 122(nt) | -50.76(kcal/mol) -42.22(kcal/mol) | 1.09 0.74 | UCUCUCGUCUUUCUCUCGUGC | AGCACGAGAGAAAGACAAGAGAAT | 0 | 30 | 0.01 | 1.6826 | 7.39454844** |
| novel_mir_102 | scaffold00140:240485:240692:- 208(nt) | -58.40(kcal/mol) | 0.62 | AAGGCUUUCAAGAAGGACCCA | AGCATGGGTTCTCTCCAACGCC | 0 | 28 | 0.01 | 1.5704 | 7.29498827** |
| novel_mir_78 | scaffold00078:95415:95752:+ 338(nt) | -66.30(kcal/mol) | 0.68 | AGGGAUCAGGCUACGGUGCAA | ATTCGTATCGTGTTTGTGTCGGAT | 0 | 26 | 0.01 | 1.4582 | 7.1880448** |
| novel_mir_140 | scaffold00614:94804:94872:+ 69(nt) | -23.60(kcal/mol) | 0.88 | ACAAAGGAUGUGGCACGGUAC | TGGTACCGTAGCCTGATCCTTAAG | 0 | 26 | 0.01 | 1.4582 | 7.1880448** |
| novel_mir_184 | scaffold05376:3349:3456:- 108(nt) | -61.00(kcal/mol) | 1.28 | UGCAUGUAAGGUACAGCCUCU | AGAGACTGTACCTTACATGCAAC | 0 | 25 | 0.01 | 1.4021 | 7.13144544** |
| novel_mir_68 | scaffold00051:232402:232504:+ 103(nt) | -64.60(kcal/mol) | 1.90 | UGAGAGGCAUGUAUCUUACAU | ATTGTAAGATACATGCCTCTCACA | 0 | 24 | 0.01 | 1.346 | 7.0725346** |
| novel_mir_188 | scaffold06001:2936:3069:- 134(nt) | -38.40(kcal/mol) | 0.77 | UUGGGCAGAGAAACACGGUA | ACCGTGATTTCTCTGTCCAAGCAGT | 0 | 22 | 0.01 | 1.2339 | 6.94708167** |
| novel_mir_103 | scaffold00140:253896:254002:- 107(nt) | -42.90(kcal/mol) | 1.06 | CUGAAGUGGCACUCAUUUAUU | CAATAGATGAGTGACACATAG | 0 | 20 | 0.01 | 1.1217 | 6.80954307** |
| novel_mir_163 | scaffold01224:25045:25140:- 96(nt) | -18.25(kcal/mol) | 0.48 | AAGACUAAGUUCUUCUCGGUACA | ATGTGACCGAGAACAATACCTTGT | 0 | 18 | 0.01 | 1.0095 | 6.6574971** |
| novel_mir_84 | scaffold00087:148005:148112:+ 108(nt) | -50.50(kcal/mol) | 0.95 | GGAAACCCUAGGGGGAGGUCG | AACCTCCCTCGAAGGCTTCCAATA | 7 | 386 | 0.3978 | 21.6489 | 5.76610664** |
| novel_mir_7 | scaffold00001:5073938:5074113:- 176(nt) scaffold00011:255388:255563:- 176(nt) scaffold00095:405526:405702:+ 177(nt) scaffold00184:55106:55281:- 176(nt) scaffold00244:120792:120949:- 158(nt) scaffold00792:63186:63361:+ 176(nt) | -69.50(kcal/mol) -67.40(kcal/mol) -64.40(kcal/mol) -64.90(kcal/mol) -57.80(kcal/mol) -67.40(kcal/mol) | 0.96 0.91 0.91 0.95 0.8 0.93 | AUACAGAGUCUGUAACGUAGU | TACGTTACAGATTCTGTATGT | 7 | 28 | 0.3978 | 1.5704 | 1.9810169** |
| novel_mir_104 | scaffold00144:71460:71597:- 138(nt) | -38.59(kcal/mol) | 0.87 | GAAUUAGAUCAAUGGCUUAUA | TGAGTCATGGATCTAATGCAA | 16 | 57 | 0.9092 | 3.1969 | 1.81400403** |
| novel_mir_52 | scaffold00022:586422:586564:+ 143(nt) scaffold00022:586421:586563:- 143(nt) | -74.90(kcal/mol) -74.80(kcal/mol) | 1.54 1.54 | UUGUGGUAGAUUGUUUGCUUA | TAAGCAAACAATCTACCACAAAG | 337 | 971 | 19.151 | 54.4589 | 1.50774812** |
| novel_mir_94 | scaffold00118:395167:395296:- 130(nt) | -45.40(kcal/mol) | 1.03 | ACUGAUGUGGCAUGAAGAGAU | TTATCATGCCACATCAGTTTGTAC | 11 | 25 | 0.6251 | 1.4021 | 1.16543034* |
| novel_mir_89 | scaffold00102:303764:303898:+ 135(nt) | -58.72(kcal/mol) | 1.32 | UCCCUACUCCACCCAUGCCAUA | TGGTATGGGTGAGTAGGGAAG | 3693 | 7785 | 209.8652 | 436.6245 | 1.05693013** |
|  |  |  |  |  |  |  |  |  |  |  |
| ***Down-regulated miRNAs*** | | | | |  |  |  |  |  |  |
| novel_mir_236 | scaffold00120:72699:72845:+ 147(nt) | -80.30(kcal/mol) | 1.14 | CCGCAGGGGCGACAUGAGAUC | TCTCAGGTCGCCCCTGTGGGA | 64485 | 0 | 3664.5434 | 0.01 | -18.48327393** |
| novel_mir_237 | scaffold00120:328021:328212:- 192(nt) | -63.20(kcal/mol) | 1.18 | UAGAUAACGGAUUAACGGCUA | TAGCCGTTGATCCGTTATCTA | 2237 | 0 | 127.1239 | 0.01 | -13.63394767** |
| novel_mir_211 | scaffold00015:714697:714778:+ 82(nt) | -52.40(kcal/mol) | 1.07 | UCGCAGGGGAGAUGGGACCAAC | AGCAGCCGGTGCCATCCTCCTGAA | 393 | 0 | 22.3333 | 0.01 | -11.12498073** |
| novel_mir_224 | scaffold00054:120262:120464:+ 203(nt) | -89.24(kcal/mol) | 1.22 | AGCAGGAAAGUGGCUGGUUGA | TCAACGAGTCACTTTTCTGTTAAC | 213 | 0 | 12.1043 | 0.01 | -10.24130393** |
| novel_mir_259 | scaffold00346:128985:129107:+ 123(nt) | -53.10(kcal/mol) | 1.17 | GGAAUGGCGGCUGGUUCAAAG | CTTTGGACCAGGCTTCATTCC | 114 | 0 | 6.4784 | 0.01 | -9.33949374** |
| novel_mir_239 | scaffold00124:390114:390333:+ 220(nt) | -63.10(kcal/mol) | 0.82 | UCACUACUUUCAAUCUCGGUC | ATTGAGATTGAAAGTAGTGAT | 96 | 0 | 5.4555 | 0.01 | -9.09156762** |
| novel_mir_315 | scaffold12451:533:628:+ 96(nt) | -53.60(kcal/mol) | 2.07 | AUGGCAUGAUAAGAUUGGUUGAA | ATTCAACTAATCTTATCATGCCAC | 75 | 0 | 4.2621 | 0.01 | -8.73542063** |
| novel_mir_285 | scaffold01507:20306:20407:- 102(nt) | -20.10(kcal/mol) | 0.53 | UCCGACAAUGUGGUAAACGUGUU | AAATGTGTTATACCAGGCTTGATG | 73 | 0 | 4.1484 | 0.01 | -8.6964112** |
| novel_mir_267 | scaffold00624:80084:80212:- 129(nt) | -30.50(kcal/mol) | 0.88 | CAAUUGGAUUGGAUAUAUGGAU | ATCCATCCAATATCTAATTGGATA | 65 | 0 | 3.6938 | 0.01 | -8.52896194** |
| novel_mir_262 | scaffold00439:112609:112756:- 148(nt) | -33.90(kcal/mol) | 0.72 | CAAGUAUUGAAGAAAGAACAC | TTTTTCTTCGATCCATAAACG | 59 | 0 | 3.3528 | 0.01 | -8.38922262** |
| novel_mir_301 | scaffold04567:3526:3599:- 74(nt) | -28.10(kcal/mol) | 0.77 | UCUCAUCGGUUUGGAUGGCAUU | GCTGCAATTTGGTGAGAAGATGAT | 44 | 0 | 2.5004 | 0.01 | -7.9660151** |
| novel_mir_207 | scaffold00010:938695:938815:+ 121(nt) | -66.00(kcal/mol) | 1.24 | UGGCGCAGCUGUCCUAAACGG | TGTTTAGGACAGCTGCTGCCAA | 40 | 0 | 2.2731 | 0.01 | -7.82851734** |
| novel_mir_219 | scaffold00036:835878:835996:+ 119(nt) | -44.10(kcal/mol) | 1.09 | AGCUUGAGUCUUGCUGAAAGUA | CTTTCAGCAGCCTCCGGCGTC | 39 | 0 | 2.2163 | 0.01 | -7.79200937** |
| novel_mir_300 | scaffold04341:5380:5481:- 102(nt) | -18.60(kcal/mol) | 0.54 | AGAGAUGUGUUGUACAAAUUCUG | ACTAAATATGTTGACACACTCTGG | 20 | 0 | 1.1366 | 0.01 | -6.82858081** |
| novel_mir_202 | scaffold00002:4781850:4782058:+ 209(nt) | -87.20(kcal/mol) | 1.74 | CUAGAAAUUGGAGGAAAAUAA | ACATTATTTTCCTCCAATTTCTAG | 19 | 0 | 1.0797 | 0.01 | -6.7544867** |
| novel_mir_201 | scaffold00001:5038722:5038836:+ 115(nt) | -44.09(kcal/mol) | 0.72 | CACGCGCUCCCCUUCUCCAAC | TGGAGAAGCAGGGCACGTGCAA | 18 | 0 | 1.0229 | 0.01 | -6.6765213** |
| novel_mir_208 | scaffold00011:1667296:1667391:- 96(nt) | -34.72(kcal/mol) | 0.84 | CAACGGCUGGAUGCAACUGUA | CAGTTACATCCAGCCGTTGG | 18 | 0 | 1.0229 | 0.01 | -6.6765213** |
| novel_mir_134 | scaffold00445:44117:44243:+ 127(nt) scaffold00445:44127:44228:+ 102(nt) | -67.40(kcal/mol) -56.10(kcal/mol) | 1.11 1.2 | GUGACAGAAGAUAGAGAGCGC | GCTCTCTGTGCTTCTGTCATCA | 31929 | 2749 | 1814.4562 | 154.1786 | -3.55686279** |
| novel_mir_121 | scaffold00270:89897:90142:- 246(nt) | -93.30(kcal/mol) | 0.95 | CUGACAGCGGCUGUACUGUAGU | TACACTACAGCTGCTGTCAATC | 7288 | 2559 | 414.1613 | 143.5224 | -1.52891683** |
| novel_mir_111 | scaffold00180:294564:294666:- 103(nt) | -37.70(kcal/mol) | 1.14 | UGCUUGUUGAUUGUCAUCUAA | TTAGATGACCATCAACAAACA | 3904 | 1570 | 221.8559 | 88.054 | -1.33316247** |
| novel_mir_187 | scaffold05694:2312:2452:- 141(nt) | -47.17(kcal/mol) | 1.01 | CUGAUGAGAGAGCGAAUGAUA | TCATTCGCGCTCTCATCATTA | 1264 | 512 | 71.8304 | 28.7157 | -1.32275482** |
| novel_mir_125 | scaffold00307:187446:187562:- 117(nt)  scaffold00307:198728:198868:- 141(nt) scaffold00337:36062:36183:- 122(nt) scaffold04340:5890:6010:- 121(nt) | -57.40(kcal/mol) -57.70(kcal/mol) -45.30(kcal/mol) -52.30(kcal/mol) | 0.96 0.93 0.96 0.88 | AGGCAGUCUCCUUGGCUAAG | TAGCCAAGGATGACTTGCCTGC | 26 | 11 | 1.4775 | 0.6169 | -1.26004958* |
| novel_mir_106 | scaffold00152:151005:151135:+ 131(nt) | -53.87(kcal/mol) | 1.01 | UUUCUCUUAUCGUUAUCUGU | CATAGATAAAGATGAGAGAAAAA | 30878 | 13876 | 1754.7301 | 778.2404 | -1.17296136** |
| novel_mir_99 | scaffold00124:390243:390458:+ 216(nt) scaffold00718:62265:62457:+ 193(nt) | -47.50(kcal/mol) -49.40(kcal/mol) | 0.63 0.73 | UUCCACCAAAGCAUUCAUUUCC | TGTCTTGGAATTTGAATGATGGTT | 469 | 229 | 26.6523 | 12.8435 | -1.05322163** |
|  |  |  |  |  |  |  |  |  |  |  |
| ***Equally expressed miRNAs*** | | | | |  |  |  |  |  |  |
| novel_mir_182 | scaffold05298:4054:4203:- 150(nt) scaffold05298:4064:4197:- 134(nt) | -49.70(kcal/mol) -46.60(kcal/mol) | 0.97 1.02 | GCUCAAGAAUGCCGUGGGAAA | TTCCACGGCTTTCTTGAACTT | 432 | 221 | 24.5496 | 12.3949 | -0.985953 |
| novel_mir_41 | scaffold00015:695981:696073:+ 93(nt) | -54.41(kcal/mol) | 1.30 | UAGCUUACGCCACACACAGCA | TGCTGTGTGTGGATGTACGTTAC | 18 | 10 | 1.0229 | 0.5609 | -0.866850 |
| novel_mir_10 | scaffold00003:1252097:1252193:+ 97(nt) | -64.20(kcal/mol) | 1.30 | AUGGGGAGUAGCUGCGCGGUG | CATAGCGCAGCTGCTCCCCATGC | 2327 | 1426 | 132.2384 | 79.9777 | -0.725471 |
| novel_mir_24 | scaffold00008:148398:148540:- 143(nt) scaffold00008:148409:148530:- 122(nt) | -57.10(kcal/mol) -44.50(kcal/mol) | 1.02 0.96 | GGUCAUGGGAGGAUUGGCGAGA | TTGCCAACTCCTCCCATGCCGA | 75153 | 53188 | 4270.7829 | 2983.0679 | -0.517704 |
| novel_mir_88 | scaffold00097:473108:473192:- 85(nt) | -23.70(kcal/mol) | 0.77 | UAGGCAUAUUGUCGAACCACAU | TTTCTGTAATTATTTAGTGTGCTT | 22 | 16 | 1.2502 | 0.8974 | -0.478336 |
| novel_mir_30 | scaffold00010:1490210:1490324:+ 115(nt) | -42.40(kcal/mol) | 0.92 | ACCAGCGCUGCACUCGAUCAU | CATCATTGAGTGCAGCGTTGA | 108 | 88 | 6.1374 | 4.9355 | -0.314431 |
| novel_mir_175 | scaffold03620:2856:3012:- 157(nt) | -61.30(kcal/mol) | 0.80 | GCUGUAGAAAGGCCCCUCAAC | AGTTGAAGGGCCTTTCTAGAGC | 9479 | 7771 | 538.6711 | 435.8393 | -0.305608 |
| novel_mir_146 | scaffold00708:8740:8865:+ 126(nt) | -51.10(kcal/mol) | 0.97 | AGCAAGCAUCCUGGGCUAAU | TAGCCAAGGATGACTTGCCTA | 140 | 118 | 7.9559 | 6.6181 | -0.265608 |
| novel_mir_191 | scaffold09523:450:585:- 136(nt) | -70.30(kcal/mol) | 1.12 | UGAGGGAAGAGCUUAGAAGG | TTCTAAACTCTCTCCCTCATGG | 1063 | 952 | 60.408 | 53.3933 | -0.178081 |
| novel_mir_26 | scaffold00008:1773769:1774113:- 345(nt) | -92.97(kcal/mol) | 0.87 | AGGUCAUCUUGCAGCUUCAAU | TTGAAGCTGCCAGCATGATCTG | 60 | 55 | 3.4097 | 3.0847 | -0.144515 |
| novel_mir_8 | scaffold00002:4214950:4215262:- 313(nt) scaffold03354:14:169:+ 156(nt) scaffold00002:4214950:4215262:+ 313(nt) | -82.40(kcal/mol) -27.32(kcal/mol) -107.30(kcal/mol) | 1.88 1.03 2.45 | UAAUAUAGGAAUAAAUUGGACA | TTGTCCAATTTATTCCTATAT | 356 | 334 | 20.2307 | 18.7325 | -0.111003 |
| novel_mir_160 | scaffold01078:7509:7590:- 82(nt) scaffold05314:439:520:+ 82(nt) | -30.92(kcal/mol) -31.92(kcal/mol) | 0.79 0.79 | CAGCUUGUCUACAAUGAUAAG | TAGGTATGGGCGGGCTGGGCATT | 65 | 67 | 3.6938 | 3.7577 | 0.024744 |
| novel_mir_82 | scaffold00084:407529:407741:+ 213(nt) | -87.90(kcal/mol) | 1.21 | UUGAGAAGUGUAGUAUUAUUU | ATAATACTACACTTCCGAAGT | 101 | 120 | 5.7396 | 6.7302 | 0.229699 |
| novel_mir_138 | scaffold00534:47809:47932:+ 124(nt) | -48.40(kcal/mol) | 1.08 | CAAGAAACAAAUAAGGAGGAGGU | ACCTCCTTGTTGGTTTCTTGAGGA | 15 | 18 | 0.8524 | 1.0095 | 0.244038 |
| novel_mir_168 | scaffold02299:12331:12515:+ 185(nt) | -43.01(kcal/mol) | 0.65 | UAAUCGUGGGAGACGAAGCUG | CAACCGGCGTCGGCTGTCATGGCT | 11985 | 14651 | 681.0817 | 821.7066 | 0.270795 |
| novel_mir_128 | scaffold00337:108014:108121:+ 108(nt) | -60.10(kcal/mol) | 1.26 | CUGGAGACAACUGUGGUACGG | GTATCACAGTTGCATCCAGCC | 308 | 386 | 17.503 | 21.6489 | 0.306692 |
| novel_mir_40 | scaffold00014:1373904:1374000:+ 97(nt) | -38.40(kcal/mol) | 0.72 | UGCAGGUGAGAUGAUACCGUCA | CCGGAATCATTTCTCCCGCGT | 26 | 33 | 1.4775 | 1.8508 | 0.324991 |
| novel_mir_90 | scaffold00107:257840:258025:- 186(nt) | -59.32(kcal/mol) | 0.94 | UCCCGUGAACGUAGGUAUAUU | CTACGTTCATGGGAGAAAGAGAA | 17 | 24 | 0.9661 | 1.346 | 0.478434 |
| novel_mir_22 | scaffold00008:142912:143016:- 105(nt) | -42.00(kcal/mol) | 1.08 | AAUGGGAGGCUUGGCAAGAAG | TCTTGCCCACCCCTCCCATTCC | 3513 | 5069 | 199.6362 | 284.2967 | 0.510024 |
| novel_mir_77 | scaffold00077:118399:118540:+ 142(nt) scaffold00248:131470:131612:+ 143(nt)  scaffold01173:21170:21312:+ 143(nt) | -86.00(kcal/mol) -67.40(kcal/mol)  -38.10(kcal/mol) | 1.48 1.63 0.99 | UAUGUUGCAACUGUGGUAUGGUA | TGCGGTACCGTACCACAGTTGCAT | 86 | 146 | 4.8872 | 8.1885 | 0.744591 |
| novel_mir_176 | scaffold03643:1001:1084:+ 84(nt) | -18.10(kcal/mol) | 0.57 | GAUCAAGUGGUUGAGACACAG | TTTGTTTCAATGCAGTTGCAAAA | 26 | 49 | 1.4775 | 2.7482 | 0.895329 |

* and ** indicates a significant difference at *P* < 0.05 and *P* < 0.01, respectively.
