# Supplementary material for: Boron-deficiency-responsive microRNAs and their targets in Citrus sinensis leaves
Source: BMC Plant Biol. 2015 Nov 4;15:271. doi: 10.1186/s12870-015-0642-y (PMC4634795; doi:10.1186/s12870-015-0642-y)
Supplement: Additional file 5: — List of novel miRNAs in Citrus sinensis leaves after removing these miRNAs with normalized read-count less than 10 TPM in two miRNA libraries constructed from control and B-deficient leaves. (DOC 69 kb) [file 12870_2015_642_MOESM5_ESM.doc]

**Additional file 5: List of novel miRNAs in *Citrus sinensis* leaves after removing these miRNAs with normalized read-count less than 10 TPM in two miRNA libraries constructed from control and B-deficient leaves.**

| miRNA | Sequence | Expressed | | Normalized read count | | Fold change |
| --- | --- | --- | --- | --- | --- | --- |
|  |  | Control | B-deficiency | Control | B-deficiency |  |
| ***Up-regulated miRNAs*** | |  |  |  |  |  |
| novel_mir_95 | GTTCTCAGGTCGCCCCTGTGGGA | 0 | 35672 | 0.01 | 2000.6768 | 17.6101286** |
| novel_mir_127 | AGGGACAAGCTAAAAGACCAA | 0 | 1442 | 0.01 | 80.8751 | 12.98147988** |
| novel_mir_35 | TTCAATAAAGCTGTGGGAAG | 0 | 1242 | 0.01 | 69.658 | 12.76607334** |
| novel_mir_59 | TGTTGGAACGGCTCAATCAAA | 0 | 649 | 0.01 | 36.3994 | 11.82969895** |
| novel_mir_132 | CTGGATGCAACTGTGACACGG | 0 | 448 | 0.01 | 25.1262 | 11.29497678** |
| novel_mir_167 | CTGAAAAGGAATGCTGGTCAA | 0 | 181 | 0.01 | 10.1514 | 9.98746299** |
| novel_mir_84 | GGAAACCCTAGGGGGAGGTCG | 7 | 386 | 0.3978 | 21.6489 | 5.76610664** |
| novel_mir_52 | TTGTGGTAGATTGTTTGCTTA | 337 | 971 | 19.151 | 54.4589 | 1.50774812** |
|  |  |  |  |  |  |  |
| ***Down-regulated miRNAs*** | |  |  |  |  |  |
| novel_mir_236 | CCGCAGGGGCGACATGAGATC | 64485 | 0 | 3664.5434 | 0.01 | -18.48327393** |
| novel_mir_237 | TAGATAACGGATTAACGGCTA | 2237 | 0 | 127.1239 | 0.01 | -13.63394767** |
| novel_mir_211 | TCGCAGGGGAGATGGGACCAAC | 393 | 0 | 22.3333 | 0.01 | -11.12498073** |
| novel_mir_224 | AGCAGGAAAGTGGCTGGTTGA | 213 | 0 | 12.1043 | 0.01 | -10.24130393** |
| novel_mir_134 | GTGACAGAAGATAGAGAGCGC | 31929 | 2749 | 1814.4562 | 154.1786 | -3.55686279** |
| novel_mir_121 | CTGACAGCGGCTGTACTGTAGT | 7288 | 2559 | 414.1613 | 143.5224 | -1.52891683** |
|  |  |  |  |  |  |  |
| ***Equally expressed miRNAs*** | |  |  |  |  |  |
| novel_mir_111 | TGCTTGTTGATTGTCATCTAA | 3904 | 1570 | 221.8559 | 88.054 | -1.33316247** |
| novel_mir_187 | CTGATGAGAGAGCGAATGATA | 1264 | 512 | 71.8304 | 28.7157 | -1.32275482** |
| novel_mir_106 | TTTCTCTTATCGTTATCTGT | 30878 | 13876 | 1754.7301 | 778.2404 | -1.17296136** |
| novel_mir_99 | TTCCACCAAAGCATTCATTTCC | 469 | 229 | 26.6523 | 12.8435 | -1.05322163** |
| novel_mir_89 | TCCCTACTCCACCCATGCCATA | 3693 | 7785 | 209.8652 | 436.6245 | 1.05693013** |
| novel_mir_182 | GCTCAAGAATGCCGTGGGAAA | 432 | 221 | 24.5496 | 12.3949 | -0.985953 |
| novel_mir_10 | ATGGGGAGTAGCTGCGCGGTG | 2327 | 1426 | 132.2384 | 79.9777 | -0.725471 |
| novel_mir_24 | GGTCATGGGAGGATTGGCGAGA | 75153 | 53188 | 4270.7829 | 2983.0679 | -0.517704 |
| novel_mir_175 | GCTGTAGAAAGGCCCCTCAAC | 9479 | 7771 | 538.6711 | 435.8393 | -0.305608 |
| novel_mir_191 | TGAGGGAAGAGCTTAGAAGG | 1063 | 952 | 60.408 | 53.3933 | -0.178081 |
| novel_mir_8 | TAATATAGGAATAAATTGGACA | 356 | 334 | 20.2307 | 18.7325 | -0.111003 |
| novel_mir_168 | TAATCGTGGGAGACGAAGCTG | 11985 | 14651 | 681.0817 | 821.7066 | 0.270795 |
| novel_mir_128 | CTGGAGACAACTGTGGTACGG | 308 | 386 | 17.503 | 21.6489 | 0.306692 |
| novel_mir_22 | AATGGGAGGCTTGGCAAGAAG | 3513 | 5069 | 199.6362 | 284.2967 | 0.510024 |

A 1.5 log2-fold cut-off was set to determine up-regulated and down-regulated miRNAs in addition a P-value of less than 0.01. * and ** indicate a significant difference at *P* < 0.05 and *P* < 0.01, respectively.
