# Supplementary material for: Boron-deficiency-responsive microRNAs and their targets in Citrus sinensis leaves
Source: BMC Plant Biol. 2015 Nov 4;15:271. doi: 10.1186/s12870-015-0642-y (PMC4634795; doi:10.1186/s12870-015-0642-y)
Supplement: Additional file 6: — List of target genes for parts of known miRNAs in Citrus sinensis leaves. (DOC 198 kb) [file 12870_2015_642_MOESM6_ESM.doc]

**Additional file 6: List of target genes for parts of known miRNAs in *Citrus sinensis* leaves.**

| *miRNA* | *Assession* | *Homology* | *Target genes* |
| --- | --- | --- | --- |
| miR1026 | orange1.1g002027m  orange1.1g040741m | AT2G40840.1  AT5G65720.1 | Disproportionating enzyme 2  Nitrogen fixation S (NIFS)-like 1 |
| miR1159 | orange1.1g045110m  orange1.1g028965m | AT5G09690.2  AT1G16180.2 | Magnesium transporter 7  Serinc-domain containing serine and sphingolipid biosynthesis protein |
| miR1446 | orange1.1g037028m | AT1G14920.1 | GRAS family transcription factor family protein |
| miR1516 | orange1.1g034867m  orange1.1g023374m  orange1.1g032745m  orange1.1g046265m  orange1.1g048542m | AT2G26100.1  AT4G20330.1  AT5G53560.1  AT4G08850.1  AT5G38280.1 | Galactosyltransferase family protein  Transcription initiation factor TFIIE, beta subunit  Cytochrome B5 isoform E  Leucine-rich repeat receptor-like protein kinase family protein  PR5-like receptor kinase |
| miR1535 | orange1.1g001616m  orange1.1g015157m | AT3G63380.1  AT3G58060.1 | ATPase E1-E2 type family protein / haloacid dehalogenase-like hydrolase family protein  Cation efflux family protein |
| miR158 | orange1.1g022993m | AT1G69840.1 | SPFH/Band 7/PHB domain-containing membrane-associated protein family |
| miR159 | orange1.1g039708m  orange1.1g044979m  orange1.1g046419m  orange1.1g011938m  orange1.1g038795m | AT5G06100.2  AT4G27330.1  AT4G26930.1  AT3G11440.1  AT3G60460.1 | Myb domain protein 33  Sporocyteless (SPL)  Myb domain protein 97  Myb domain protein 65  Myb-like HTH transcriptional regulator family protein |
| miR164 | orange1.1g030909m  orange1.1g047710m  orange1.1g017636m  orange1.1g017827m | AT1G56010.2  AT5G53950.1  AT3G08030.1  AT5G61430.1 | NAC domain containing protein 1  NAC (No Apical Meristem) domain transcriptional regulator superfamily protein  Protein of unknown function, DUF642  NAC domain containing protein 100 |
| miR2099 | orange1.1g006412m  orange1.1g042571m  orange1.1g017694m  orange1.1g002431m  orange1.1g043065m  orange1.1g016767m  orange1.1g031725m | AT1G43620.2  AT5G42400.1  AT3G22830.1  AT3G04240.1  AT2G16570.1  AT5G65000.1  AT2G33450.1 | UDP-Glycosyltransferase superfamily protein  SET domain protein 25  Heat shock transcription factor A6B  Tetratricopeptide repeat (TPR)-like superfamily protein  GLN phosphoribosyl pyrophosphate amidotransferase 1  Nucleotide-sugar transporter family protein  Ribosomal L28 family |
| miR2614 | orange1.1g014044m | AT5G24670.1 | Cytidine/deoxycytidylate deaminase family protein |
| miR2630 | orange1.1g032710m  orange1.1g005928m  orange1.1g016130m  orange1.1g023739m  orange1.1g019814m  orange1.1g009434m | AT1G80245.3  AT3G09070.1  AT1G69690.1  AT2G41870.1  AT1G08970.4  AT5G62810.1 | Spc97 / Spc98 family of spindle pole body (SBP) component  Protein of unknown function (DUF740)  TCP family transcription factor  Remorin family protein  Nuclear factor Y, subunit C9  Peroxin 14 |
| miR2643 | orange1.1g018307m  orange1.1g005129m  orange1.1g020050m  orange1.1g036061m  orange1.1g000172m  orange1.1g022592m  orange1.1g020718m  orange1.1g000410m | AT1G12500.1  AT4G34830.1  AT5G19890.1  AT1G56430.1  AT3G48190.1  AT5G40530.1  AT1G74070.1  AT4G29380.1 | Nucleotide-sugar transporter family protein  Pentatricopeptide repeat (PPR) superfamily protein  Peroxidase superfamily protein  Nicotianamine synthase 4  Ataxia-telangiectasia mutated  S-adenosyl-L-methionine-dependent methyltransferases superfamily protein  Cyclophilin-like peptidyl-prolyl cis-trans isomerase family protein  Protein kinase family protein / WD-40 repeat family protein |
| miR2648 | orange1.1g039062m  orange1.1g003798m  orange1.1g045701m  orange1.1g024671m  orange1.1g028115m  orange1.1g038710m | AT5G53480.1  AT5G58460.1  AT4G27110.1  AT2G44040.1  AT3G59890.1  AT4G16120.1 | ARM repeat superfamily protein  Cation/H+ exchanger 25  COBRA-like protein 11 precursor  Dihydrodipicolinate reductase, bacterial/plant  Dihydrodipicolinate reductase, bacterial/plant  COBRA-like protein-7 precursor |
| miR2663 | orange1.1g024446m  orange1.1g041969m | AT2G27680.1  AT4G15390.1 | NAD(P)-linked oxidoreductase superfamily protein  HXXXD-type acyl-transferase family protein |
| miR2864 | orange1.1g044947m  orange1.1g005621m | AT2G03360.2  AT3G55550.1 | Glycosyltransferase family 61 protein  Concanavalin A-like lectin protein kinase family protein |
| miR2868 | orange1.1g011851m | AT5G15080.1 | Protein kinase superfamily protein |
| miR2928 | orange1.1g047553m  orange1.1g009101m  orange1.1g017875m  orange1.1g010495m  orange1.1g012793m  orange1.1g012814m  orange1.1g012807m  orange1.1g012801m  orange1.1g026210m  orange1.1g031188m  orange1.1g002431m  orange1.1g047144m  orange1.1g027531m  orange1.1g013125m  orange1.1g022394m  orange1.1g044228m  orange1.1g043628m  orange1.1g000758m  orange1.1g005719m  orange1.1g045445m  orange1.1g039518m  orange1.1g001061m  orange1.1g014096m  orange1.1g014112m  orange1.1g011447m  orange1.1g022971m  orange1.1g028004m  orange1.1g014623m  orange1.1g025483m  orange1.1g015393m  orange1.1g016852m  orange1.1g028739m  orange1.1g028752m  orange1.1g028740m  orange1.1g032177m  orange1.1g037631m  orange1.1g036376m  orange1.1g023649m  orange1.1g013096m  orange1.1g005360m  orange1.1g004261m  orange1.1g019550m  orange1.1g026118m  orange1.1g005301m  orange1.1g007447m  orange1.1g043360m  orange1.1g041579m  orange1.1g021144m  orange1.1g021451m  orange1.1g002094m  orange1.1g026721m  orange1.1g022244m  orange1.1g009941m  orange1.1g006427m  orange1.1g028830m  orange1.1g036241m  orange1.1g026746m  orange1.1g030240m  orange1.1g004195m  orange1.1g031799m  orange1.1g046172m  orange1.1g012984m  orange1.1g002407m  orange1.1g039534m  orange1.1g005033m  orange1.1g001829m  orange1.1g001832m  orange1.1g007099m  orange1.1g014735m  orange1.1g016623m  orange1.1g014676m  orange1.1g008428m  orange1.1g013858m  orange1.1g013962m  orange1.1g027225m  orange1.1g030203m  orange1.1g008805m  orange1.1g002400m  orange1.1g006694m  orange1.1g026149m  orange1.1g038076m | AT5G64950.1  AT2G26510.1  AT2G26510.2  AT1G76710.2  AT1G76990.1  AT1G76990.2  AT1G76990.5  AT1G76990.3  AT5G66730.1  AT5G03905.1  AT3G04240.1  AT1G29100.1  AT3G55120.1  AT4G28880.1  AT1G71500.1  AT3G63380.1  AT3G26510.3  AT5G58040.1  AT2G25180.1  AT1G33330.1  AT3G10200.1  AT5G14950.1  AT5G55530.1  AT5G55530.2  AT1G16860.1  AT3G61060.1  AT5G52020.1  AT2G02800.2  AT2G02800.1  AT1G67340.1  AT5G40850.1  AT3G26510.4  AT3G26510.3  AT3G26510.1  AT3G26510.5  AT5G20220.1  AT3G18400.1  AT1G53520.1  AT2G23093.1  AT5G23980.1  AT2G05920.1  AT5G19530.1  AT3G51420.1  AT1G06150.1  AT1G06150.2  AT3G22910.1  AT1G17020.1  AT3G22740.1  AT4G14930.1  AT5G01890.1  AT1G35830.1  AT3G48820.1  AT5G04710.1  AT1G78230.1  AT1G09920.1  AT1G10070.1  AT5G43650.1  AT4G31840.1  AT3G03770.1  AT3G17130.1  AT5G17680.1  AT4G22580.1  AT5G54200.1  AT2G38330.1  AT1G80870.1  AT1G28260.2  AT1G28260.1  AT4G04450.1  AT4G22070.1  AT1G62300.1  AT4G22290.1  AT5G63120.2  AT1G20920.2  AT1G20920.1  AT5G03730.2  AT5G03730.1  AT1G18560.1  AT5G19820.1  AT1G26810.1  AT4G19830.1  AT5G44600.1 | Mitochondrial transcription termination factor family protein  Xanthine/uracil permease family protein  Xanthine/uracil permease family protein  SET domain group 26  ACT domain repeat 3  ACT domain repeat 3  ACT domain repeat 3  ACT domain repeat 3  C2H2-like zinc finger protein  Iron-sulphur cluster biosynthesis family protein  Tetratricopeptide repeat (TPR)-like superfamily protein  Heavy metal transport/detoxification superfamily protein  Chalcone-flavanone isomerase family protein  Casein kinase I-like 3  Rieske (2Fe-2S) domain-containing protein  ATPase E1-E2 type family protein / haloacid dehalogenase-like hydrolase family protein  Octicosapeptide/Phox/Bem1p family protein  Homolog of yeast FIP1 [V]  Response regulator 12  Class I peptide chain release factor  S-adenosyl-L-methionine-dependent methyltransferases superfamily protein  Golgi alpha-mannosidase II  Calcium-dependent lipid-binding (CaLB domain) family protein  Calcium-dependent lipid-binding (CaLB domain) family protein  Ubiquitin-specific protease family C19-related protein  Phloem protein 2-A13  Integrase-type DNA-binding superfamily protein  Protein kinase 2B  Protein kinase 2B  HCP-like superfamily protein with MYND-type zinc finger  Urophorphyrin methylase 1  Octicosapeptide/Phox/Bem1p family protein  Octicosapeptide/Phox/Bem1p family protein  Octicosapeptide/Phox/Bem1p family protein  Octicosapeptide/Phox/Bem1p family protein  Zinc knuckle (CCHC-type) family protein  NAC domain containing protein 58  Chalcone-flavanone isomerase family protein  Major facilitator superfamily protein  Ferric reduction oxidase 4  Subtilase family protein  S-adenosyl-L-methionine-dependent methyltransferases superfamily protein  Strictosidine synthase-like 4  Basic helix-loop-helix (bHLH) DNA-binding superfamily protein  Basic helix-loop-helix (bHLH) DNA-binding superfamily protein  ATPase E1-E2 type family protein / haloacid dehalogenase-like hydrolase family protein  Senescence-related gene 1  Homocysteine S-methyltransferase 3  Survival protein SurE-like phosphatase/nucleotidase  Leucine-rich receptor-like protein kinase family protein  VQ motif-containing protein  Glycosyltransferase family 29 (sialyltransferase) family protein  Zn-dependent exopeptidases superfamily protein  Outer arm dynein light chain 1 protein  TRAF-type zinc finger-related  Branched-chain amino acid transaminase 2  Basic helix-loop-helix (bHLH) DNA-binding superfamily protein  Early nodulin-like protein 15  Leucine-rich repeat protein kinase family protein  Plant invertase/pectin methylesterase inhibitor superfamily protein  disease resistance protein (TIR-NBS-LRR class), putative  Exostosin family protein  Transducin/WD40 repeat-like superfamily protein  MATE efflux family protein  Protein kinase superfamily protein  Telomerase activating protein Est1  Telomerase activating protein Est1  WRKY family transcription factor  WRKY DNA-binding protein 31  WRKY family transcription factor  Ubiquitin-specific protease family C19-related protein  P-loop containing nucleoside triphosphate hydrolases superfamily protein  P-loop containing nucleoside triphosphate hydrolases superfamily protein  P-loop containing nucleoside triphosphate hydrolases superfamily protein  Protein kinase superfamily protein  Protein kinase superfamily protein  BED zinc finger ;hAT family dimerisation domain  ARM repeat superfamily protein  galactosyltransferase1  FKBP-like peptidyl-prolyl cis-trans isomerase family protein  S-adenosyl-L-methionine-dependent methyltransferases superfamily protein |
| miR3443 | orange1.1g017888m  orange1.1g022660m | AT5G12040.1  AT5G12040.2 | Nitrilase/cyanide hydratase and apolipoprotein N-acyltransferase family protein  Nitrilase/cyanide hydratase and apolipoprotein N-acyltransferase family protein |
| miR3446 | orange1.1g004633m  orange1.1g004928m  orange1.1g036074m | AT5G66850.1  AT2G25930.1  AT4G22200.1 | Mitogen-activated protein kinase kinase kinase 5  Hydroxyproline-rich glycoprotein family protein  Potassium transport 2/3 |
| miR3510 | orange1.1g000791m | AT5G58610.1 | PHD finger transcription factor, putative |
| miR3638 | orange1.1g017621m | AT4G08850.1 | Leucine-rich repeat receptor-like protein kinase family protein |
| miR393 | orange1.1g010049m | AT3G18080.1 | B-S glucosidase 44 |
| miR3946 | orange1.1g008033m  orange1.1g024892m  orange1.1g025742m  orange1.1g029215m  orange1.1g007409m  orange1.1g008131m  orange1.1g014046m  orange1.1g018306m  orange1.1g015407m  orange1.1g027324m  orange1.1g029573m  orange1.1g005678m  orange1.1g010076m  orange1.1g007785m  orange1.1g041705m  orange1.1g008017m  orange1.1g021974m  orange1.1g032701m  orange1.1g011288m  orange1.1g005793m  orange1.1g004564m  orange1.1g000847m  orange1.1g016997m  orange1.1g010437m  orange1.1g027084m  orange1.1g011329m  orange1.1g017756m  orange1.1g003550m  orange1.1g021656m  orange1.1g016506m  orange1.1g028984m  orange1.1g003870m  orange1.1g018220m  orange1.1g020417m  orange1.1g013045m  orange1.1g001676m  orange1.1g011625m  orange1.1g009943m  orange1.1g030008m  orange1.1g017825m  orange1.1g017665m  orange1.1g001541m  orange1.1g002915m  orange1.1g015158m  orange1.1g008113m  orange1.1g002871m  orange1.1g026437m  orange1.1g002063m  orange1.1g001383m  orange1.1g013500m  orange1.1g002776m  orange1.1g014765m  orange1.1g034408m  orange1.1g000086m  orange1.1g011548m  orange1.1g016580m  orange1.1g020754m  orange1.1g025497m  orange1.1g005344m  orange1.1g033867m  orange1.1g000694m  orange1.1g002882m  orange1.1g018425m  orange1.1g030941m  orange1.1g026096m  orange1.1g027026m  orange1.1g014356m  orange1.1g014089m  orange1.1g008730m  orange1.1g030887m  orange1.1g013629m  orange1.1g025914m  orange1.1g007260m  orange1.1g010142m  orange1.1g016950m  orange1.1g017263m  orange1.1g020124m  orange1.1g011938m  orange1.1g028670m  orange1.1g010674m  orange1.1g014846m  orange1.1g021047m  orange1.1g026346m  orange1.1g001975m  orange1.1g005651m  orange1.1g006091m  orange1.1g007773m  orange1.1g023081m  orange1.1g003265m  orange1.1g005183m  orange1.1g031837m  orange1.1g024507m  orange1.1g020877m  orange1.1g010179m  orange1.1g000187m  orange1.1g000296m  orange1.1g004509m  orange1.1g005654m  orange1.1g011498m  orange1.1g012387m  orange1.1g014660m  orange1.1g015632m  orange1.1g017643m  orange1.1g033760m  orange1.1g013416m  orange1.1g027612m | AT1G22530.1  AT4G09650.1  AT1G21000.2  AT4G35020.3  AT3G58560.1  AT1G18870.1  AT1G64720.1  AT3G52060.1  AT1G05170.2  AT5G47310.1  AT5G47370.1  AT5G20930.1  AT3G54700.1  AT4G34370.1  AT4G25980.1  AT1G55110.1  AT5G60200.1  AT1G52740.1  AT3G57610.1  AT5G47750.1  AT3G04910.1  AT1G13220.2  AT1G13310.1  AT4G13710.2  AT3G20560.1  AT4G38510.5  AT2G17265.1  AT4G26690.1  AT2G40830.1  AT1G32740.1  AT1G49620.1  AT3G56640.1  AT3G56680.1  AT3G15140.1  AT5G25220.1  AT1G27320.1  AT2G33500.1  AT4G36920.2  AT3G49870.1  AT5G12840.4  AT3G04070.1  AT5G20280.1  AT3G42170.1  AT3G13920.1  AT3G25230.1  AT1G29400.2  AT3G07390.1  AT1G72180.1  AT2G27980.1  AT4G25990.1  AT3G22400.1  AT3G06270.1  AT1G33110.1  AT1G36160.2  AT5G49630.1  AT4G08300.1  AT4G08980.2  AT5G65430.1  AT3G10420.2  AT5G24860.1  AT3G06880.2  AT3G06670.1  AT1G48210.1  AT3G16640.1  AT4G17900.1  AT4G27670.1  AT3G13510.1  AT1G73390.1  AT2G42810.2  AT3G08890.2  AT3G22190.1  AT4G13040.1  AT5G47750.1  AT4G17180.1  AT4G18710.1  AT1G29050.1  AT2G01060.1  AT3G11440.1  AT5G11090.1  AT1G72710.1  AT5G46170.1  AT4G03270.1  AT4G03510.2  AT5G61960.1  AT1G32640.1  AT5G24300.2  AT5G24240.1  AT1G04770.1  AT5G63020.1  AT3G05350.1  AT1G08830.1  AT5G32450.1  AT5G07990.1  AT1G78900.1  AT1G01320.2  AT3G62900.1  AT2G45290.1  AT2G46040.1  AT1G01540.2  AT4G00050.1  AT2G46270.1  AT5G26751.1  AT3G62660.1  AT2G46690.1  AT5G05840.1  AT1G04760.1 | PATELLIN 2  ATP synthase delta-subunit gene  PLATZ transcription factor family protein  RAC-like 3  DNAse I-like superfamily protein  Isochorismate synthase 2  Polyketide cyclase/dehydrase and lipid transport superfamily protein  Core-2/I-branching beta-1,6-N-acetylglucosaminyltransferase family protein  Galactosyltransferase family protein  PPPDE putative thiol peptidase family protein  Homeobox-leucine zipper protein 4 (HB-4) / HD-ZIP protein  Protein kinase superfamily protein  Phosphate transporter 1;7  RING/U-box superfamily protein  Peroxidase superfamily protein  Indeterminate(ID)-domain 7  TARGET OF MONOPTEROS 6  Histone H2A protein 9  Adenylosuccinate synthase  D6 protein kinase like 2  With no lysine (K) kinase 1  Nuclear matrix constituent protein-related  Endosomal targeting BRO1-like domain-containing protein  Pectin lyase-like superfamily protein  PDI-like 5-3  ATPase, V1 complex, subunit B protein  Homoserine kinase  PLC-like phosphodiesterase family protein  RING-H2 finger C1A  SBP (S-ribonuclease binding protein) family protein  Cyclin-dependent kinase inhibitor family protein  Exocyst complex component sec15A  Single-stranded nucleic acid binding R3H protein  Polynucleotidyl transferase, ribonuclease H-like superfamily protein  KNOTTED1-like homeobox gene 3  Histidine kinase 3  B-box type zinc finger protein with CCT domain  Integrase-type DNA-binding superfamily protein  ADP-ribosylation factor-like A1C  Nuclear factor Y, subunit A1  NAC domain containing protein 47  Sucrose phosphate synthase 1F  BED zinc finger ;hAT family dimerisation domain  Eukaryotic translation initiation factor 4A1  Rotamase FKBP 1  MEI2-like protein 5  Auxin-responsive family protein  Leucine-rich receptor-like protein kinase family protein  Acyl-CoA N-acyltransferase with RING/FYVE/PHD-type zinc finger domain  CCT motif family protein  PLAT/LH2 domain-containing lipoxygenase family protein  Protein phosphatase 2C family protein  MATE efflux family protein  acetyl-CoA carboxylase 1  amino acid permease 6  nodulin MtN21 /EamA-like transporter family protein  F-BOX WITH WD-40 2  general regulatory factor 8  P-loop containing nucleoside triphosphate hydrolases superfamily protein  flowering promoting factor 1  Transducin/WD40 repeat-like superfamily protein  binding  Protein kinase superfamily protein  translationally controlled tumor protein  PLATZ transcription factor family protein  heat shock protein 21  Protein of Unknown Function (DUF239)  Endosomal targeting BRO1-like domain-containing protein  protein phosphatase 5.2  Protein of unknown function, DUF538  IQ-domain 5  Integrase-type DNA-binding superfamily protein  D6 protein kinase like 2  O-Glycosyl hydrolases family 17 protein  Protein kinase superfamily protein  TRICHOME BIREFRINGENCE-LIKE 38  myb-like HTH transcriptional regulator family protein  myb domain protein 65  serine-rich protein-related  casein kinase 1-like protein 2  F-box family protein  Cyclin D6;1  RING membrane-anchor 1  MEI2-like protein 1  Basic helix-loop-helix (bHLH) DNA-binding family protein  Glycogen/starch synthases, ADP-glucose type  Phosphatidylinositol 3- and 4-kinase ;Ubiquitin family protein  Tetratricopeptide repeat (TPR)-like superfamily protein  Disease resistance protein (CC-NBS-LRR class) family  Metallopeptidase M24 family protein  copper/zinc superoxide dismutase 1  RNA binding (RRM/RBD/RNP motifs) family protein  Cytochrome P450 superfamily protein  vacuolar ATP synthase subunit A  Tetratricopeptide repeat (TPR)-like superfamily protein  CW-type Zinc Finger  Transketolase  ARID/BRIGHT DNA-binding domain;ELM2 domain protein  Protein kinase superfamily protein  Basic helix-loop-helix (bHLH) DNA-binding superfamily protein  G-box binding factor 3  Shaggy-related kinase 11  Galacturonosyltransferase-like 7  SAUR-like auxin-responsive protein family  Protein of unknown function (DUF620)  Vesicle-associated membrane protein 726 |
| miR3953 | orange1.1g016435m  orange1.1g017142m | AT5G46590.1  AT5G22290.1 | NAC domain containing protein 96  NAC domain containing protein 89 |
| miR3979 | orange1.1g047335m  orange1.1g004351m  orange1.1g008876m  orange1.1g003467m  orange1.1g008307m | AT4G39340.1  AT3G19720.1  AT4G20140.1  AT4G07410.1  AT1G16900.1 | Protein of unknown function (DUF1278)  P-loop containing nucleoside triphosphate hydrolases superfamily protein  Leucine-rich repeat transmembrane protein kinase  Transducin family protein / WD-40 repeat family protein  Alg9-like mannosyltransferase family |
| miR4244 | orange1.1g000234m  orange1.1g000393m  orange1.1g001899m  orange1.1g042321m  orange1.1g011402m  orange1.1g021930m | AT4G32620.1  AT4G32620.2  AT1G21640.1  AT1G59970.1  AT1G05840.1  AT1G30910.1 | Enhancer of polycomb-like transcription factor protein  Enhancer of polycomb-like transcription factor protein  NAD kinase 2  Matrixin family protein  Eukaryotic aspartyl protease family protein  Molybdenum cofactor sulfurase family protein |
| miR4366 | orange1.1g040864m | AT1G24625.1 | Zinc finger protein 7 |
| miR477 | orange1.1g018483m | AT3G11340.1 | UDP-Glycosyltransferase superfamily protein |
| miR5029 | orange1.1g011764m  orange1.1g020142m | AT5G42240.1  AT1G43780.1 | Serine carboxypeptidase-like 42  Serine carboxypeptidase-like 44 |
| miR5037 | orange1.1g013267m  orange1.1g013411m  orange1.1g016066m  orange1.1g009501m  orange1.1g013426m  orange1.1g045030m  orange1.1g032502m  orange1.1g040418m  orange1.1g044628m  orange1.1g012879m | AT1G43190.1  AT2G16980.2  AT2G16990.2  AT3G15070.2  AT3G15070.1  AT1G75900.1  AT3G08710.1  AT2G37710.1  AT2G46050.1  AT1G09220.1 | Polypyrimidine tract-binding protein 3  Major facilitator superfamily protein  Major facilitator superfamily protein  RING/U-box superfamily protein  RING/U-box superfamily protein  GDSL-like Lipase/Acylhydrolase superfamily protein  Thioredoxin H-type 9  Receptor lectin kinase  Pentatricopeptide repeat (PPR-like) superfamily protein  Pentatricopeptide repeat (PPR) superfamily protein |
| miR5057 | orange1.1g042327m  orange1.1g021529m | AT5G63020.1  AT5G26680.2 | Disease resistance protein (CC-NBS-LRR class) family  5\'-3\' exonuclease family protein |
| miR5227 | orange1.1g040823m  orange1.1g030350m  orange1.1g035609m  orange1.1g029721m  orange1.1g048384m  orange1.1g041304m  orange1.1g036387m  orange1.1g014823m  orange1.1g031467m  orange1.1g007071m  orange1.1g021206m  orange1.1g016526m  orange1.1g018585m  orange1.1g003684m  orange1.1g005229m | AT2G41890.1  AT5G20885.1  AT4G27990.1  AT5G53160.2  AT3G49350.1  AT1G05577.1  AT5G23120.1  AT4G31170.1  AT2G24860.1  AT1G49890.1  AT1G33800.1  AT1G50300.1  AT1G31260.1  AT5G27220.1  AT5G61890.1 | Curculin-like (mannose-binding) lectin family protein / PAN domain-containing protein  RING/U-box superfamily protein  YGGT family protein  Regulatory components of ABA receptor 3  Ypt/Rab-GAP domain of gyp1p superfamily protein  Domain of unknown function (DUF966)  Photosystem II stability/assembly factor, chloroplast (HCF136)  Protein kinase superfamily protein  DnaJ/Hsp40 cysteine-rich domain superfamily protein  Family of unknown function (DUF566)  Protein of unknown function (DUF579)  TBP-associated factor 15  Zinc transporter 10 precursor  Frigida-like protein  Integrase-type DNA-binding superfamily protein |
| miR5233 | orange1.1g012694m | AT1G12970.1 | Plant intracellular ras group-related LRR 3 |
| miR5259 | orange1.1g005910m | AT5G42480.1 | Chaperone DnaJ-domain superfamily protein |
| miR5262 | orange1.1g005832m  orange1.1g003885m  orange1.1g045543m  orange1.1g034299m  orange1.1g000957m  orange1.1g016749m | AT1G06820.1  AT5G49890.1  AT5G54160.1  AT1G65700.3  AT2G31970.1  AT5G12130.1 | Carotenoid isomerase  Chloride channel C  O-methyltransferase 1  Small nuclear ribonucleoprotein family protein  DNA repair-recombination protein (RAD50)  Integral membrane TerC family protein |
| miR5338 | orange1.1g015878m  orange1.1g022537m  orange1.1g026124m  orange1.1g026315m  orange1.1g013131m  orange1.1g004518m | AT1G47870.2  AT1G47870.1  AT2G31380.1  AT3G12500.1  AT5G50210.1  AT5G63630.1 | Winged-helix DNA-binding transcription factor family protein  Winged-helix DNA-binding transcription factor family protein  Salt tolerance homologue  Basic chitinase  Quinolinate synthase  P-loop containing nucleoside triphosphate hydrolases superfamily protein |
| miR5368 | orange1.1g011456m  orange1.1g013011m | AT5G53840.1  AT4G01050.1 | F-box/RNI-like/FBD-like domains-containing protein  Thylakoid rhodanese-like |
| miR5498 | orange1.1g042216m | AT3G51550.1 | Malectin/receptor-like protein kinase family protein |
| miR5499 | orange1.1g002521m  orange1.1g009266m  orange1.1g042735m | AT5G04895.1  AT4G35140.1  AT4G08850.1 | DEA(D/H)-box RNA helicase family protein  Transducin/WD40 repeat-like superfamily protein  Leucine-rich repeat receptor-like protein kinase family protein |
| miR5534 | orange1.1g048798m | AT3G14470.1 | NB-ARC domain-containing disease resistance protein |
| miR5656 | orange1.1g023027m | AT2G03050.1 | Mitochondrial transcription termination factor family protein |
| miR5762 | orange1.1g010779m | AT5G06900.1 | Cytochrome P450, family 93, subfamily D, polypeptide 1 |
| miR5766 | orange1.1g034822m  orange1.1g004279m  orange1.1g001097m | AT4G27080.1  AT1G76280.3  AT1G27750.1 | PDI-like 5-4  Tetratricopeptide repeat (TPR)-like superfamily protein  Nucleic acid binding |
| miR5812 | orange1.1g043235m | AT1G74190.1 | Receptor like protein 15 |
| miR5821 | orange1.1g026276m  orange1.1g008183m  orange1.1g037143m  orange1.1g039657m | AT5G42700.1  AT4G33170.1  AT5G04730.1  AT5G04700.1 | AP2/B3-like transcriptional factor family protein  Tetratricopeptide repeat (TPR)-like superfamily protein  Ankyrin-repeat containing protein  Ankyrin repeat family protein |
| miR5834 | orange1.1g031829m  orange1.1g025843m  orange1.1g000412m  orange1.1g000656m | AT3G50810.1  AT3G12930.1  AT3G60240.2  AT3G60240.4 | Uncharacterised protein family (UPF0497)  Lojap-related protein  Eukaryotic translation initiation factor 4G  Eukaryotic translation initiation factor 4G |
| miR6025 | orange1.1g023118m  orange1.1g005832m | AT2G21940.4  AT1G06820.1 | Shikimate kinase 1  Carotenoid isomerase |
| miR6136 | orange1.1g025985m  orange1.1g002187m  orange1.1g045303m | AT5G44080.1  AT3G14470.1  AT3G14460.1 | Basic-leucine zipper (bZIP) transcription factor family protein  NB-ARC domain-containing disease resistance protein  LRR and NB-ARC domains-containing disease resistance protein |
| miR6143 | orange1.1g004065m  orange1.1g000512m  orange1.1g008925m  orange1.1g006058m  orange1.1g037093m  orange1.1g032285m  orange1.1g042761m | AT4G23640.1  AT1G15520.1  AT5G21222.1  AT5G11430.1  AT5G13910.1  AT3G04720.1  AT5G19500.1 | Potassium transporter family protein  Pleiotropic drug resistance 12  Protein kinase family protein  SPOC domain / Transcription elongation factor S-II protein  Integrase-type DNA-binding superfamily protein  Pathogenesis-related 4  Tryptophan/tyrosine permease |
| miR6180 | orange1.1g014985m  orange1.1g029357m  orange1.1g032835m  orange1.1g009468m  orange1.1g002564m  orange1.1g044974m  orange1.1g006131m  orange1.1g015433m  orange1.1g029885m  orange1.1g031178m | AT1G18040.1  AT4G35335.1  AT1G14140.1  AT3G58790.1  AT4G04940.1  AT2G18950.1  AT3G06500.1  AT1G52920.1  AT5G47710.1  AT5G47710.2 | Cyclin-dependent kinase D1;3  Nucleotide-sugar transporter family protein  Mitochondrial substrate carrier family protein  Galacturonosyltransferase 15  Transducin family protein / WD-40 repeat family protein  Homogentisate phytyltransferase 1  Plant neutral invertase family protein  G protein coupled receptor  Calcium-dependent lipid-binding (CaLB domain) family protein  Calcium-dependent lipid-binding (CaLB domain) family protein |
| miR6214 | orange1.1g014994m  orange1.1g007067m  orange1.1g020905m  orange1.1g037100m  orange1.1g019091m  orange1.1g006896m  orange1.1g017349m  orange1.1g011689m  orange1.1g044959m  orange1.1g007550m  orange1.1g032005m  orange1.1g037661m  orange1.1g027351m  orange1.1g041119m  orange1.1g026607m | AT4G19660.1  AT5G01760.1  AT5G24490.1  AT1G23710.1  AT4G25720.1  AT3G07660.1  AT2G24540.1  AT4G27600.1  AT1G75240.1  AT5G45110.1  AT3G11780.1  AT5G37380.4  AT3G03530.1  AT5G11590.1  AT2G44840.1 | NPR1-like protein 4  ENTH/VHS/GAT family protein  30S ribosomal protein, putative  Protein of unknown function (DUF1645)  Glutaminyl cyclase  Kinase-related protein of unknown function (DUF1296)  Galactose oxidase/kelch repeat superfamily protein  PfkB-like carbohydrate kinase family protein  Homeobox protein 33  NPR1-like protein 3  MD-2-related lipid recognition domain-containing protein / ML domain-containing protein  Chaperone DnaJ-domain superfamily protein  Non-specific phospholipase C4  Integrase-type DNA-binding superfamily protein  Ethylene-responsive element binding factor 13 |
| miR6247 | orange1.1g044169m | AT2G27610.1 | Tetratricopeptide repeat (TPR)-like superfamily protein |
| miR6260 | orange1.1g003752m  orange1.1g029026m  orange1.1g002855m  orange1.1g041599m  orange1.1g043518m  orange1.1g046902m  orange1.1g007512m  orange1.1g010903m  orange1.1g036391m  orange1.1g042312m  orange1.1g002941m  orange1.1g033274m  orange1.1g021990m  orange1.1g044417m  orange1.1g002676m  orange1.1g003678m  orange1.1g002964m  orange1.1g003319m  orange1.1g047631m  orange1.1g001045m  orange1.1g003451m  orange1.1g000565m  orange1.1g027807m  orange1.1g008817m  orange1.1g015356m  orange1.1g023188m  orange1.1g019887m  orange1.1g035767m  orange1.1g007303m | AT5G42480.1  AT1G64650.1  AT5G22640.1  AT1G49330.1  AT3G55360.1  AT4G12560.2  AT4G34200.1  AT5G15130.1  AT4G12560.2  AT3G50845.1  AT5G57590.1  AT4G39340.1  AT5G13890.1  AT2G42770.1  AT1G55250.3  AT1G55250.1  AT5G04460.1  AT5G04460.2  AT2G16485.1  AT1G66730.1  AT5G02860.1  AT2G20190.1  AT1G17100.1  AT5G23960.2  AT5G55730.2  AT5G15530.1  AT3G16770.1  AT3G60900.1  AT5G57190.1 | Chaperone DnaJ-domain superfamily protein  Major facilitator superfamily protein  MORN (Membrane Occupation and Recognition Nexus) repeat-containing protein  Hydroxyproline-rich glycoprotein family protein  3-oxo-5-alpha-steroid 4-dehydrogenase family protein  F-box and associated interaction domains-containing protein  D-3-phosphoglycerate dehydrogenase  WRKY DNA-binding protein 72  F-box and associated interaction domains-containing protein  Protein of unknown function (DUF59)  Adenosylmethionine-8-amino-7-oxononanoate transaminases  Protein of unknown function (DUF1278)  Family of unknown function (DUF716)  Peroxisomal membrane 22 kDa (Mpv17/PMP22) family protein  Histone mono-ubiquitination 2  Histone mono-ubiquitination 2  RING/U-box superfamily protein  RING/U-box superfamily protein  Nucleic acid binding;zinc ion binding;DNA binding  DNA LIGASE 6  Pentatricopeptide repeat (PPR) superfamily protein  CLIP-associated protein  SOUL heme-binding family protein  Terpene synthase 21  FASCICLIN-like arabinogalactan 1  Biotin carboxyl carrier protein 2  Ethylene-responsive element binding protein  FASCICLIN-like arabinogalactan-protein 10  Phosphatidylserine decarboxylase 2 |
| miR6262 | orange1.1g048798m | AT3G14470.1 | NB-ARC domain-containing disease resistance protein |
| miR6281 | orange1.1g005908m  orange1.1g007409m  orange1.1g005678m  orange1.1g007785m  orange1.1g016997m  orange1.1g027084m  orange1.1g021656m  orange1.1g013045m  orange1.1g001676m  orange1.1g015158m  orange1.1g002871m  orange1.1g037584m  orange1.1g034408m  orange1.1g011548m  orange1.1g016580m  orange1.1g025497m  orange1.1g030887m  orange1.1g013629m  orange1.1g025914m  orange1.1g016950m  orange1.1g028670m  orange1.1g007898m  orange1.1g010674m  orange1.1g026346m  orange1.1g001975m  orange1.1g006091m  orange1.1g005183m  orange1.1g020877m  orange1.1g000296m  orange1.1g004509m  orange1.1g011498m  orange1.1g012387m  orange1.1g017643m | AT3G63150.1  AT3G58560.1  AT5G20930.1  AT4G34370.1  AT1G13310.1  AT3G20560.1  AT2G40830.1  AT5G25220.1  AT1G27320.1  AT3G13920.1  AT1G29400.2  AT5G47550.1  AT1G33110.1  AT5G49630.1  AT4G08300.1  AT5G65430.1  AT3G08890.2  AT3G22190.1  AT4G13040.1  AT4G18710.1  AT5G11090.1  AT3G13810.1  AT1G72710.1  AT4G03510.2  AT5G61960.1  AT5G24300.2  AT3G05350.1  AT5G07990.1  AT3G62900.1  AT2G45290.1  AT1G01540.2  AT4G00050.1  AT3G62660.1 | MIRO-related GTP-ase 2  DNAse I-like superfamily protein  Protein kinase superfamily protein  RING/U-box superfamily protein  Endosomal targeting BRO1-like domain-containing protein  PDI-like 5-3  RING-H2 finger C1A  KNOTTED1-like homeobox gene 3  histidine kinase 3  eukaryotic translation initiation factor 4A1  MEI2-like protein 5  Cystatin/monellin superfamily protein  MATE efflux family protein  Amino acid permease 6  Nodulin MtN21 /EamA-like transporter family protein  General regulatory factor 8  Protein of unknown function, DUF538  IQ-domain 5  Integrase-type DNA-binding superfamily protein  Protein kinase superfamily protein  Serine-rich protein-related  Indeterminate(ID)-domain 11  Casein kinase 1-like protein 2  RING membrane-anchor 1  MEI2-like protein 1  Glycogen/starch synthases, ADP-glucose type  Metallopeptidase M24 family protein  Cytochrome P450 superfamily protein  CW-type Zinc Finger  Transketolase  Protein kinase superfamily protein  Basic helix-loop-helix (bHLH) DNA-binding superfamily protein  Galacturonosyltransferase-like 7 |
| miR7127 | orange1.1g004943m  orange1.1g000624m | AT5G51340.1  AT2G25050.1 | Tetratricopeptide repeat (TPR)-like superfamily protein  Actin-binding FH2 (Formin Homology) protein |
| miR7128 | orange1.1g010502m  orange1.1g007945m | AT3G18040.1  AT2G32640.1 | MAP kinase 9  Lycopene beta/epsilon cyclase protein |
| miR7539 | orange1.1g018368m  orange1.1g033898m  orange1.1g027937m  orange1.1g006487m  orange1.1g005922m  orange1.1g001894m  orange1.1g003485m  orange1.1g032701m  orange1.1g017756m  orange1.1g031528m  orange1.1g035290m  orange1.1g003692m  orange1.1g004392m  orange1.1g005555m  orange1.1g018220m  orange1.1g044685m  orange1.1g028655m  orange1.1g002698m  orange1.1g019812m  orange1.1g021699m  orange1.1g024586m  orange1.1g016580m  orange1.1g029576m  orange1.1g032330m  orange1.1g020124m  orange1.1g031504m  orange1.1g021047m  orange1.1g028026m | AT5G42520.1  AT5G42000.1  AT5G51110.1  AT2G02080.1  AT2G25970.1  AT5G60170.1  AT5G60170.2  AT1G52740.1  AT2G17265.1  AT2G14530.1  AT1G10290.1  AT1G49630.2  AT1G49630.3  AT1G49630.1  AT3G56680.1  AT3G04650.1  AT3G01470.1  AT2G42600.1  AT2G36810.1  AT5G05000.2  AT5G05000.3  AT4G08300.1  AT1G78020.1  AT4G28440.1  AT2G01060.1  AT2G33990.1  AT4G03270.1  AT4G19100.1 | Basic pentacysteine 6  ORMDL family protein  Transcriptional coactivator/pterin dehydratase  indeterminate(ID)-domain 4  KH domain-containing protein  RNA binding (RRM/RBD/RNP motifs) family protein  RNA binding (RRM/RBD/RNP motifs) family protein  Histone H2A protein 9  Homoserine kinase  TRICHOME BIREFRINGENCE-LIKE 13  Dynamin-like protein 6  Presequence protease 2  Presequence protease 2  Presequence protease 2  Single-stranded nucleic acid binding R3H protein  FAD/NAD(P)-binding oxidoreductase family protein  Homeobox 1  Phosphoenolpyruvate carboxylase 2  ARM repeat superfamily protein  Translocon at the outer envelope membrane of chloroplasts 34  Translocon at the outer envelope membrane of chloroplasts 34  Nodulin MtN21 /EamA-like transporter family protein  Protein of unknown function (DUF581)  Nucleic acid-binding, OB-fold-like protein  Myb-like HTH transcriptional regulator family protein  IQ-domain 9  Cyclin D6;1  Protein of unknown function (DUF3464) |
| miR7767 | orange1.1g031715m | AT3G52560.1 | Ubiquitin E2 variant 1D-4 |
| miR7785 | orange1.1g030776m | AT5G19440.1 | NAD(P)-binding Rossmann-fold superfamily protein |
| miR7837 | orange1.1g013681m | AT3G18270.1 | Cytochrome P450, family 77, subfamily A, polypeptide 5 pseudogene |
| miR7838 | orange1.1g028094m | AT5G51990.1 | C-repeat-binding factor 4 |
| miR7841 | orange1.1g041450m  orange1.1g039871m  orange1.1g035790m  orange1.1g005247m  orange1.1g005251m  orange1.1g006703m  orange1.1g038607m | AT3G42640.1  AT3G63470.1  AT5G49980.1  AT5G03730.1  AT5G03730.2  AT4G24480.1  AT4G31940.1 | H(+)-ATPase 8  Serine carboxypeptidase-like 40  Auxin F-box protein 5  Protein kinase superfamily protein  Protein kinase superfamily protein  Protein kinase superfamily protein  Cytochrome P450, family 82, subfamily C, polypeptide 4 |
| miR8005 | orange1.1g012326m  orange1.1g037771m  orange1.1g010176m  orange1.1g035432m | AT5G67630.1  AT1G58100.1  AT1G04280.1  AT3G06680.1 | P-loop containing nucleoside triphosphate hydrolases superfamily protein  TCP family transcription factor  P-loop containing nucleoside triphosphate hydrolases superfamily protein  Ribosomal L29e protein family |
| miR8036 | orange1.1g039852m  orange1.1g037695m  orange1.1g001805m | AT3G14470.1  AT1G30290.1  AT1G27170.1 | NB-ARC domain-containing disease resistance protein  Tetratricopeptide repeat (TPR)-like superfamily protein  Transmembrane receptors;ATP binding |
| miR8124 | orange1.1g042449m | AT3G51810.1 | Stress induced protein |
| miR8141 | orange1.1g009354m | AT2G47310.1 | Flowering time control protein-related / FCA gamma-related |
| miR8145 | orange1.1g035624m | AT3G44900.1 | Cation/H+ exchanger 4 |
| miR833 | orange1.1g047519m | AT1G45616.1 | Receptor like protein 6 |
| miR838 | orange1.1g009145m  orange1.1g009474m  orange1.1g003745m  orange1.1g005921m  orange1.1g002711m | AT4G17410.2  AT2G30390.1  AT4G29950.1  AT4G29950.2  AT1G09090.2 | DWNN domain, a CCHC-type zinc finger  Ferrochelatase 2  Ypt/Rab-GAP domain of gyp1p superfamily protein  Ypt/Rab-GAP domain of gyp1p superfamily protein  Respiratory burst oxidase homolog B |
