# Supplementary material for: Boron-deficiency-responsive microRNAs and their targets in Citrus sinensis leaves
Source: BMC Plant Biol. 2015 Nov 4;15:271. doi: 10.1186/s12870-015-0642-y (PMC4634795; doi:10.1186/s12870-015-0642-y)
Supplement: Additional file 7: — List of target genes for parts of novel miRNAs in Citrus sinensis leaves. (DOC 33 kb) [file 12870_2015_642_MOESM7_ESM.doc]

**Additional file 7: List of target genes for parts of novel miRNAs in *Citrus sinensis* leaves.**

| *miRNA* | *Assession* | *Homology* | *Target genes* |
| --- | --- | --- | --- |
| novel_mir_132 | orange1.1g004928m  orange1.1g020365m  orange1.1g036074m  orange1.1g009354m | AT2G25930.1  AT2G25850.4  AT4G22200.1  AT2G47310.1 | Hydroxyproline-rich glycoprotein family protein  P oly(A) polymerase 2  Potassium transport 2/3  Flowering time control protein-related / FCA gamma-related |
| novel_mir_134 | orange1.1g029650m  orange1.1g009653m  orange1.1g011640m orange1.1g021420m  orange1.1g032310m  orange1.1g046416m  orange1.1g016971m | AT1G53160.1  AT1G69170.1  AT5G43270.2  AT5G50670.1  AT2G33810.1  AT2G42200.1  AT5G50570.2 | Squamosa promoter binding protein-like 4  Squamosa promoter-binding protein-like (SBP domain) transcription factor family protein  Squamosa promoter binding protein-like 2  Squamosa promoter-binding protein-like (SBP domain) transcription factor family protein  Squamosa promoter binding protein-like 3  Squamosa promoter binding protein-like 9  Squamosa promoter-binding protein-like (SBP domain) transcription factor family protein |
| novel_mir_224 | orange1.1g045509m | AT1G69310.2 | WRKY DNA-binding protein 57 |
| novel_mir_35 | orange1.1g019148m  orange1.1g019183m | AT5G51410.1  AT5G51410.2 | LUC7 N_terminus domain-containing protein  LUC7 N_terminus domain-containing protein |
| novel_mir_52 | orange1.1g001616m | AT3G63380.1 | ATPase E1-E2 type family protein / haloacid dehalogenase-like hydrolase family protein |
| novel_mir_59 | orange1.1g010034m  orange1.1g010770m | AT2G36360.1  AT2G36360.3 | Galactose oxidase/kelch repeat superfamily protein  Galactose oxidase/kelch repeat superfamily protein |
