# Supplementary material for: Boron-deficiency-responsive microRNAs and their targets in Citrus sinensis leaves
Source: BMC Plant Biol. 2015 Nov 4;15:271. doi: 10.1186/s12870-015-0642-y (PMC4634795; doi:10.1186/s12870-015-0642-y)
Supplement: Additional file 8: — List of stem loop qRT-PCR primers. (DOC 61 kb) [file 12870_2015_642_MOESM8_ESM.doc]

**Additional file 8: List of stem loop qRT-PCR primers.**

| miRNA | Sequence | RT-primer: | Forward primer |
| --- | --- | --- | --- |
| miR158 | TCCCAAATGTAGACAAAGCA | GTCGTATCCAGTGCAGGGTCCGAGGTATTCGCACTGGATACGACTGCTTT | CGCGGTCCCAAATGTAGAC |
| miR159 | TTTGGATTGAAGGGAGCTCTA | GTCGTATCCAGTGCAGGGTCCGAGGTATTCGCACTGGATACGACTAGAGC | CGGAGTTTGGATTGAAGGGA |
| miR160 | GCGTACGAGGAGCCAAGCATA | GTCGTATCCAGTGCAGGGTCCGAGGTATTCGCACTGGATACGACTATGCT | CATGAGCGTACGAGGAGCCA |
| miR164 | TGGAGAAGCAGGGCACGTGCA | GTCGTATCCAGTGCAGGGTCCGAGGTATTCGCACTGGATACGACTGCACG | GAATTGGAGAAGCAGGGCA |
| miR393 | TCATGCGATCCCTTCGGAATT | GTCGTATCCAGTGCAGGGTCCGAGGTATTCGCACTGGATACGACAATTCC | CGCGTCATGCGATCCCTTC |
| miR408 | ACGGGGAACAGGCAGAGCATG | GTCGTATCCAGTGCAGGGTCCGAGGTATTCGCACTGGATACGACCATGCT | CAACACGGGGAACAGGCAG |
| miR477 | ACCTCCCTCGAAGGCTTCCAA | GTCGTATCCAGTGCAGGGTCCGAGGTATTCGCACTGGATACGACTTGGAA | CAACACCTCCCTCGAAGGC |
| miR782 | ACAAACAGAGTTGGATTTCTT | GTCGTATCCAGTGCAGGGTCCGAGGTATTCGCACTGGATACGACAAGAAA | CGGCACAAACAGAGTTGGA |
| miR1446 | CGAACTCTCTCCCTCAACGGC | GTCGTATCCAGTGCAGGGTCCGAGGTATTCGCACTGGATACGACGCCGTT | CAGCCGAACTCTCTCCCTC |
| miR1535 | CTTCTTTGTGGTAGATTGTTT | GTCGTATCCAGTGCAGGGTCCGAGGTATTCGCACTGGATACGACAAACAA | CGCGCCTTCTTTGTGGTAGA |
| miR2099 | AAAGGCTGTACGTTATTT | GTCGTATCCAGTGCAGGGTCCGAGGTATTCGCACTGGATACGACAAATAA | CCGCAGAAAGGCTGTACG |
| miR2643 | TTTGGGACGAGATTGAGA | GTCGTATCCAGTGCAGGGTCCGAGGTATTCGCACTGGATACGACTCTCAA | CAGCCAGTTTGGGACGAGA |
| miR2648 | TAGCATGGGATAACAGAT | GTCGTATCCAGTGCAGGGTCCGAGGTATTCGCACTGGATACGACATCTGT | CGCCAGGTAGCATGGGATA |
| miR2928 | AAGAAGAAGAAGTTTGTT | GTCGTATCCAGTGCAGGGTCCGAGGTATTCGCACTGGATACGACAACAAA | CGGCGCAAGAAGAAGAAG |
| miR3446 | CTGGAAGCAACTGTGGCACGG | GTCGTATCCAGTGCAGGGTCCGAGGTATTCGCACTGGATACGACCCGTGC | CAGCAGCTGGAAGCAACTGTG |
| miR3946 | GTAGAGAGAGAGAGAGAGAGCAA | GTCGTATCCAGTGCAGGGTCCGAGGTATTCGCACTGGATACGACTTGCTC | CGCGGTAGAGAGAGAGAGAGA |
| miR3953 | TTGAGTTCTGCAAGCCGTCGA | GTCGTATCCAGTGCAGGGTCCGAGGTATTCGCACTGGATACGACTCGACG | CGCGTTGAGTTCTGCAAGC |
| miR5037 | ATGGAACTTTGAAGGCCG | GTCGTATCCAGTGCAGGGTCCGAGGTATTCGCACTGGATACGACCGGCCT | CGGCGGATGGAACTTTGA |
| miR5227 | TGAAGATGAAGACGATGATGAAGA | GTCGTATCCAGTGCAGGGTCCGAGGTATTCGCACTGGATACGACTCTTCA | CGCGTGAAGATGAAGACGATGA |
| miR5259 | CAAGGGGTATTTGGATGGACA | GTCGTATCCAGTGCAGGGTCCGAGGTATTCGCACTGGATACGACTGTCCA | CGCGCAAGGGGTATTTGGA |
| miR5266 | CGGGGGACGGTCTGGGAACG | GTCGTATCCAGTGCAGGGTCCGAGGTATTCGCACTGGATACGACCGTTCC | GAACACGGGGGACGGTCTG |
| miR5262 | TCTTCAAGAGACTCAATTT | GTCGTATCCAGTGCAGGGTCCGAGGTATTCGCACTGGATACGACAAATTG | GCCGCTCTTCAAGAGACT |
| miR6025 | TACCAACAAGAGATGAACATT | GTCGTATCCAGTGCAGGGTCCGAGGTATTCGCACTGGATACGACAATGTT | CGCCGTACCAACAAGAGATG |
| miR6214 | CGACACGAAGCAGACACGACA | GTCGTATCCAGTGCAGGGTCCGAGGTATTCGCACTGGATACGACTGTCGT | CAGAACGACACGAAGCAGAC |
| miR6260 | TGGAGTGGGAGTGGGAGT | GTCGTATCCAGTGCAGGGTCCGAGGTATTCGCACTGGATACGACACTCCC | CGACGCTGGAGTGGGAGT |
| miR7539 | AGAGAGAGAGAGGACAAGG | GTCGTATCCAGTGCAGGGTCCGAGGTATTCGCACTGGATACGACCCTTGT | CGCAGAGAGAGAGAGAGG |
| miR7841 | GGGGATTCTCTCAAGCAAA | GTCGTATCCAGTGCAGGGTCCGAGGTATTCGCACTGGATACGACTTTGCT | CACAACGGGGATTCTCTCA |
| Reverse primer | CAGTGCAGGGTCCGAGGT |  |  |

*Actin* (AEK97331.1) was used as an internal standard and the reverse (5´→3´) and forward (5´→3´) primers were GCTTGGAGCAAGTGCTGTGATT and AGAACTATGAACTGCCTGATGGC, respectively.
