# Supplementary material for: Boron-deficiency-responsive microRNAs and their targets in Citrus sinensis leaves
Source: BMC Plant Biol. 2015 Nov 4;15:271. doi: 10.1186/s12870-015-0642-y (PMC4634795; doi:10.1186/s12870-015-0642-y)
Supplement: Additional file 9: — Specific primer pairs used for qRT-PCR expression analysis of selected miRNA target genes. (DOC 160 kb) [file 12870_2015_642_MOESM9_ESM.doc]

**Additional file 9: Specific primer pairs used for qRT-PCR expression analysis of selected miRNA target genes.**

| miRNA | Accession No. | Homology | Target genes | Forward primer (5´→3´) | Reverse primer (5´→3´) |
| --- | --- | --- | --- | --- | --- |
| miR158 | orange1.1g022993m | AT1G69840.1 | SPFH/Band 7/PHB domain-containing membrane-associated protein family | GAACTCGAAAAGGCCATGTCTC | TGACGCTGACGTGCAATACC |
|  |  | AT2G03210 | Fucosyltransferase 2 | CAAACCAACCCAAATCCACCT | TGCGGGACAACATTACCAGTG |
|  | orange1.1g001709m | AT3G07400 | Lipase class 3 family protein | GCAGCCAGCCCTTTCACTTC | CTGACAGCACCAGGCATAACATA |
| miR159 | orange1.1g039708m | AT5G06100.2 | MYB domain protein 33 | GGATGAAGAAGTCAATGAGGGTG | GGCTGATGATCGGTAGTTGTAGC |
|  | orange1.1g044979m | AT4G27330.1 | Sporocyteless (SPL) | TGGGGTTCTGAATGGAGGGT | CGTCATTGTGCTTGTTGCTTGG |
|  | orange1.1g046419m | AT4G26930.1 | MYB domain protein 97 | TTTGACAAGCACTGGTTCGGTT | CTGAAGATTGGCAATTTGAGGC |
|  | orange1.1g011938m | AT3G11440.1 | MYB domain protein 65 | GAACATCTTCCCCACAACCTTTAC | TTCCGGGTGCAACTATCAGC |
|  | orange1.1g038795m | AT3G60460.1 | MYB -like HTH transcriptional regulator family protein | TCCCTCAGATTTCTCAACCACAAC | CATTTTCGTCCTTCATGCCAGT |
| miR160 | orange1.1g004896m | AT2G28350.1 | ARF10 | AGGTTTTCTTGGAGTCAGAGGATG | CACTGTCACTGCCAGAACCCAT |
|  | orange1.1g005075m | AT4G30080.1 | ARF16 | ACTTCAACAACAAGGCTTACCCG | GCGACCTACATCCTCTGATTCCAT |
|  | orange1.1g008078m | AT1G77850.1 | ARF17 | CTTCTTTCCAGTGAACGGTTTAGG | CTGTGGCTATCGGGTGATAAGTT |
| miR164 | orange1.1g030909m | AT1G56010.2 | NAC domain containing protein 1 | AGGGCAGCCTTGTCGGTATG | GACGAAGCCTTAGGAGAATTAGCA |
|  | orange1.1g047710m | AT5G53950.1 | NAC domain transcriptional regulator superfamily protein | TGGCTTGAATGCTTTCCCTA | CATCACCGTTGACCAGTTTG |
|  | orange1.1g017827m | AT5G61430.1 | NAC domain containing protein 100 | AGGGTTCTGGTGGGAAAAGG | GAGATGGGAGGAGGATTGTTGA |
|  | orange1.1g017636m | AT3G08030.1 | Protein of unknown function, DUF642 | GACATGCGGCAGTTGAGCTT | GAACCCACCTTTTCCCTTTGAT |
| miR393 | orange1.1g010049m | AT3G18080.1 | B-S glucosidase 44 | CAACAGGCTACCCAAGTTCACA | CCCACTTGCTTTGGTTGTTTCA |
|  | orange1.1g007916m | At3g62980 | TIR1 | ACTCCAACGACTTTCAGTTTCAGG | CTGCTTTGCCAATCGCCTAC |
|  |  | At4g03190 | AFB1 | TCCCTTTGGATGTCATCTTGCT | CGGTCCTGCGACTGTTCTGTA |
|  | orange1.1g008325m | At3g26810 | AFB2 | AAGCGTCTAAGGCGATTGTCA | CGCATTGTCTCATACTTTCCCAC |
|  |  | At1g12820 | AFB3 | ACATTTCAATCACTGGACGAGGGC | CGAGCTGTTCCGCGTACATACC |
| miR408 | orange1.1g013075m | At2g30210 | LACCASE 3 | GGTAATGTAAGCCGAGGTCTATGG | TGAGGTTGAACTTGGCTGTATCTG |
|  | orange1.1g041358m | At5g05390 | LACCASE 12 | ACCACCACTGCCATTGTCCA | CCTGCCAGCAAATCTGTCCC |
|  |  | At5g07130 | LACCASE 13 | CGACCCACCACGAAGAAACA | AGACCATTGCTAAACCCCAGAA |
|  | orange1.1g048131m | At2g02850 | PLANTACYANIN | ATGGCGGTTGGGTCTTTGGT | CTTAGCATCACTCGCAGGTTTG |
| miR477 | orange1.1g018483m | AT3G11340.1 | UDP-Glycosyltransferase superfamily protein | CGGGGATCAAAACCTAAACATG | CTCTTTGCCTTCTGTATCCACCAT |
| miR782 |  | HQ202267 | MYB transcription factor (MYBML2) | ACCCACAAGCGTAAAGACAC | AGAAACTTGGACTCACGGACT |
|  | orange1.1g039969m | NM_001112290 | Protein disulfide isomerase (PDIL5-1) | CGTGAAAGTGAATGCGAAGGA | CCGGGGATGAATCAGGATAAG |
| miR1446 | orange1.1g037028m | AT1G14920.1 | GRAS family transcription factor family protein | ACCAAAGTATGACACGAAACGAGC | CCATCCCAGAACCAAGCACC |
| miR1535 | orange1.1g001616m | AT3G63380.1 | ATPase E1-E2 type family protein/haloacid dehalogenase-like hydrolase family protein | TAGCGGCTGTTTCTGCTGGTG | TTCAGGACTCACGTTGAAGATGG |
|  | orange1.1g015157m | AT3G58060.1 | Cation efflux family protein | GTCATACAGCATCACCCTCAAATC | CTTCTGGCAGCTCAATATCAACCT |
| miR2099 | orange1.1g017694m | AT3G22830.1 | Heat shock transcription factor A6B | AAGCTGAACCTTTGGAATACGG | CCCACTTCGCTACCAGGCATAT |
| miR2643 | orange1.1g018307m | AT1G12500.1 | Nucleotide-sugar transporter family protein | CAGCGAGCCCCTGTTTCATT | TTGCGGCCACATTCCCTTCA |
|  | orange1.1g020050m | AT5G19890.1 | Peroxidase superfamily protein | TGCCAATGCTCTTCCTTCACC | CCGTATTGTTTCCGTCACCGT |
| miR2648 | orange1.1g003798m | AT5G58460.1 | Cation/H^+^ exchanger 25 | GAGGCTCAATGCGAAGACACC | CGGCTCCATTTCTCACCACC |
| miR2928 | orange1.1g007099m | AT4G04450.1 | WRKY family transcription factor | AGTTGGTTGCCCAGTTCGTAAG | GCAGCAGGAGGTAAGGGATGAT |
|  | orange1.1g014735m | AT4G22070.1 | WRKY DNA-binding protein 31 | AGGAACCCCACCGAACATTG | TCGAGCACGAACTGAGACACG |
|  | orange1.1g016623m | AT1G62300.1 | WRKY family transcription factor | AGACCTTACTCATTCACCAAACCC | GGCACTTACAGTGTCAGCCAACT |
| miR3446 | orange1.1g004633m | AT5G66850.1 | Mitogen-activated protein kinase kinase kinase 5 | TTTGTCGGTCCCTATGCGTGTT | CGAGTCAGGCAGTGATTCAGTTTT |
|  | orange1.1g004928m | AT2G25930.1 | Hydroxyproline-rich glycoprotein family protein | TGGGGTTATGCCTGGTGCTC | CCTGAAACTGCCTTACTGGTGGA |
|  | orange1.1g036074m | AT4G22200.1 | Potassium transport 2/3 | TATTCCCTCCTCGGCATTCT | AACCTGAGTCGCAACCCAAA |
| miR3946 | orange1.1g029573m | AT5G47370.1 | Homeobox-leucine zipper protein 4 (HB-4) / HD-ZIP protein | GAGAAGCGAGCGGGAACCTA | TCGGAGAACTAACAGAAGCCTGA |
|  | orange1.1g041705m | AT4G25980.1 | Peroxidase superfamily protein | CAGTCTTGCTTAGGCTCCATTTC | TGGTAGCTCGGTCCATTTGATAG |
|  | orange1.1g031837m | AT1G08830.1 | Copper/zinc superoxide dismutase 1 | GGTCTCAAGCCTGGTCCTCAT | GGACTACAACAGCCCTTCCAATAA |
|  | orange1.1g016997m | AT1G13310.1 | Endosomal targeting BRO1-like domain-containing protein | TGTTGCTGCCGATATGCTCA | CCATCTTTTGGGACGGTAAGG |
|  | orange1.1g014089m | AT1G73390.1 | Endosomal targeting BRO1-like domain-containing protein | CTGGCAGCAGGCTCAAGATAA | GGTTCCCCAAAGTGGAGGATTT |
|  | orange1.1g027084m | AT3G20560.1 | PDI-like 5-3 | GAGTGATGTTCAGCGATTGATACC | GCTTTGAACCAGGCTGCTATG |
|  | orange1.1g017665m | AT3G04070.1 | NAC domain containing protein 47 | CGCATCTACCGCAAGTCCCA | GGTGCTGCTCTAAAATCCCTGAAT |
|  | orange1.1g010076m | AT3G54700.1 | Phosphate transporter 1;7 | CCTAACAGAATCGGGTTCGTGA | ACCAACAATAGCCCCAGCCT |
|  | orange1.1g034408m | AT1G33110.1 | MATE efflux family protein | GAGGAAAATGATGTTGATGGGC | ATCTTGTGAAAATAGCGGGAGC |
|  | orange1.1g027612m | AT1G04760.1 | Vesicle-associated membrane protein 726 | GCTCCTGCCAATGGCCTGAA | CGAAAGTCTTGTGCCTGCTGATG |
|  | orange1.1g027026m | AT4G27670.1 | Heat shock protein 21 | GCCCCAGTTTCATCTCCAGG | AGGCGTTCTTCCCCGTCTGT |
|  | orange1.1g020124m | AT2G01060.1 | MYB-like HTH transcriptional regulator family protein | CCAGGACAAGGCTGCCAAAG | CGATGCCCGAGATGAAGGAT |
|  | orange1.1g011938m | AT3G11440.1 | MYB domain protein 65 | GATCCTCTTTCTCCTTTGGGTCA | CGTCTCCACTGGTGCTTGTTCAT |
|  | orange1.1g005651m | AT1G32640.1 | Basic helix-loop-helix (bHLH) DNA-binding family protein | GAAGATAATTGGATGGGATGCG | GCTCCTGCGTGTAAAACCGAC |
|  | orange1.1g012387m | AT4G00050.1 | Basic helix-loop-helix (bHLH) DNA-binding superfamily protein | GGCAATGCAACAACAACTTCAG | AGGAATGTGGATAACGGGTCTG |
|  | orange1.1g004509m | AT2G45290.1 | Transketolase | CGGCTGACGGTAATGAAACTG | CCCAAGAAACAAAGGAGACAACTC |
|  | orange1.1g033760m | AT2G46690.1 | SAUR-like auxin-responsive protein family | GAGGAGCAGCAGCGAGTTGT | CATGATGATGGTGATGAAGGGA |
| miR3953 | orange1.1g016435m | AT5G46590.1 | NAC domain containing protein 96 | TCAGTTGGACGGTGAGCAGG | AAGGATTCGGTTTCAGAGTTCG |
|  | orange1.1g017142m | AT5G22290.1 | NAC domain containing protein 89 | GGTTCTAGTTGTGGTAGCGGTTTA | GGATTCGGTTCCAGTGTTCG |
| miR5037 | orange1.1g013411m | AT2G16980.2 | Major facilitator superfamily protein | GGTCCTCCTGGGTTCCTTATGC | AGCCTTCCCCTGTTCGTTGG |
|  | orange1.1g016066m | AT2G16990.2 | Major facilitator superfamily protein | GGTCCTCCTGGGTTCCTTATGC | TGAGCCTTCCCCTGTTCGTT |
| miR5262 | orange1.1g005832m | AT1G06820.1 | Carotenoid isomerase | GGGACTGGCTCAAAAGGACTATG | TTGCTATGGTGGTGGGCGTA |
|  | orange1.1g003885m | AT5G49890.1 | Chloride channel C | ATGGTTTCCCAGTGATAGATGAGC | TGTCCAAATCTTCTAGCTTGACCC |
| miR5266 | orange1.1g040022m | AT4G13510.1 | Ammonium transporter 1;1 | TGGTGATTACTGGGTGGGTGA | CCTGCCATTTCCTCGTCTGTT |
| miR5227 | orange1.1g031467m | AT2G24860.1 | DnaJ/Hsp40 cysteine-rich domain superfamily protein | TAACAAGAAAGTCACCGATGCG | GAAATTAACCAGGCTTGCTCCA |
|  | orange1.1g018585m | AT1G31260.1 | Zinc transporter 10 precursor | CCTCGGCAGGACTACTCATCTACA | CCAAGCAAAACGGCAGCATA |
| miR5929 | orange1.1g005910m | AT5G42480.1 | Chaperone DnaJ-domain superfamily protein | GAGTTGCCCAGAATGGATGC | GATGAACCGTGTCTGTGAGGC |
| miR6025 | orange1.1g005832m | AT1G06820.1 | Carotenoid isomerase | GGGACTGGCTCAAAAGGACTATG | TGGTGACCCAATCTCCCTAAAA |
|  | orange1.1g023118m | AT2G21940.4 | Shikimate kinase 1 | AGGCAGGGCATCACTGTCTTT | TTGCTATGGTGGTGGGCGTA |
| miR6214 | orange1.1g037661m | AT5G37380.4 | Chaperone DnaJ-domain superfamily protein | AGTTGGCAGACAAGGAAGCG | CCCCTGATGGTTTGGGTTTT |
| miR6260 | orange1.1g010903m | AT5G15130.1 | WRKY DNA-binding protein 72 | TACTCCTCCTCCCATTCCTCAA | CTCCAACATTCTGCTGACCGA |
|  | orange1.1g003752m | AT5G42480.1 | Chaperone DnaJ-domain superfamily protein | GAGTTGCCCAGAATGGATGC | GATGAACCGTGTCTGTGAGGC |
|  | orange1.1g041599m | AT1G49330.1 | Hydroxyproline-rich glycoprotein family protein | TTCTCGGCCATTACGTCCTC | TGTCCCTCAAGTCATACCCCAT |
|  | orange1.1g029026m | AT1G64650.1 | Major facilitator superfamily protein | AGGTGGAGGCATCTCATTATCTG | CCCTCAAGCCGCCTCTGTAA |
| miR7539 | orange1.1g002698m | AT2G42600.1 | Phosphoenolpyruvate carboxylase 2 | TCGTCCATCAAAGCGAAAGC | GACCCTAAAGAAAGGCCACAAA |
|  | orange1.1g020124m | AT2G01060.1 | MYB-like HTH transcriptional regulator family protein | CCAGGACAAGGCTGCCAAAG | CGATGCCCGAGATGAAGGAT |
| miR7841 | orange1.1g041450m | AT3G42640.1 | H^+^-ATPase 8 | CTATGCTCAGAGTGGAAAGGC | GCTCATTCAATTCACGGTTG |

*Actin* (AEK97331.1) was used as an internal standard and the reverse (5´→3´) and forward (5´→3´) primers were GCTTGGAGCAAGTGCTGTGATT and AGAACTATGAACTGCCTGATGGC, respectively.
